# Allicin‒Decorated FeO_1‐x_OH Nanocatalytic Medicine for Fe^2+^/Fe^3+^ Cycling‒Promoted Efficient and Sustained Tumor Regression

**DOI:** 10.1002/advs.202402801

**Published:** 2024-06-20

**Authors:** Zhongming Jie, Bingyan Xiong, Jianlin Shi

**Affiliations:** ^1^ Shanghai Tenth People's Hospital Shanghai Frontiers Science Center of Nanocatalytic Medicine School of Medicine Tongji University Shanghai 200072 P. R. China; ^2^ School of Physical Science and Technology ShanghaiTech University Shanghai 201210 P. R. China; ^3^ Shanghai Institute of Ceramics Chinese Academy of Sciences Research Unit of Nanocatalytic Medicine in Specific Therapy for Serious Disease Chinese Academy of Medical Sciences (2021RU012) Shanghai 200050 P. R. China; ^4^ Center of Materials Science and Optoelectronics Engineering University of Chinese Academy of Sciences Beijing 100049 P. R. China

**Keywords:** allicin, Fe(II, III) cycling, Fenton reaction, GSH oxidation, tumor therapy

## Abstract

In the tumor treatment by Fenton reaction‒based nanocatalytic medicines, the gradual consumption of Fe(II) ions greatly reduces the production of hydroxyl radicals, one of the most active reactive oxygen species (ROS), leading to much deteriorated therapeutic efficacy. Meanwhile, the ROS consumption caused by the highly expressed reduced glutathione (GSH) in the tumor microenvironment further prevents tumor apoptosis. Therefore, using the highly expressed GSH in tumor tissue to promote the Fe(III) reduction to Fe(II) can not only weaken the resistance of tumor to ROS attack, but also generate enough Fe(II) to accelerate the Fenton reaction. In view of this, an allicin‒modified FeO_1‐x_OH nanocatalyst possessing varied valence states (II, III) has been designed and synthesized. The coexistence of Fe(II)/Fe(III) enables the simultaneous occurrence of Fenton reaction and GSH oxidation, and the Fe(III) reduction by GSH oxidation results in the promoted cyclic conversion of Fe ions in tumor and positive catalytic therapeutic effects. Moreover, allicin capable of regulating cell cycle and suppressing tumor growth is loaded on FeO_1‐x_OH nanosheets to activate immune response against tumors and inhibit tumor recurrence, finally achieving the tumor regression efficiently and sustainably. This therapeutic strategy provides an innovative approach to formulate efficient antitumor nanomedicine for enhanced tumor treatment.

## Graphical abstract

The allicin‒modified FeO_1‐x_OH nanocatalyst has been designed and synthesized. The coexistence of Fe(II)/Fe(III) enables the simultaneous occurrence of Fenton reaction and GSH oxidation. Allicin plays a significant role in inhibiting the recurrence of tumors. The present nanocatalytic therapeutic strategy combines tumor regression and recurrence inhibition in a collaborative manner, effectively overcoming the problems encountered in traditional nanocatalytic drugs.

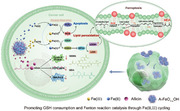

## 1 Introduction

Tumor, one of the most difficult diseases to conquer, has become one of the toughest obstacles for human beings to achieve healthy and long‒lasting development. During the tumor growth, the special microenvironment is gradually formed inside, such as oxygen deficiency, weak acidity, high concentrations of GSH (1–10 mM) and H_2_O_2_ (50–100 µM), and the stable expression of hypoxia‒inducible factor.^[^ ^1^ ^]^ This special microenvironment can not only prevent conventional drugs from reaching the tumor area resulting in poor treatment, but also trigger the immunosuppression in the tumor region.^[^ ^2^ ^]^ To solve these challenges for tumors treatment, a series of nanocatalytic medicines have been developed to introduce nanocatalytic reactions in tumor tissues for ROS productions and the activation of immune responses in response to tumor microenvironment.^[^ ^3^ ^]^

Iron‒based nanomaterials are widely used in tumor treatment thanks to their good biosafety and ease of decomposition, release and excretion of metabolites.^[^ ^4^ ^]^ Their core reaction for tumor treatment is Fenton reaction consisting of three steps (Equations (1), (2), (3)). In the step 1, Fe^2+^ rapidly reacts with the H_2_O_2_ generating Fe^3+^, ·OH, and OH^−^. The obtained ·OH of especially high oxidative potential and extremely short life‐time (10^−9^ s) is capable of damaging DNA and amino acids in proteins, and polyunsaturated fatty acid oxidation in lipids without significant spillover risk to surrounding normal tissue.^[^ ^5^ ^]^ Also, the oxidation of polyunsaturated fatty acid to lipid peroxide (LOOH) can be mediated by ROS, leading to the ferroptosis. In the steps 2 and 3, the produced Fe^3+^ will be reduced back to Fe^2+^ by H_2_O_2_ and HO_2_· as the reactant of the first step, accompanied by the release of O_2_ (Equations 2 and 3). However, the Fe^2+^ consumption in the step 1 is much faster than the Fe^2+^ production by step 2, resulting in the Fe^2+^ shortage and largely reduced hydroxyl radical production (Equations 1 and 2). In conventional iron‒based nanomedicines, the Fe ions show a single valence state and are unable to solve the problem of Fe^2+^ deficiency, leading to a decelerated Fenton reaction. Correspondingly, the therapeutic effect of nanocatalytic drugs on tumors will also be deteriorated correspondingly.

(1) $$Fe^{2+}+\;H_{2}O_{2}\to \;Fe^{3+}+\cdot \mathrm{OH}\;+\;OH^{-}$$

(2) $$Fe^{3+}+H_{2}O_{2}\to Fe^{2+}+HO_{2}\cdot +H^{+}$$

(3) $$Fe^{3+}+HO_{2}\cdot \to Fe^{2+}+O_{2}+H^{+}$$

(4) $$Fe^{3+}+\mathrm{GSH}\to Fe^{2+}+\mathrm{GS}\cdot +H^{+}$$

GSH, highly expressed in tumor microenvironment, exhibits strong antioxidant activities owing to its highly reducing thiol group that can react with oxidative free radicals.^[^ ^6^ ^]^ Resultantly, it will significantly counteract the efficacy of the antitumor treatment modalities by nanocatalytic medicine involving ROS productions, greatly undermining the therapeutic effect.^[^ ^7^ ^]^ Thus, there is a great significance for designing appropriate nano‒drugs to prevent the high expression of GSH in tumors and provide enough Fe(II) for Fenton reaction to promote the reaction kinetics. Fortunately, Fe(III)‒mediated oxidation of GSH and lipid peroxidation can directly promote tumor cell apoptosis.^[^ ^8^ ^]^ The reduction of Fe(III) back to Fe(II) in turn triggers the cyclic Fenton reaction for generating ROS.^[^ ^9^ ^]^ Additionally, the downregulation of defensive glutathione peroxidase 4 (GPX4) induced by GSH depleting prevents the conversion of highly toxic LOOH into low‒toxicity hydroxyl fatty acids (LOH), thus promoting the ferroptosis. Therefore, it is significant to develop a nanomedicine containing both Fe(III) and Fe(II) to facilitate the GSH oxidation and Fe(III) reduction back to Fe(II) for accelerating the Fenton reaction (Equation 4).

Nevertheless, a small amount of surviving tumor cells will exhibit corresponding drug resistance during the tumor therapy by nanomedicines, which can easily lead to tumor recurrence.^[^ ^10^ ^]^ This means that in order to achieve the goal of killing tumor cells, nanomedicines should not only be able to kill cancer cells, but also to prevent tumor recurrence concurrently. The Cyclin‒E and CDK2 genes, closely associated with the cell cycle, play a crucial role in inhibiting the proliferation of tumor cells.^[^ ^11^ ^]^ Allicin can inhibit the activity of DNA polymerase *β* (DNA Pol *β*), regulate cell cycle to inhibit tumor cell division and proliferation, and downregulate the expression of Cyclin‒E protein to inhibit the expression of the CDK2 gene.^[^ ^12^ ^]^ Therefore, in this study, allicin was employed to decorate FeO_1‐x_OH nanosheets, thus constructing a composite nanomedicines, named A‒FeO_1‐x_OH hereafter, in which FeO_1‐x_OH nanosheets act as a “sword” that can induce Fenton reaction, and the decorated allicin play a role of “shield” that is capable of inhibiting cancer recurrence.

**Figure 1:**
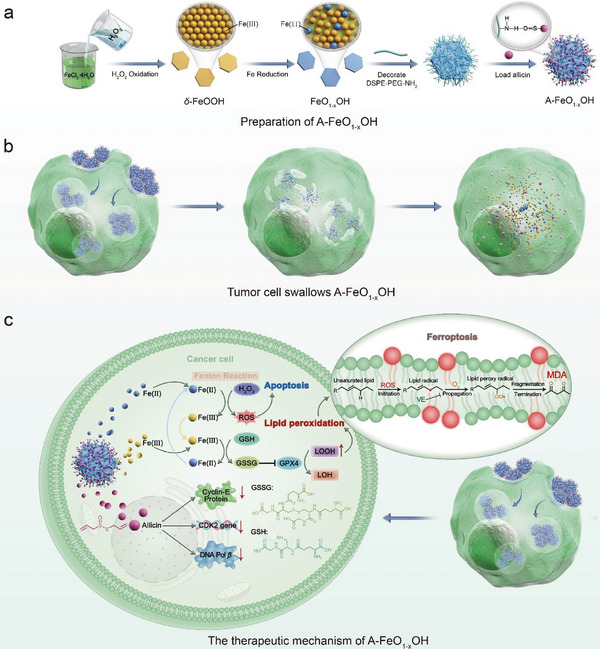
Schematic diagram for the synthesis of A‒FeO_1‐x_OH and its therapeutic mechanism. a) Schematics of the step‒by‒step synthesis procedure for A‒FeO_1‐x_OH. b) A‒FeO_1‐x_OH is phagocytosed by tumor cells, therein to release Fe(II,III) and allicin by degradation. c) The proposed mechanism of nanocatalytic tumor therapy by A‒FeO_1‐x_OH.

FeO_1‐x_OH nanosheets were synthesized through facile and efficient chemical precipitation and reduction methods (**Figure** **1**a). In a typical process, FeCl_2_ was oxidized by H_2_O_2_ to form *δ*‒FeOOH, which was then partially reduced to form FeO_1‐x_OH with mixed valences of Fe ions. Unlike conventional Fe‒based nanomedicines, Fe species in FeO_1‐x_OH are in the mixed form of both Fe(II) and Fe(III). After allicin decoration onto the FeO_1‐x_OH surface, the formed A‒FeO_1‐x_OH can be well‒phagocytosed by tumor cells, then degraded, releasing Fe(II, III) ions and allicin within the tumor tissue (Figure 1b). Subsequently, the Fe(II) triggers Fenton reaction producing a large amount of ROS to kill tumor cells. As a compensatory source of Fe(II), the released Fe(III) and the produced Fe(III) by the Fenton reaction of Equation 1 can be reduced by GSH and cycled back to Fe(II) through Equation 4, ensuring the continuous strengthening of Fenton reaction. Moreover, the immunity can be activated by FeO_1‐x_OH through cGAS‒STING pathway, and the tumor cell proliferation will be suppressed by allicin, diminishing the possibility of tumor recurrence (Figure 1c). Therefore, this strategy of combining both ROS production and cancer recurrence prevention provides a novel approach for the design of more advanced tumor nanomedicines for the efficient and sustained tumor treatment.

## 2 Results and Discussion

### 2.1 Synthesis and Characterization of A‒FeO_1‐x_OH

The *δ*‒FeOOH was prepared by a chemical precipitation and reduction method by using H_2_O_2_ oxidize FeCl_2_, followed by reduction of Fe(III) in *δ*‒FeOOH forming FeO_1‐x_OH containing both Fe(II)/Fe(III). The obtained FeO_1‐x_OH shows an orthorhombic crystalline structure, identical to that of *δ*‒FeOOH, indicating that the main crystalline structure remains unchanged after partial reduction of *δ*‒FeOOH to FeO_1‐x_OH. This result has been further confirmed by Raman spectroscopic data (Figure S1a and b, Supporting Information). Nevertheless, compared to *δ*‒FeOOH, the electronic state of FeO_1‐x_OH has undergone significant changes. The Fe 2p_3/2_ peak of FeO_1‐x_OH significantly shifts to a lower energy level (1.06 eV), while the O 1s peak shifts to a higher energy level (0.40 eV) (Figure S2a and b, Supporting Information). These results suggest that the formation of electron‒rich FeO_1‐x_OH is due to the shift of the electron cloud from the oxygen atom to the iron atom without the accompanying structural change. The Mossbauer spectrum data of FeO_1‐x_OH shows four peaks, confirming that the iron species in FeO_1‐x_OH has a mixed valence states of Fe(II) and Fe(III) with a Fe(II)/Fe(III) ratio of 5:1, which is consist with XPS data (Figure S2c and Table S1, Supporting Information). The as‒synthesized FeO_1‐x_OH presents a deeper brownish‒red color than that of *δ*‒FeOOH, due to the partial reduction of Fe(III) (Figure S3a, Supporting Information). Additionally, FeO_1‐x_OH exhibits significant Tyndall effect and excellent hydrophilicity, with a contact angle of 30.08° measured by a contact angle meter, favoring subsequent surface modification with highly biocompatible DSPE‒PEG‒NH_2_ for improved biocompatibility (Figure S3b, Supporting Information).^[^ ^13^ ^]^ Subsequently, the allicin was loaded on FeO_1‐x_OH, obtaining the target product A‒FeO_1‐x_OH. The percentage of allicin loaded on FeO1‐xOH determined by Inductively coupled plasma‒optical emission spectroscopy (ICP‒OES) is 12.21 m.% (Table S2, Supporting Information). From the N_2_ adsorption‐desorption isotherm, PEGylated FeO_1‐x_OH data displays typical mesoporous characteristics with mesoporous hysteresis loop and high specific surface area (151.6 cm^3^ g^−1^), which is beneficial for subsequently loading allicin into mesopore channels. After PEGylation, the pore size of FeO_1‐x_OH (15.34 nm) is smaller than that of *δ*‒FeOOH (45.78 nm), which may be caused by the decoration of DSPE‒PEG‒NH_2_ on the surface of FeO_1‐x_OH (Figure S4, Supporting Information).

Fourier transform infrared spectroscopy (FT‒IR) was applied to monitor the loading. The broadening of the ‒NH_2_ absorption peak indicates that allicin has been loaded onto FeO_1‐x_OH by hydrogen bonding (N‒H^…^O) with DSPE‒PEG‒NH_2_. Compared to FeO_1‐x_OH, the FT‒IR data of A‒FeO_1‐x_OH displays clear S = O absorption peaks, which is a typical absorption peak of allicin, demonstrating that allicin has been successfully loaded onto A‒FeO_1‐x_OH (Figure S5, Supporting Information).^[^ ^14^ ^]^ This inference is further confirmed by Zeta potential and Dynamic light scattering technique (DLS) measurements (Figure S6, Supporting Information). The long‒term stability of A‒FeO_1‐x_OH was observed for 30 days, and the particle size distribution and XRD data verify that A‒FeO_1‐x_OH can maintain long‒term stability in at least 30 days (Figure S7, Supporting Information).

**Figure 2:**
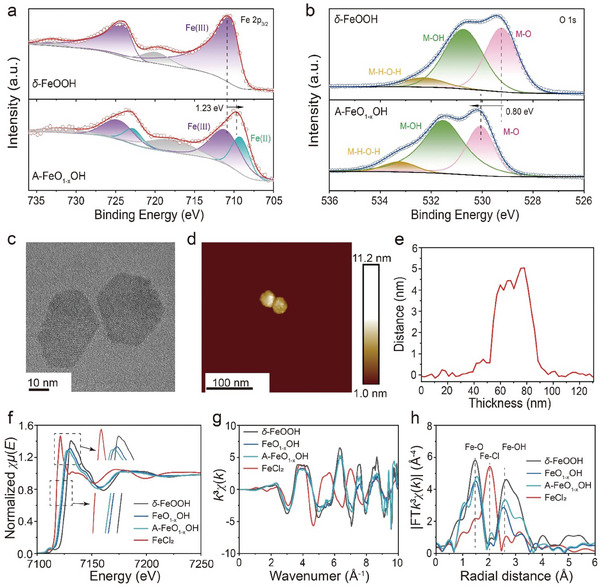
Characterizations of *δ*‒FeOOH, FeO_1‐x_OH and A‒FeO_1‐x_OH. a,b) XPS spectra for Fe 2p_3/2_ of *δ*‒FeOOH and A‒FeO_1‐x_OH a) and O 1s of *δ*‒FeOOH and A‒FeO_1‐x_OH b). c) TEM image of A‒FeO_1‐x_OH. d) AFM image of A‒FeO_1‐x_OH. e) Thickness distribution data of A‒FeO_1‐x_OH. f) XANES spectra of *δ*‒FeOOH, FeO_1‐x_OH, A‒FeO_1‐x_OH and FeCl_2_. g, h) *K* g) and *R* h) space extended EXAFS spectra of *δ*‒FeOOH, FeO_1‐x_OH, A‒FeO_1‐x_OH and FeCl_2_.

Attributing to the influences of allicin, A‒FeO_1‐x_OH exhibits S‒S (163 eV) and S = O (167 eV) bonds in XPS S 2p_3/2_ with an S element amount of 6.89%. From the XPS spectrum of *δ*‒FeOOH, the Fe 2p_3/2_ peak shifted by 1.23 eV toward the lower binding energy, while the O 1s peak shifted by 0.80 eV toward the higher binding energy (**Figure** **2**a and b; Figures S8,S9, Supporting Information). Moreover, the A‒FeO_1‐x_OH shows a slight increase of Fe(II) concentration (from 16.6% to 17.2%) compared to that of FeO_1‐x_OH, which is ascribed to the electron cloud shifts of electron‒rich allicin toward the iron core region, resulting in partial reduction of Fe(III) in FeO_1‐x_OH (Table S1, Supporting Information).^[^ ^15^ ^]^

Transmission electron microscopy (TEM) images show that *δ*‒FeOOH and FeO_1‐x_OH have a typical single‒crystalline hexagonal nanosheet structure of ≈40 nm in diameter (Figure S10a‒d, Supporting Information). From Spherical aberration‐corrected transmission electron microscopy (AC‒TEM) images, FeO_1‐x_OH clearly shows an atomic disorder phenomenon due to the partial reduction of Fe(III), compared to that of *δ*‒FeOOH (Figure S10e and f, Supporting Information). Similar to FeO_1‐x_OH, A‒FeO_1‐x_OH also possesses a hexagonal 2D nanosheet morphology, with a thickness of 4.4 nm determined by TEM and Atomic force microscopy (AFM), indicating that the loaded allicin will not affect the overall morphology of FeO_1‐x_OH (Figure 2c‒e; Figure S11, Supporting Information). Energy dispersive X‒ray (EDX) images show a uniform distribution of S element, illustrating that allicin has been uniformly decorated on the surface of A‒FeO_1‐x_OH (Figures S12,S13, Supporting Information).

In order to further determine the chemical state and bonding environment of the Fe species in FeO_1‐x_OH, and A‒FeO_1‐x_OH, Extended X‒ray absorption fine structure (EXAFS) and X‒ray absorption near‒edge structure (XANES) spectroscopies were used by selecting *δ*‒FeOOH and FeCl_2_ (with a typical Fe(II) chemical bonding environment) as references (Figure 2f,g). According to the *E*_0_ values from EXAFS samples, it can be inferred that the chemical valences of Fe species in A‒FeO_1‐x_OH and FeO_1‐x_OH are higher than that in FeCl_2_ (Fe(II)), but lower than that in *δ*‒FeOOH (Fe(III)), confirming mixed valence states of Fe in A‒FeO_1‐x_OH and FeO_1‐x_OH (the inset of Figure 2f and Table S3, Supporting Information). Compared with FeO_1‐x_OH, the Fe species in A‒FeO_1‐x_OH have much lower chemical valence states, demonstrating that the introduction of electron‒rich allicin has led to the electron enrichment around iron atom in A‒FeO_1‐x_OH and the increased amount of Fe(II), further confirming the existence of electronic interaction between FeO_1‐x_OH and allicin. By observing the peak intensity of EXAFS data, it is determined that due to the partial reduction of Fe(III) to Fe(II), A‒FeO_1‐x_OH shows further lowered crystallinity.^[^ ^16^ ^]^ From the Fourier transform EXAFS spectra, signals from *δ*‒FeOOH, FeO_1‐x_OH are assigned to Fe‒O (1.53 Å), Fe‒OH (2.51 Å) bonds, while the signals of Fe‒O (1.56 Å) and Fe‒OH (2.61 Å) are originated from A‒FeO_1‐x_OH (Figure 2h). Analogous to the previous tests, the structure of A‒FeO_1‐x_OH is similar to FeO_1‐x_OH, while both of them show the decreased valence and disordered lattice structure compared to those of *δ*‒FeOOH. Due to the decoration of allicin, the valence state and Fe(III) amount in A‒FeO_1‐x_OH further decreases. The changes in valence bond length are associated with the variation of iron valence states in the nanomedicine. In the EXAFS spectrum of FeCl_2_, the first coordination shell of Fe‒Cl can be identified at radial distances between 1.0 and 2.5 Å, indicating the existence of Fe‒Cl bonds (2.02 Å) (Figures S14,S15 and Table S4, Supporting Information). In addition, it can be seen that compared to *δ*‒FeOOH, FeO_1‐x_OH shows bond length changes of Fe‒O and Fe‒OH respectively from 1.47 to 1.50 Å and from 2.66 to 2.57 Å, which is also due to the partial reduction of Fe(III) to Fe(II) by electron‒rich allicin (Figure S16, Supporting Information).

### 2.2 ROS Production and GSH Oxidation

**Figure 3:**
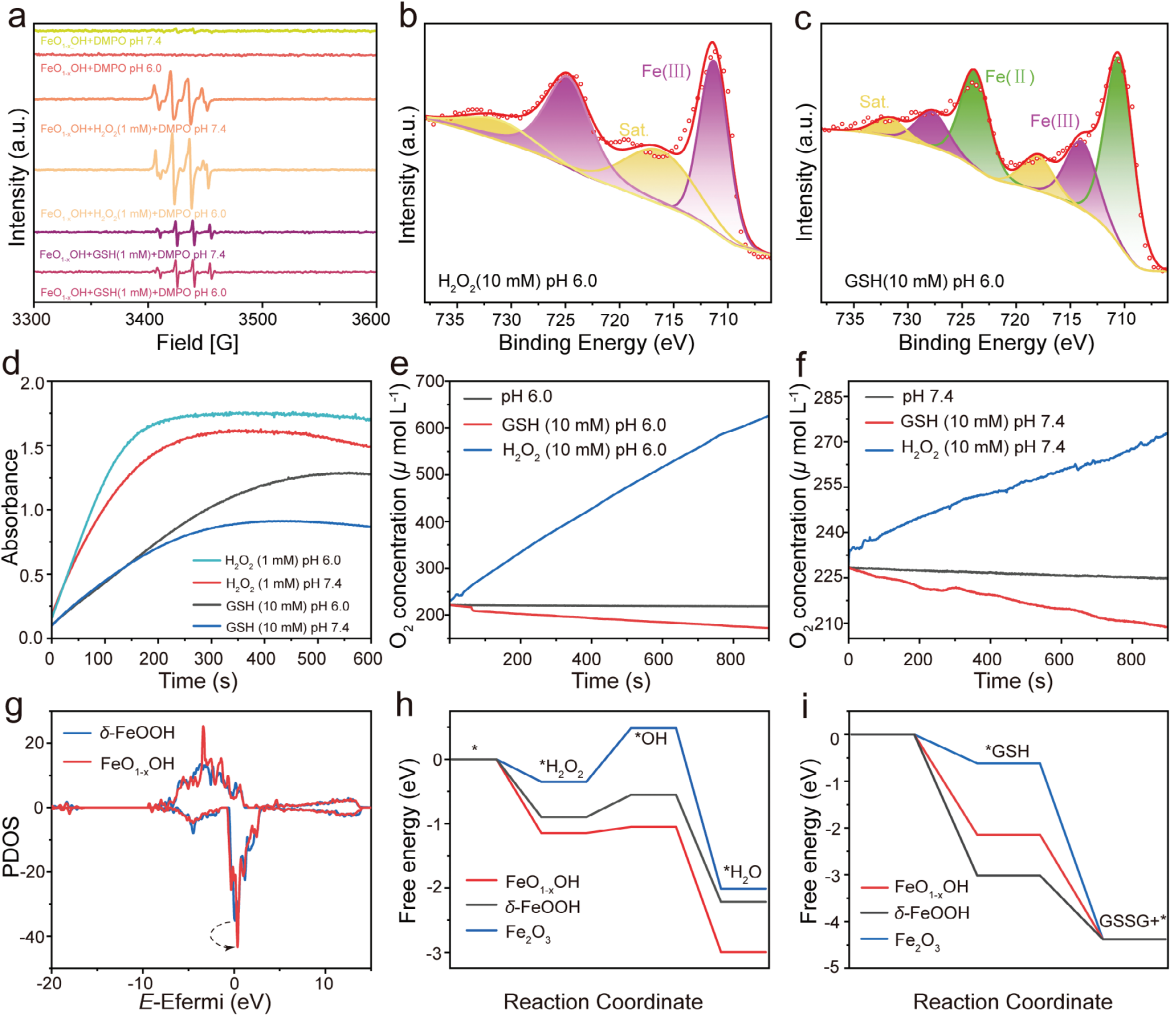
Catalytic performances of FeO_1‐x_OH. a) ESR spectra of ·OH generated during GSH oxidation or Fenton reaction‒induced H_2_O_2_ decomposition by FeO_1‐x_OH under different conditions, using DMPO as a trapping agent. b, c) Fe 2p_3/2_ XPS spectra for FeO_1‐x_OH after the reaction with H_2_O_2_ b) or GSH c). d) FeO_1‐x_OH catalyzes TMB color reactions of H_2_O_2_ and GSH under different conditions. e,f) The changes of O_2_ concentration during FeO_1‐x_OH‒catalyzed Fenton reaction for H_2_O_2_ decomposition and the oxidation of GSH (10 mM) in different media of pH 6.0 e) and pH 7.4 f). g) PDOS spectra of Fe 3d orbitals for FeO_1‐x_OH and *δ*‒FeOOH. h) Free energy diagrams of FeO_1‐x_OH, *δ*‒FeOOH, Fe_2_O_3_ for ·OH generation through Fenton reaction. i) Free energy diagrams of FeO_1‐x_OH, *δ*‒FeOOH, Fe_2_O_3_ for GSH oxidation reaction.

To verify whether FeO_1‐x_OH can oxidize GSH and catalyze the Fenton reaction or not, 5,5‒dimethyl‒1‒pyrroline‒N‒oxide (DMPO) was used as a typical ·OH capturing agent for electron spin resonance spectroscopy (ESR) measurement. After adding H_2_O_2_ or GSH to FeO_1‐x_OH solution at pH 6.0 and 7.4, distinguishable 1: 2: 2: 1 quadruple peak signals characteristic of ·OH are observed, confirming that the free radical has been generated during the Fenton reaction or the GSH oxidization to glutathione disulfide (GSSG) (**Figure** **3**a, Equations (4), (5), (6), (7), (8), (9), (10)).^[^ ^17^ ^]^ Moreover, compared to the situation at pH 7.4, the ROS signals are much intensified at pH 6.0, showing that FeO_1‐x_OH possesses enhanced catalytic activity in the slightly acidic environment of tumors.

(5) $$Fe^{2+}+\;\mathrm{GSH}\;\to \;\left[GS^{-}-Fe^{2+}\right]+H^{+}$$

(6) $$\left[GS^{-}-Fe^{2+}\right]\;+\;\mathrm{GS}\cdot \to \;\left[GS^{-}-Fe^{2+}-\mathrm{GS}\cdot \right]$$

(7) $$\left[GS^{-}-\;Fe^{2+}-\;\mathrm{GS}\cdot ]\;+\;\mathrm{GSH}\to \;[GS^{-}-Fe^{2+}-\mathrm{GSSG}\cdot^{-}\right]+H^{+}$$

(8) $$\begin{array}{ccc} {}[GS^{-} & - & Fe^{2+}-\mathrm{GSSG}\cdot^{-}]\;+\;O_{2}\to \;[GS^{-}-Fe^{2+}]\;+\cdot {O_{2}}^{-}+\mathrm{GSSG} \end{array}$$

(9) $$2H^{+}+\;Fe^{2+}+\cdot {O_{2}}^{-}\to \;Fe^{3+}+\;H_{2}O_{2}$$

(10) $$H_{2}O_{2}+\;Fe^{2+}\to Fe^{3+}+\;OH^{-}+\cdot \mathrm{OH}$$

After reacting with H_2_O_2_ (simulating the Fenton reaction), Fe ions in the FeO_1‐x_OH become almost completely +3 charged, suggesting the successful initiation of Fenton reaction by FeO_1‐x_OH (Figure 3b). While after reaction with GSH, the proportion of Fe (II) in the FeO_1‐x_OH increases dramatically, affirming that a fraction of Fe(III) has participated in oxidizing the GSH to GSSG and converted back into Fe(II) in this reaction system (Figure 3c). These results indicate that FeO_1‐x_OH can simultaneously oxidize GSH and catalyze the Fenton reaction because of the co‐existence of Fe(II) and Fe(III) in this nanomedicine. The activity of FeO_1‐x_OH to catalyze the Fenton reaction and produce ·OH was detected by methylene blue (MB) degradation, chromogenic reaction of 3,3,5,5‒tetramethylbenzidine (TMB) under the presence or absence of GSH. The absorbance of the solution at 660 nm decreased significantly, being 69% of the original value in 8 min, which is consistent with the previous results, i.e., FeO_1‐x_OH is active for both ROS production via Fenton reaction and GSH oxidation by Fe(III) in FeO_1‐x_OH, and the kinetics of the Fenton reaction for ROS production is faster than GSH oxidation due to the Fe(II) supply from the Fe(III) reduction by GSH (Figure S17, Supporting Information). Subsequently, we used TMB hydrochloride as an indicator of ROS production which can oxidize TMB hydrochloride from transparency to blue. The results also indicate the Fenton reaction producing ROS is much more efficient than the GSH oxidation, and the color change at pH 6.0 is faster than that at pH 7.4, indicating that the promoted reaction in acidic environment is beneficial for the therapy of weakly acidic tumor (Figure 3d; Figure S18, Supporting Information).

To measure the GSH oxidation activity of FeO_1‐x_OH, 5,5′‒dithiobis (2‒nitrobenzoic acid) (DTNB) was used as an indicator to react with the thiol groups in GSH, forming a yellow product. In the reaction time period of 8 min, the GSH concentration decreases to 53% of the initial value, verifying that FeO_1‐x_OH exhibits excellent GSH oxidizing activity (Figure S19, Supporting Information).

Afterward, an active oxygen electrode was applied to measure the changes in dissolved oxygen level in the reaction solution over time. Compared with the blank solution, the oxidation of GSH by FeO_1‐x_OH is accompanied by the consumption of O_2_ (Equation 8), while the catalytic Fenton reaction of H_2_O_2_ releases abundant O_2_ accompanying ROS production (Equations 1 and 3), therefore this experiment confirms the prevailing production of ROS by the Fenton reaction. Besides, the GSH oxidation by the nanomedicine takes place much faster and more thoroughly in an acidic environment (pH 6.0) than in neutral condition (Figure 3e,f).

The comparison of cyclic voltammetry curves of H_2_O_2_ and GSH at the same concentration and varied pH values were performed to evaluate the reaction kinetics of GSH oxidation and Fenton reaction. The results show that the current densities at pH 6.0 in almost entire voltage scanning window for reducing H_2_O_2_ are typically higher than that for oxidizing GSH, confirming the Fenton reaction dominates the whole solution reaction under the promotion by GSH oxidation (Figure S20, Supporting Information).

Further, Vienna ab initio simulation package (VASP) was employed for density functional theory (DFT) calculations. Partial density of state (PDOS) results calculated based on HSE06 functions at zero Fermi energy level indicate that, compared to *δ*‒ FeOOH, FeO_1‐x_OH shows an enhanced charge density distribution at spin down Fe 3d orbital near the Fermi energy level, which is beneficial for electron transport, facilitating the reaction activity enhancement (Figure 3g). From the Gibbs free energy calculation, the Fenton reaction catalyzed by both *δ*‒FeOOH (−0.55 eV) and FeO_1‐x_OH (−1.05 eV) are spontaneous (*ΔG* < 0), and FeO_1‐x_OH exhibits a faster and more thorough reaction pathway (Figure 3h). However, the reaction catalyzed by Fe_2_O_3_ is kinetically reluctant and requires external energy supplementation (0.48 eV), while GSH oxidation forming GSSG is a thermodynamic spontaneous process (*ΔG* < 0). The adsorption energy of *GSH on FeO_1‐x_OH (−3.01 eV) is lower than that on *δ*‒FeOOH (−2.14 eV) and Fe_2_O_3_ (−0.62 eV), indicating the adsorption of *GSH on FeO_1‐x_OH is predominant (Figure 3i). The Fe(II)‒containing FeO_1‐x_OH provides the adsorption sites, promoting the oxidation conversion of GSH to GSSG. All calculation results indicate that compared with other reference samples, FeO_1‐x_OH shows the highest activity in catalyzing Fenton reaction and GSH oxidization (Figure S21, Supporting Information).

### 2.3 Degradation and Release of A‒FeO_1‐x_OH and Redox Reactions between Fe(II) and Fe(III) in Tumor Cell Environments

**Figure 4:**
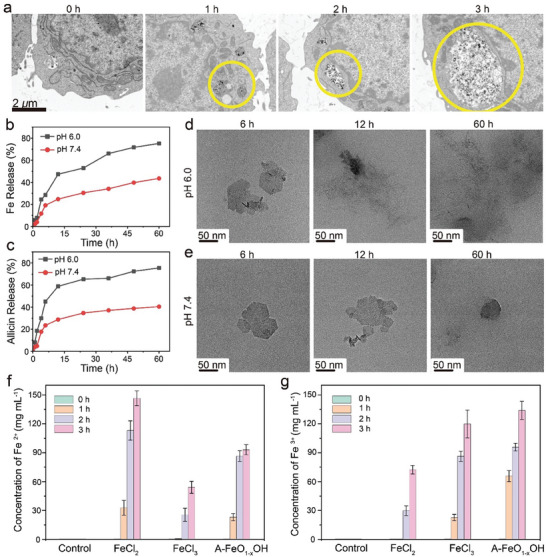
Phagocytosis and degradation of A‒FeO_1‐x_OH by tumor cells. a) Observation of time‒dependent phagocytosis of A‒FeO_1‐x_OH by 4T1 tumor cells using Bio‒TEM. b,c) Accumulated release curves of Fe b) and allicin c) from A‒FeO_1‐x_OH under different pH environments. d,e) TEM observations of the degradation of A‒FeO_1‐x_OH in different pH environments at pH 6.0 d) and pH 7.4 e). f,g) IC‒ICP‒OES data at different time intervals after co‒incubations of FeCl_2_, FeCl_3_, or A‒FeO_1‐x_OH with 4T1 cells.

In order to verify that A‒FeO_1‐x_OH can be phagocytosed and degraded by tumor cells, A‒FeO_1‐x_OH was co‒incubated with 4T1 cells, and the cell pellets were collected at different time points. From the biological transmission electron microscopic (Bio‒TEM) images, it is clear that A‒FeO_1‐x_OH can be well phagocytosed by 4T1 tumor cells through cellular vesicle cytosis, as further validated by statistical result of Fe elemental mapping (**Figure** **4**a; Figure S22, Supporting Information). As the reaction proceeds, the cumulative amount of A‒FeO_1‐x_OH in 4T1 cells gradually increases, ensuring the efficient antitumor treatments.

Then, A‒FeO_1‐x_OH was immersed in PBS of varied pH levels for evaluating the biodegradability. Under a mildly acidic environment (pH 6.0), the release rates of iron and allicin are higher than those at neutral pH 7.4 in the time course (Figure 4b,c). The TEM images of A‒FeO_1‐x_OH at different time points further illustrate the differentiated release rates at pH 6.0 and pH 7.4 (Figure 4d and e). It can be seen that, the unique hexagonal morphology of A‒FeO_1‐x_OH has been broken in 6 h in pH 6.0 environment, which is almost unchanged at pH 7.4. In 12 h, A‒FeO_1‐x_OH has been mostly degraded at pH 6.0, but the nanosheet shows almost no degradation at pH 7.4. In 60 h, no nanosheets of A‒FeO_1‐x_OH can be observed in pH 6.0 medium compared to that in pH 7.4 medium. These results suggest that the degradation rate of A‒FeO_1‐x_OH is significantly faster in weakly acidic medium than that in neutral pH environment, which is beneficial for nanocatalytic therapy only in tumor environments without damaging normal cellular tissues of neutral pH.^[^ ^18^ ^]^

After being phagocytosed by 4T1 cells, the concentrations of Fe^2+^ and Fe^3+^ in the solution were detected by Ion chromatography coupled with inductively coupled plasma‒optical emission spectroscopy (IC‒ICP‒OES) to monitor the degradation of A‒FeO_1‐x_OH. After the co‒incubation of A‒FeO_1‐x_OH, FeCl_2_ or FeCl_3_ with 4T1 cells in PBS, the cells were collected and lysed, and then the Fe^3+^/Fe^2+^ ion concentrations were tested in obtained decomposition solution. From the data of FeCl_2_ group, the concentrations of Fe^3+^ and Fe^2+^ in the cells increases over time, and the concentration of Fe^2+^ is much higher than that of Fe^3+^. The presence of Fe^3+^ in cells indicates that Fe^2+^ has played a role in catalyzing Fenton reaction after being absorbed by 4T1 cells, leading to partial oxidation of Fe^2+^ to Fe^3+^. Meanwhile, the addition of FeCl_3_ increases the concentrations of intracellular Fe^3+^ and Fe^2+^, and these Fe^2+^ ions are derived from the reduction of Fe^3+^ by GSH. According to the results of FeCl_2_ and FeCl_3_ groups, it can be known that A‒FeO_1‐x_OH induces both Fenton reaction and GSH oxidation reaction after being phagocytosed by the tumor cells, resulting in the simultaneous increases in Fe^2+^ and Fe^3+^ concentrations (Figure 4f,g). Considering that Rhodamine B and FerroOrange can bind respectively with intracellular Fe^3+^ and Fe^2+^, producing red and orange fluorescence accordingly, they can be used to detect the distributions of intracellular Fe^2+^ and Fe^3+^ in cells. Using the same method as IC‒ICP‒OES measurement, 4T1 cells were cultured and stained, strong orange fluorescence was observed, indicating that compared to the control group, the A‒FeO_1‐x_OH and FeCl_2_ groups show higher intracellular Fe^2+^ amounts. After the treatments with A‒FeO_1‐x_OH and FeCl_3_, strong red fluorescence can be observed in both groups, confirming the intracellular Fe^3+^ amounts were enhanced after the treatments (Figure S23, Supporting Information). These results further confirm A‒FeO_1‐x_OH can be degraded after being phagocytosed by 4T1 cells. After co‒culturing with FeCl_3_ for a period of time, the presence of Fe^2+^ in the solution was originated from GSH oxidation in tumor cells. Meanwhile, the Fenton reaction triggered by H_2_O_2_ in tumor cells results in the transformation from Fe^2+^ to Fe^3+^, which is confirmed by the presence of Fe^3+^ in the solution after co‒culturing with FeCl_2_ for a period of time. Similarly, according to the results of the FeCl_2_ and FeCl_3_ groups, it can be seen that after adding A‒FeO_1‐x_OH, a fraction of Fe^2+^ ions can be oxidized to Fe^3+^ by Fenton reaction, which afterwards can be reduced back to Fe^2+^ by GSH oxidation in the tumor microenvironment. Therefore, it can be concluded that A‒FeO_1‐x_OH has excellent capabilities to promote both Fenton reaction and GSH oxidation.

### 2.4 A‒FeO_1‐x_OH Mediated Ferroptosis and Apoptosis Mechanism

**Figure 5:**
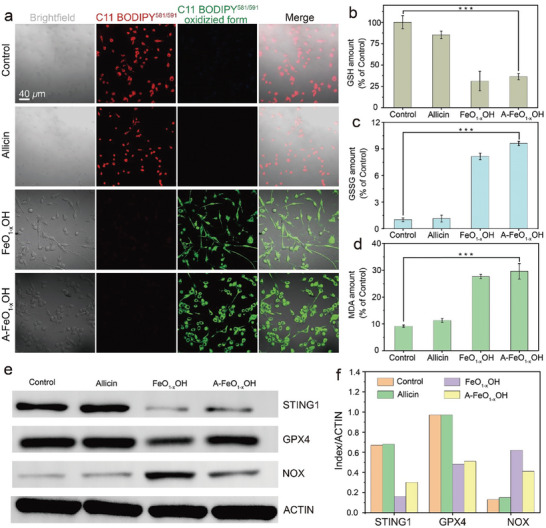
A‒FeO_1‐x_OH induces ferroptosis in tumor cells. a) CLSM images of 4T1 cells incubated with allicin, FeO_1‐x_OH and A‒FeO_1‐x_OH after stained with C11‒BODIPY^581/591^ dye. b‒d) GSH b), GSSG c) and MDA d) levels in 4T1 tumor cells after the treatment by allicin, FeO_1‐x_OH or A‒FeO_1‐x_OH. The results are reported as means standard deviation (*n* = 3). *p < 0.05, **p < 0.01, ***p < 0.001. e, f) WB analysis of STING1, GPX4 and NOX proteins under varied conditions.

As well known, Fe(III) can not only oxidize GSH and promote lipid peroxidation, but also directly oxidize lipids, promoting the occurrence of ferroptosis.^[^ ^19^ ^]^ In order to explore the mechanism of A‒FeO_1‐x_OH mediated ferroptosis and apoptosis, Confocal laser scanning microscope (CLSM) was used to observe the state of 4T1 cells incubated with A‒FeO_1‐x_OH, allicin and FeO_1‐x_OH for 3 h and C11‒bodipy^581/591^ dye was used as a lipid peroxidation sensor. Upon oxidation of C11‒bodipy,^581/591^ the maximum emission wavelength shifted from 590 nm (red) to 510 nm (green). As shown in **Figure** **5**a, green fluorescence signal of the A‒FeO_1‐x_OH group is significantly intensified, while on the contrary, the red fluorescence signal is significantly weakened. Accordingly, the control group (only 4T1 cells) shows no lipid peroxidation emitting red fluorescence signal. The fluorescence intensity of A‒FeO_1‐x_OH is similar to that of FeO_1‐x_OH, and that from allicin group is also similar with that of the control group, suggesting A‒FeO_1‐x_OH is capable of inducing the ferroptosis of 4T1 cells thanks to the high catalytic activity of FeO_1‐x_OH. The GSH/GSSG kit was used to detect GSH and GSSG during the cellular ferroptosis. The 4T1 cells were incubated for 6 h with A‒FeO_1‐x_OH, and the resultant GSH concentration of this group decreased to 36% compared to the control group of 100%. While the GSSG concentration in A‒FeO_1‐x_OH group is 9.6 times higher than that of the control group, due to the strong oxidation activity of the Fe(III)‒containing A‒FeO_1‐x_OH, confirming the occurrence of ferroptosis. Because of the ineffectiveness of allicin in inducing ferroptosis, the GSH and GSSG levels in the FeO_1‐x_OH group are similar to those in the A‒FeO_1‐x_OH group. These results indicate that FeO_1‐x_OH in A‒FeO_1‐x_OH is the predominant component for the ferroptosis (Figure 5b,c).

During the ferroptosis, lipid peroxide was eventually decomposed into small molecular by‒products containing malondialdehyde (MDA).^[^ ^20^ ^]^ Thus, MDA amount can be measured to verify the ferroptosis. Similarly, 4T1 cells were incubated with allicin, FeO_1‐x_OH and A‒FeO_1‐x_OH for 6 h at the dosages indicated above, and the resultant MDA amounts were determined using an MDA assay kit. Compared with the control group, the MDA amount in the A‒FeO_1‐x_OH group significantly increases by 3.2 times, which is close to that in FeO_1‐x_OH group, verifying that MDA was produced during the ferroptosis by FeO_1‐x_OH in the A‒FeO_1‐x_OH group (Figure 5d).

**Figure 6:**
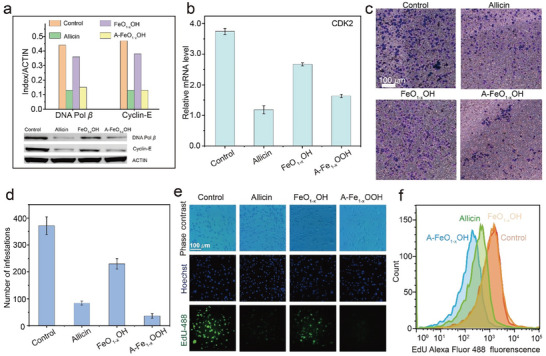
The inhibitory effect of allicin from A‒FeO_1‐x_OH on tumor cell proliferation. a) WB analysis of DNA Pol *β*, Cyclin‒E proteins under varied conditions. b) RT‒qPCR analysis of CDK2 expression levels in 4T1 cells treated with allicin, FeO_1‐x_OH or A‒FeO_1‐x_OH. The results are reported as means standard deviation (*n* = 3). c,d) Micrographs of infiltrated and metastasized tumor cells after treated with allicin, FeO_1‐x_OH or A‒FeO_1‐x_OH for 12 h and then stained with crystal violet before the measurement c), and the corresponding quantitative results d). The results are reported as means standard deviation (*n* = 3). e,f) CLSM images of EdU and Hoechst‒stained 4T1 cells after incubation in allicin, FeO_1‐x_OH or A‒FeO_1‐x_OH media for 6 h e) and corresponding flow cytometry data f).

The expression of ferroptosis–related proteins induced by A‒FeO_1‐x_OH was evaluated through Western blotting (WB) technique. Compared with the control group, the gray level of STING1 and GPX4 protein bands in both FeO_1‐x_OH and A‒FeO_1‐x_OH decreases significantly, while that of NOX protein band increases, indicating down‒regulated expressions of STING1 and GPX4 proteins, but increased NOX expression. The WB experimental results also prove that A‒FeO_1‐x_OH can induce significant ferroptosis in tumor cells (Figure 5e,f).^[^ ^21^ ^]^ All results above are in accordance with the GSH, GSSG and MDA concentration measurements. Hence, it can be concluded that A‒FeO_1‐x_OH can oxidize both GSH and lipids, leading to the ferroptosis of tumor cells. The 4T1 cells were co‒incubated with allicin, FeO_1‐x_OH or A‒FeO_1‐x_OH for 24 h. The protein bands of Cyclin‒E and DNA Pol *β* for allicin and A‒FeO_1‐x_OH groups have turned gray levels, indicating that allicin can inhibit the activity of DNA Pol *β* and downregulate the expression of Cyclin‒E protein (**Figure** **6**a).

The expression of CDK2 gene in 4T1 cells was detected by Real‒time fluorescence quantitative PCR (RT‒qPCR). As in previous experiments, 4T1 cells were incubated with different nanomedicines for 6 h before measurement. The expression level of the CDK2 gene in the allicin group is only 31% that of the control group, verifying that allicin can inhibit the expression of the CDK2 gene in cells. The decrease in CDK2 expression induced by allicin is originated from DNA damage caused by ·OH generated from Fenton catalytic reaction in the weakly acidic microenvironment of tumor cells.

Compared with the control group, the expression level of CDK2 in A‒FeO_1‐x_OH group is 43%, in comparison to 68% in the FeO_1‐x_OH group (Figure 6b and Table S5, Supporting Information). Hence, A‒FeO_1‐x_OH shows a significant inhibitory effect on CDK2 expression, attributing to the release of allicin in cells.

The inhibitory effect of allicin on tumor cell proliferation was further explored by performing cell invasion assays. 4T1 cells were stained with crystal violet and photographed for counting. Similar to allicin treatment, the number of infiltrated 4T1 cell largely decreased after A‒FeO_1‐x_OH treatment for 12 h, much more significant than FeO_1‐x_OH treatment (Figure 6c and d). This indicates that A‒FeO_1‐x_OH shows an inhibitory effect on the proliferation of tumor cells, benefiting from the loaded allicin. In addition, EdU (5‒ethynyl‒2′‒deoxyuridine) can replace thymidine in DNA synthesis, which can be excited by green fluorescence at 488 nm and observed through the CLSM. The 4T1 cells were treated with allicin, FeO_1‐x_OH or A‒FeO_1‐x_OH for 6 h. Different from FeO_1‐x_OH treatment, the green fluorescence signal of cells treated with A‒FeO_1‐x_OH or allicin is significantly reduced compared to that of the control group. Flow cytometry results also confirm the above phenomenon, demonstrating that allicin can regulate the cell cycle and inhibit the replication of tumor cell DNA, therefore playing a crucial role in suppressing tumor cell proliferation (Figure 6e,f). Therefore, it is not difficult to conclude that the inhibitory effect of allicin on tumor cell proliferation and nanocatalytic killing effect of FeO_1‐x_OH on tumor cells can cooperatively enhance the antitumor effect.

Subsequently, the activity of A‒FeO_1‐x_OH to catalyze the Fenton reaction and produce high cytotoxic ·OH in tumor cells was further evaluated using DCFH‒DA probe and DAPI dye. After A‒FeO_1‐x_OH or FeO_1‐x_OH treatment, the laser stimulated green fluorescence signal is enhanced, and the cytometry signal shifts to the right, indicating a significant intensification of the intracellular DCF green fluorescence signal (Figure S24, Supporting Information). In contrast, no green fluorescence is found in allicin and control groups, suggesting that due to the presence of Fe(II) and Fe(III), A‒FeO_1‐x_OH and FeO_1‐x_OH can induce Fenton reaction to produce ROS. The cytotoxic effects of A‒FeO_1‐x_OH, FeO_1‐x_OH, and allicin were evaluated by flow cytometry, CLSM, and CCK‒8 colorimetry. From the CCK‒8 colorimetric data, A‒FeO_1‐x_OH shows the strongest toxic effect on cells at a concentration of 250 µ*g* mL^−1^, while the allicin shows a significant toxic effect at 0.5 g L^−1^ (Figures S25 and S26, Supporting Information). The level reduction of intracellular ATP by mitochondrial damage is another marker of apoptosis.^[^ ^22^ ^]^ Different with allicin, the ATP level of 4T1 cells treated with the same concentration of A‒FeO_1‐x_OH or FeO_1‐x_OH is significantly reduced compared to that of the control group, illustrating that the cell apoptosis is derived from mitochondrial damage caused by FeO_1‐x_OH‒induced ROS (Figure S27, Supporting Information).^[^ ^23^ ^]^ CLSM was used to observe the extent of apoptosis of nanomedicine‒treated 4T1 cells stained with Calcein‒AM/PI. The 4T1 cells treated with allicin, FeO_1‐x_OH, or A‒FeO_1‐x_OH all present strong red PI fluorescence signals, while the green fluorescence signals of Calcein AM sharply weaken, once again demonstrating the strong toxicity of A‒FeO_1‐x_OH, FeO_1‐x_OH, and allicin on 4T1 cells. Annexin V‒FITC/PI dye flow cytometry data also show that allicin, FeO_1‐x_OH and A‒FeO_1‐x_OH exhibit apoptotic activity against the tumor cells, which is consistent with the above conclusion (Figures S28 and S29, Supporting Information). From the WB results and compared with the control group, the A‒FeO_1‐x_OH group shows the increased grayscale levels of C‒Caspase3, Caspase3, Caspase9, and Bax bands, while Bcl‒2 exhibits the decrease levels, indicating the gradually strengthened cell apoptosis. Therefore A‒FeO_1‐x_OH shows a strong effect in inducing cell apoptosis (Figure S30, Supporting Information).^[^ ^24^ ^]^ From the Bio‒TEM images, local membrane rupture, slight atrophy of nucleus and swelling of organelles can be observed in cells treated with allicin. After treatment with FeO_1‐x_OH, a certain extent of nucleus solidification, local rupture of cell membrane and swelling of organelles can also be observed. Comparatively, cells treated with A‒FeO_1‐x_OH exhibit much more significant apoptotic toxicity than allicin and FeO_1‐x_OH, as judged from the prevailing coagulation of nucleus and existences of lipid malnutrition droplets and autophagic lysosomes in large quantities (Figure S31, Supporting Information). Hence, A‒FeO_1‐x_OH can induce both Fenton reaction and lipid peroxidation through intracellular release of Fe(II) ions for the apoptosis and ferroptosis of the cancer cells, and the released allicin from the nanomedicine in mildly acidic tumor tissues regulates cell cycle and inhibits the proliferation of tumor cells.

### 2.5 A‒FeO_1‐x_OH Promotes Indirect Antitumor Effects

**Figure 7:**
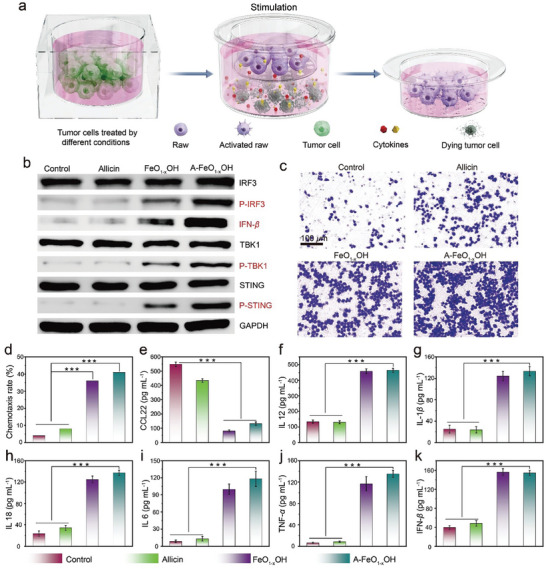
In vitro stimulation of immune response of RAW264.7 incubated with 4T1 cells upon A‒FeO_1‐x_OH treatment. a) Diagram of the transwell experiment employed. b) WB results of cGAS‒STING pathway proteins expressed in macrophages. c) Microscopic images of allicin, FeO_1‐x_OH and A‒FeO_1‐x_OH treated chemotaxis RAW264.7 (purple) stained with crystal violet. d) Chemotaxis rate of RAW264.7 macrophages induced by 4T1 cells under various treatments. e‒k) Concentrations of CCL22 e), IL 12 f), IL‒1*β* g), IL 18 h), IL 6 i), TNF‒*α* j), and IFN‒*β* k) in RAW264.7 macrophage cell‒cultured supernatants, as measured by ELISA, including the control, allicin, FeO_1‐x_OH and A‒FeO_1‐x_OH treatment groups (*n* = 3, *p < 0.05, **p < 0.01, and ***p < 0.001). The results are reported as means standard deviation.

As previously reported, the cGAS mediates the innate immune cGAS‒STING pathway by sensing tumor‒derived DNA, which can be activated by Fe‒based nanomaterials, and then promote interferon beta (IFN‒*β*) secretion, activating the indirect antitumor effects.^[^ ^25^ ^]^ To verify the cGAS‒STING pathway, a transwell experimental system was constructed to clearly demonstrate this immune activation pathway (**Figure** **7**a). WB analysis was conducted to examine the phosphorylation and expression of relevant factors, including phosphorylated STING (p‒STING), tank‒binding kinase 1 (TBK1), phosphorylated TBK1 (p‒TBK1), interferon regulatory factor 3 (IRF3), phosphorylated IRF3 (p‒IRF3), and their downstream markers in the cGAS‒STING pathway, as well as IFN‒*β* protein secretion level. Compared with the control group, significant upregulations of p‒STING, p‒TBK1, and p‒IRF3 were observed in the RAW264.7 macrophage lysates of the A‒FeO_1‐x_OH group, whereas non‒phosphorylated markers remain basically unchanged, similar to the results of the FeO_1‐x_OH group, demonstrating that A‒FeO_1‐x_OH is able to promote indirect antitumor immunity and activate the cGAS‒STING pathway originated from FeO_1‐x_OH (Figure 7b; Figure S32, Supporting Information).

The macrophage RAW264.7 cells were co‒cultured with the 4T1 cells to assess the immunoreactive behavior of different nanomedicine treatments for 24 h. The therapeutic effect of allicin treatment resembles that of the control group, without significant migration of RAW264.7. After the treatment with A‒FeO_1‐x_OH, however, the migration of macrophages shows a significant enhancement, suggesting that A‒FeO_1‐x_OH is capable of promoting the immune cell infiltration in the tumor microenvironment due to the presence of Fe(II) and Fe(III) (Figure 7c,d). A‒FeO_1‐x_OH treatment leads to the downregulation of the M2‒related cytokine CCL22 and upregulation of the M1‒related cytokine IL‒12. The ELISA results show that compared with the control group, the expression of the cGAS‒STING pathway and associated antitumor cytokines, such as IL‒1*β*, IL‒18, IL‒6, TNF‒*α*, and IFN‒*β*, are significantly enhanced, indicating the effective antitumor immunity activation (Figure 7e‒k). Additionally, similar to the FeO_1‐x_OH group, the expression of the M1‒associated marker CD86 in the A‒FeO_1‐x_OH group is markedly upregulated, while the expression of M2‒associated marker CD206 is down‒regulated, also suggesting antitumor immunity stimulations by the Fe(II)/Fe(III) cycling from A‒FeO_1‐x_OH (Figure S33, Supporting Information). Furthermore, it is worth noting that A‒FeO_1‐x_OH is capable of generating a large amount of ROS in tumor cells, which can stimulate immunogenic cell death (ICD) by inducing endoplasmic reticulum (ER) stress.^[^ ^26^ ^]^ Therefore, we used WB assay to assess whether A‒FeO_1‐x_OH can re‒initiate endoplasmic reticulum stress, by measuring the expressions of endoplasmic reticulum stress‒related proteins such as C/EBP homologous protein (CHOP) and eukaryotic initiation factor 2*α* (eIF2*α*). As expected, compared with the control group, the expressions of p‒eIF2*α* (phosphorylated form of eIF2*α*) and CHOP are significantly up‐regulated in A‒FeO_1‐x_OH group and slightly higher than that in FeO_1‐x_OH group, as A‒FeO_1‐x_OH mainly produces ROS from its FeO_1‐x_OH component (Figure S34, Supporting Information). The expressions of p‒eIF2*α* and CHOP in allicin and control groups are similar with each other and much lower than that in the FeO_1‐x_OH and A‒FeO_1‐x_OH groups, as allicin cannot trigger ROS production. This result indicates that A‒FeO_1‐x_OH can effectively induce ER stress, which lays the foundation for subsequent ICD to promote dendritic cells (DCs) maturation and T cell activation for chemo‐immunotherapy of tumors.

### 2.6 Evaluation of Therapeutic Effect in Mice

To conduct the tumor nanocatalytic therapy, the hemocompatibility and histocompatibility of the allicin, FeO_1‐x_OH, and A‒FeO_1‐x_OH were preliminarily evaluated. Seven‒week‒old Institute of Cancer Research (ICR) mice were categorized into four groups randomly: control, allicin, FeO_1‐x_OH and A‒FeO_1‐x_OH groups. The weights of the mice were recorded for 30 consecutive days and the serum and plasma were collected for routine blood biochemical examinations in the 30th day. The heart, liver, spleen, lung, and kidney samples were stained with hematoxylin‒eosin (H&E) for observation. The steady increment of weight, with no abnormality or lesion observed in blood routine, blood biochemistry and H&E section histology, indicates that A‒FeO_1‐x_OH and its related species possess excellent biosafety and can be used for further in vivo tumor treatment (Figure S35, Supporting Information).

Next, tumor‒bearing mice were used to evaluate the biological targeting of A‒FeO_1‐x_OH to tumor regions and its metabolism in mice, A‒FeO_1‐x_OH modified with Cy5.5 were tail‒vein administrated. The infrared signal at 703 nm from Cy5.5 gradually appears in the tumor area of the mice in 30 min, then reaches the maximum in 6 h, and becomes disappeared in 24 h. The main organs and tumors in mice were collected for fluorescence measurements. The fluorescence signal can be found mainly in tumor and kidney in 24 h, confirming that A‒FeO_1‐x_OH can be well enriched and retained in tumor environment, and most of them in normal organs can be excreted from the body through the metabolic system of the mouse in 24 h (Figure S36, Supporting Information).

**Figure 8:**
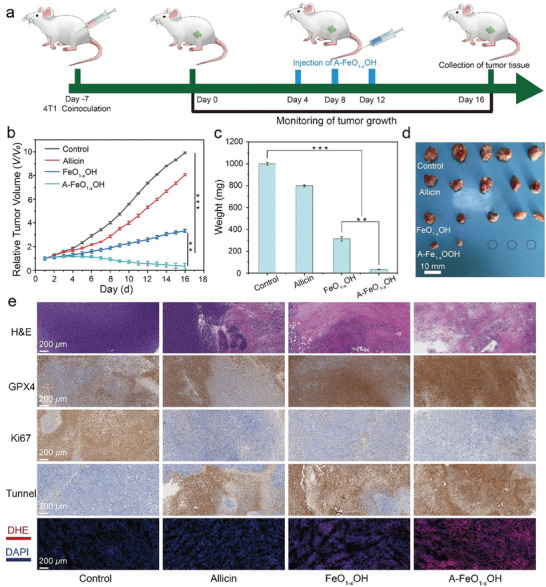
Evaluation of the effectiveness of A‒FeO_1‐x_OH in the treatment of tumors. a) Schematic diagram of 4T1‒tumor‒bearing mouse model establishment and the tumor treatment by A‒FeO_1‐x_OH. b) Relative growth curves of 4T1 tumors treated with different groups within 16 days. c) Weight changes of 4T1 tumors treated with different groups for 16 days (*n* = 7, *p < 0.05, **p < 0.01 and ***p < 0.001). The results are reported as means standard deviation. d) Digital photo of 4T1 tumors after receiving different treatments. e) H&E, GPX4, Ki67, Tunnel, DHE images of tumor tissues after different treatments.

In order to assess the therapeutic efficacy of A‒FeO_1‐x_OH on tumors, the Balb/c mice were injected with 4T1 cells 7 days before the treatment to establish animal models of tumors. When the tumor volume reached ≈100 mm^3^, these tumor‒bearing mice were randomly divided into control, allicin, FeO_1‐x_OH and A‒FeO_1‐x_OH groups. Then, the nanomedicines were injected via the tail vein on day 0, 4, 8, and 12. The body weight were daily recorded during the 16‒day treatment, showing no significant abnormal change during the treatment (**Figure** **8**a).

In terms of tumor volume, FeO_1‐x_OH group exhibits a low growth rate during the observation period, much lower than that of the control and allicin groups, suggesting a mild inhibitory effect on tumor growth. Owing to the loading of allicin, A‒FeO_1‐x_OH group shows a significant decrease of tumor volume during the treatments especially in the later stage, demonstrating an excellent antitumor effect (Figure 8b; Figures S37 and S38, Supporting Information). In the 16th day, the plasma, serum, heart, liver, spleen, lung, kidney and tumor samples were collected for pathological observation. The H&E images demonstrate no abnormality after the treatment, ensuring the biosafety of A‒FeO_1‐x_OH (Figure S39, Supporting Information). Most attractively, tumors on three mice from total five mice have been completely eliminated, while the average tumor weights of FeO_1‐x_OH and allicin group are 313.6 and 799.3 mg, respectively (Figure 8c,d). The research results verify the highly promising treatment efficacy of allicin‒loaded A‒FeO_1‐x_OH nanomedicine for tumor therapy.

The H&E staining of tumor tissues results confirm that A‒FeO_1‐x_OH treatment can cause a violent damage to tumor tissues, followed by FeO_1‐x_OH, while allicin is the weakest (Figure 8e). The GPX4‒staining results exhibit prevailing staining areas of A‒FeO_1‐x_OH and FeO_1‐x_OH, indicating that these nanomedicines have induced strong ferroptosis on tumor cells and tissues. The images of the Ki67‒stained sections show no Ki67‒stained areas in the allicin and A‒FeO_1‐x_OH groups. On the contrary, large Ki67‒stained area can be found in the control group (Figure 8e).

Owing to the GSH‒oxidation‒promoted Fenton reaction and the loading of allicin, A‒FeO_1‐x_OH displays a highly attractive tumor‒inhibiting effect, featuring marked ferroptosis and proliferation inhibition on cancer cells. To further evaluate apoptosis in tumor tissues, sections of tumor tissues were stained using the TdT‒mediated dUTP Nick‒End Labeling (TUNEL) method. The images indicate that A‒FeO_1‐x_OH group shows the most serious cell apoptosis, followed by FeO_1‐x_OH and allicin groups, in comparison with control group (Figure 8e). The dihydroethidium bromide (DHE)‒stained section images confirm that FeO_1‐x_OH and A‒FeO_1‐x_OH groups produce much more ROS than other groups, which is consistent with previous results, that is, FeO_1‐x_OH catalyzes Fenton reaction of over‒expressed H_2_O_2_ in tumors (Figure 8e). Furthermore, all untreated tumor‒bearing mice in the control group died within 16 days, while all mice in A‒FeO_1‐x_OH group survived for over 39 days, compared with that of FeO_1‐x_OH (36 days) and allicin (24 days) groups, confirming the substantially enhanced therapeutic effectiveness of this antitumor nanomedicine (Figure S40, Supporting Information).

The ICD effect triggered by ROS production through A‒FeO_1‐x_OH in tumor tissues was also explored in‒depth. Tumor tissues were processed into single‒cell suspension to quantify DC maturation, and flow cytometry was used to detect the CD11c^+^, CD80^+^, CD86^+^, DC populations in each group. From the experimental results, it can be found that, compared with the control group, the number of DC cells in tissues treated with A‒FeO_1‐x_OH has significantly increased. The CD80^+^ and CD86^+^ levels in the FeO_1‐x_OH group are similar to those in the A‒FeO_1‐x_OH group, while those of the allicin group is close to that of the control group (Figure S41, Supporting Information). These results are consistent with the previous WB results of endoplasmic reticulum stress, indicating that ROS generated by A‒FeO_1‐x_OH can promote the maturation of DC cells through endoplasmic reticulum stress. Subsequently, the DCs‒mediated immune response was further investigated by studying the infiltration of T lymphocytes in tumors, and the results are similar to the previous findings. After treatment with A‒FeO_1‐x_OH, increased CD3^+^ and CD8^+^ levels can be observed (Figure S42, Supporting Information). Therefore, these results collectively indicate that A‒FeO_1‐x_OH can induce the production of ROS, further triggering the ICD to achieve tumor immunotherapy. Moreover, the inhibitory effect of A‒FeO_1‐x_OH on tumor recurrence was investigated by following experiments. The tumor‒bearing mice were injected with allicin, FeO_1‐x_OH, A‒FeO_1‐x_OH after surgical resection of half of the tumor on the 15th day post tumor implantation (Figure S43a and b, Supporting Information).

The tumor volume of mice in the allicin group remained unchanged during the treatment period compared to the control group after surgical treatment, which indicates the allicin can inhibit the growth of tumor cells effectively. In contrast, the tumor volume of mice in the FeO_1‐x_OH group continues to increase, possibly due to the larger initial tumor volume on the 15th day, suggesting the poor tumor recurrence inhibitory effect by FeO_1‐x_OH alone. The tumor volume of mice in the A‒FeO_1‐x_OH group is significantly reduced, indicating the tumor cell proliferation has been largely inhibited by the loaded allicin and Fenton reaction taking place within tumor tissue (Figures S43c,d, and S44, Supporting Information). These comprehensive animal experiments illustrate the excellent tumor therapeutic and recurrence inhibitory efficacies of this A‒FeO_1‐x_OH nanomedicine, which is achieved by nanocatalytic Fenton reaction and oxidative GSH depletion by FeO_1‐x_OH and the loaded allicin.

## 3 Conclusion

In summary, this study has developed a promising but facile allicin‒loaded FeO_1‐x_OH nanomedicine, which presents cooperative effects between allicin and Fe(II, III) cycling for the effective and sustained tumor therapy. The coexistent Fe(II) and Fe(III) in FeO_1‐x_OH not only oxidizes GSH, but also catalyzes the Fenton reaction in an efficient and sustained manner through GSH oxidation‒promoted Fe(III) reduction to Fe(II), resulting in abundant ROS production without the deterioration by the high‒expressed GSH in tumor cells. Moreover, allicin decorated on the FeO_1‐x_OH surface plays a highly significant role in inhibiting the recurrence of tumor cells. The present nanocatalytic therapeutic strategy combines tumor regression and recurrence inhibition in a collaborative manner, effectively overcoming the problems encountered in traditional nanocatalytic drugs. This study provides an example for developing nanomedicines that achieve both effective tumor treatment and recurrence prevention, demonstrating promising prospect for future clinic translation.

## Conflict of Interest

The authors declare no conflict of interest.

## Supporting information

Supporting Information

## Data Availability

The data that support the findings of this study are available from the corresponding author upon reasonable request.
