# Supplementary material for: Allicin‒Decorated FeO1‐xOH Nanocatalytic Medicine for Fe2+/Fe3+ Cycling‒Promoted Efficient and Sustained Tumor Regression
Source: Adv Sci (Weinh). 2024 Jun 20;11(32):2402801. doi: 10.1002/advs.202402801 (PMC11348051; doi:10.1002/advs.202402801)
Supplement: Supplementary file 1 — Supporting Information [file ADVS-11-2402801-s001.docx]

# Supporting Information

# Allicin-Decorated FeO_1-x_OH Nanocatalytic Medicine for Fe^2+^/Fe^3+^ Cycling-Promoted Efficient and Sustained Tumor Regression

*Zhongming Jie, Bingyan Xiong,* and Jianlin Shi*^,^*

J. Shi

Shanghai Tenth People’s Hospital, Shanghai Frontiers Science Center of Nanocatalytic Medicine, School of Medicine, Tongji University Shanghai, 200072, P. R. China

School of Physical Science and Technology, ShanghaiTech University Shanghai, 201210, P. R. China

Shanghai Institute of Ceramics, Chinese Academy of Sciences, Research Unit of Nanocatalytic Medicine in Specific Therapy for Serious Disease, Chinese Academy of Medical Sciences (2021RU012) Shanghai, 200050, P. R. China

Center of Materials Science and Optoelectronics Engineering, University of Chinese Academy of Sciences Beijing, 100049, P. R. China

E-mail: jlshi@mail.sic.ac.cn

B. Xiong

Shanghai Tenth People’s Hospital, Shanghai Frontiers Science Center of Nanocatalytic Medicine, School of Medicine, Tongji University Shanghai, 200072, P. R. China

E‒mail: xiongbingyan720@126.com

Z. Jie

School of Physical Science and Technology, ShanghaiTech University Shanghai, 201210, P. R. China

**Supplementary Figures**


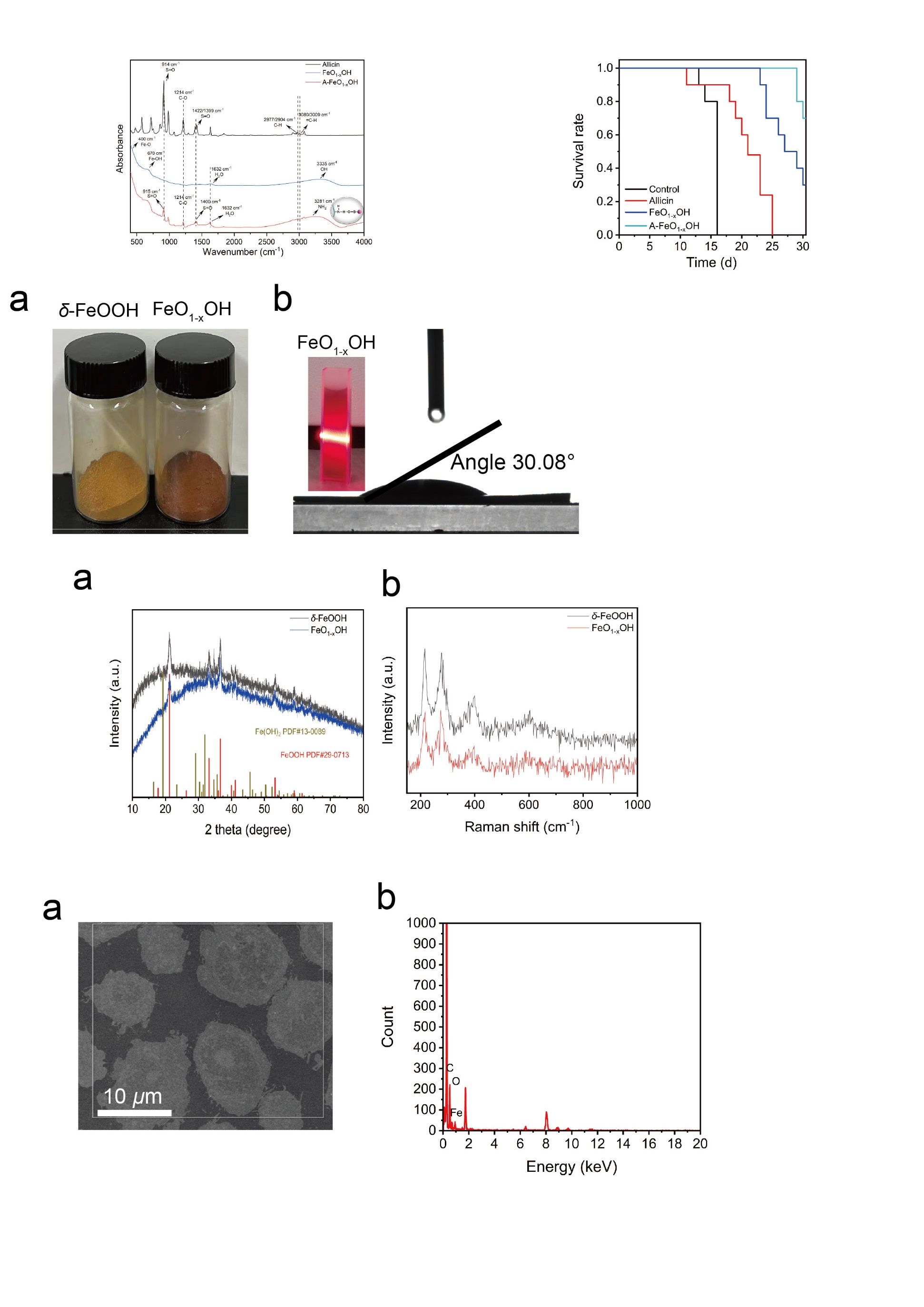


**Figure S1.** a) XRD patterns of *δ*‒FeOOH and FeO_1-x_OH. b) Raman spectra of *δ*‒FeOOH and FeO_1-x_OH.

The XRD patterns of *δ*‒FeOOH and FeO_1-x_OH show several diffraction peaks at 21°, 33°, 34°, 36°, proving that they show a similar FeOOH orthorhombic structure (PDF #29‒0713) instead of a Fe(OH)_2_ structure. The Raman spectra show typical Fe‒O (215 cm^-1^, 276 cm^-1^), Fe‒OH (399 cm^-1^) peaks, confirming that both samples exist Fe‒O and Fe‒OH bonds (Figure S1b).


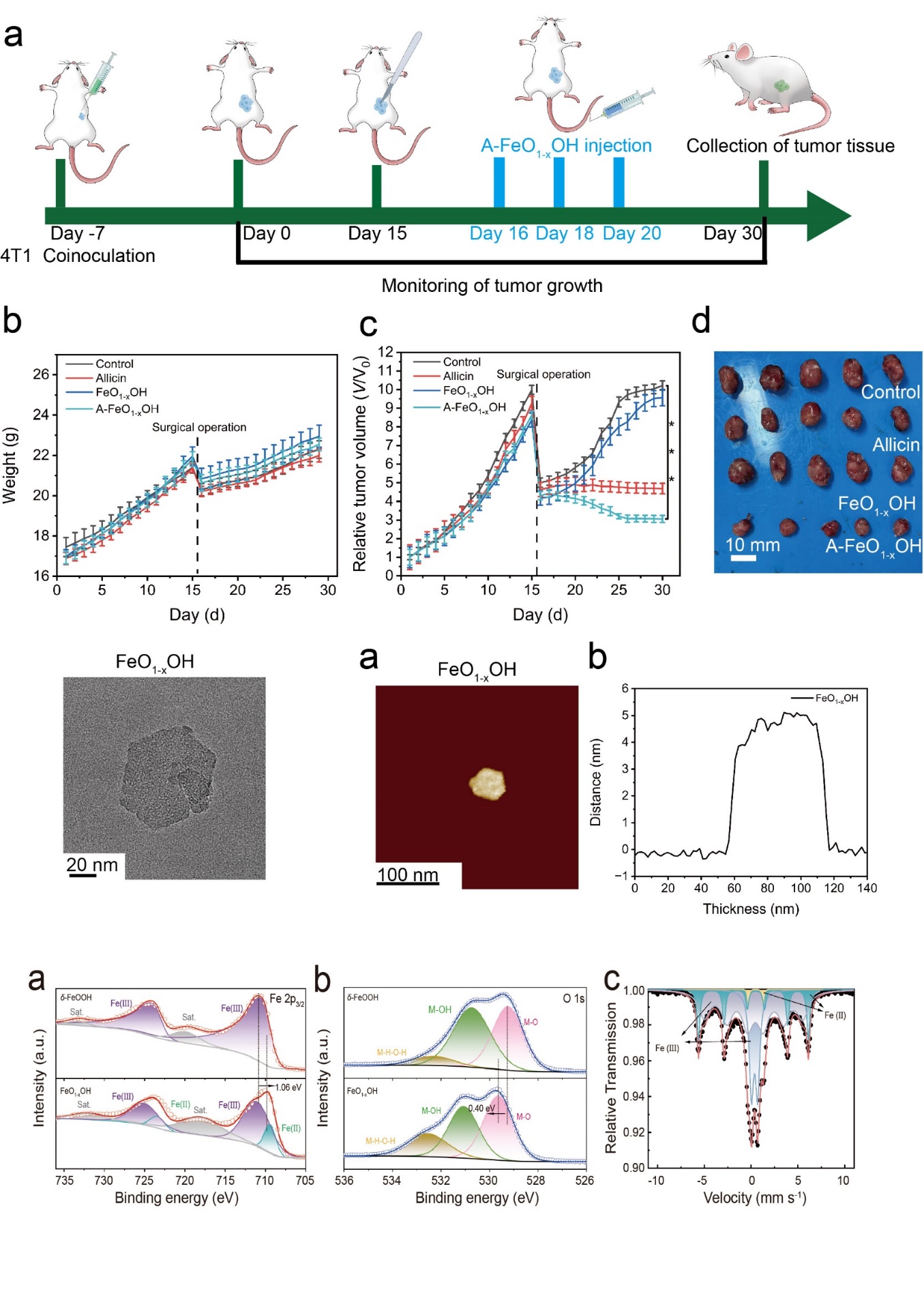


**Figure S2.** XPS spectra for a) Fe 2p_3/2_ of *δ*‒FeOOH and FeO_1-x_OH and b) O 1s of *δ*‒FeOOH and FeO_1-x_OH. c) Mossbauer spectrum of FeO_1-x_OH.

Two prominent peaks are observed at 709.48 eV and 725.18 eV, accompanied by two satellite peaks corresponding to Fe 2p_3/2_ and Fe 2p_1/2_, respectively. They can be deconvoluted into two sub‒peaks corresponding to Fe(II) and Fe(III).^[1]^ Additionally, the O 1s spectrum is deconvoluted into three sub‒peaks at 529.68 eV, 531.08 eV and 532.58 eV, belonging to Fe‒O, Fe‒OH bonds and Fe‒H_2_O, respectively.


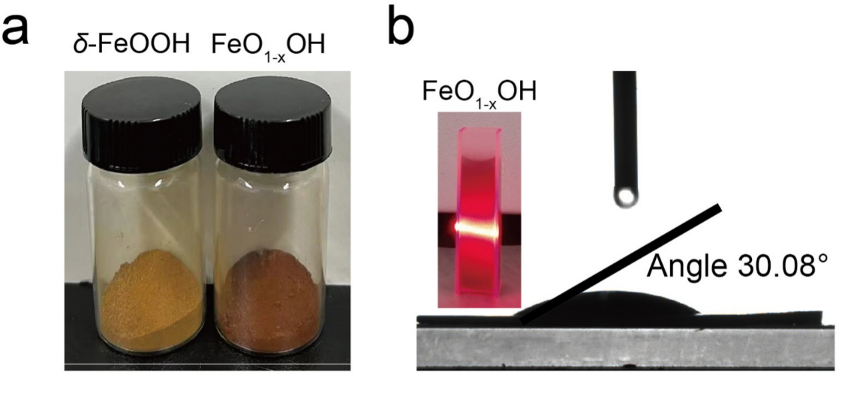


**Figure S3.** a) Digital photo of *δ*‒FeOOH and FeO_1-x_OH. b) The Tyndall effect is evident in the digital photograph of the FeO_1-x_OH nanocatalyst dispersed in deionized water for 12 h, and the contact angle test image of FeO_1-x_OH.


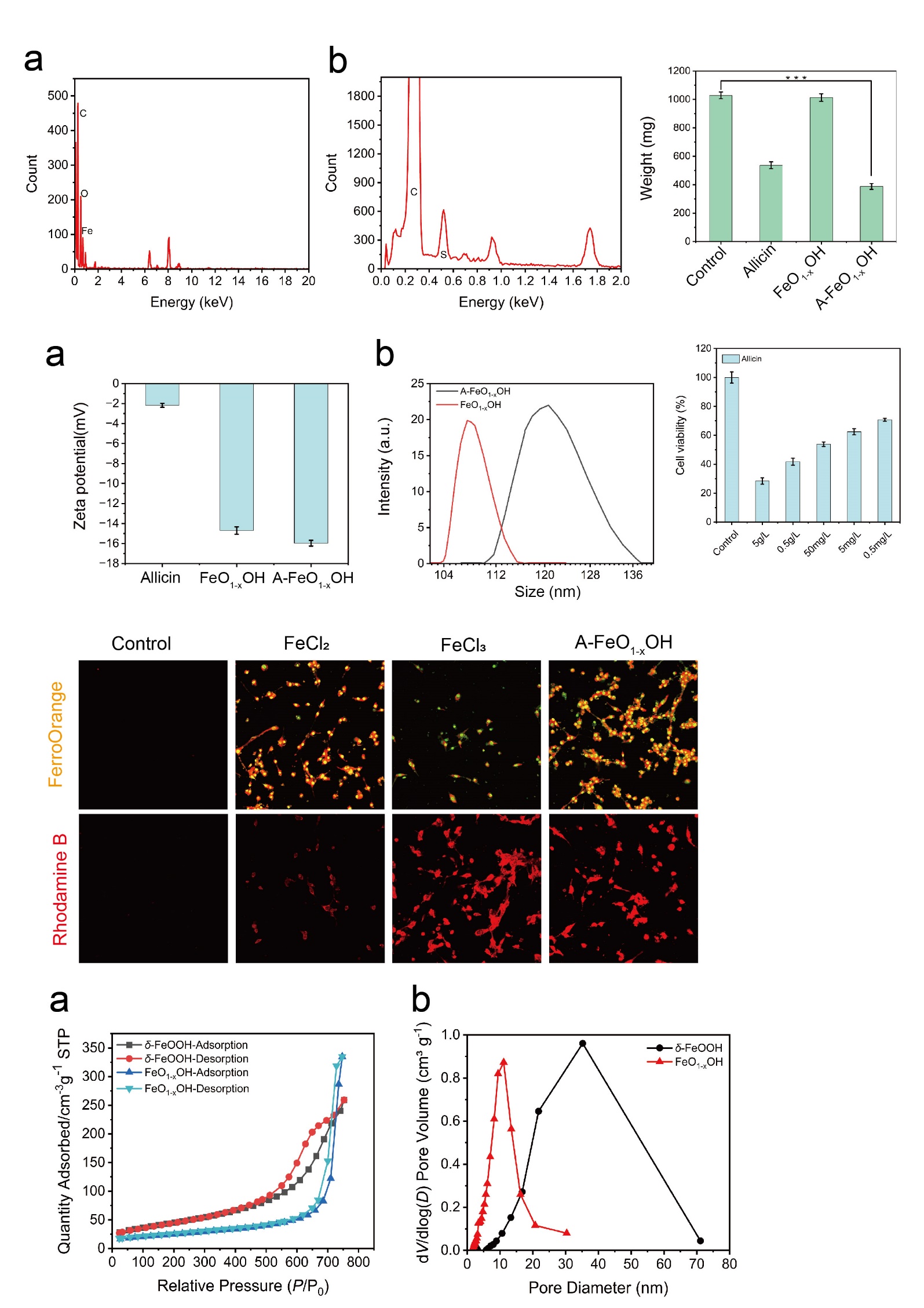


**Figure S4.** a) N_2_ adsorption‒desorption isotherms of *δ*‒FeOOH and PEGylation FeO_1-x_OH. b) Pore size distribution plots of *δ*‒FeOOH and PEGylation FeO_1-x_OH.


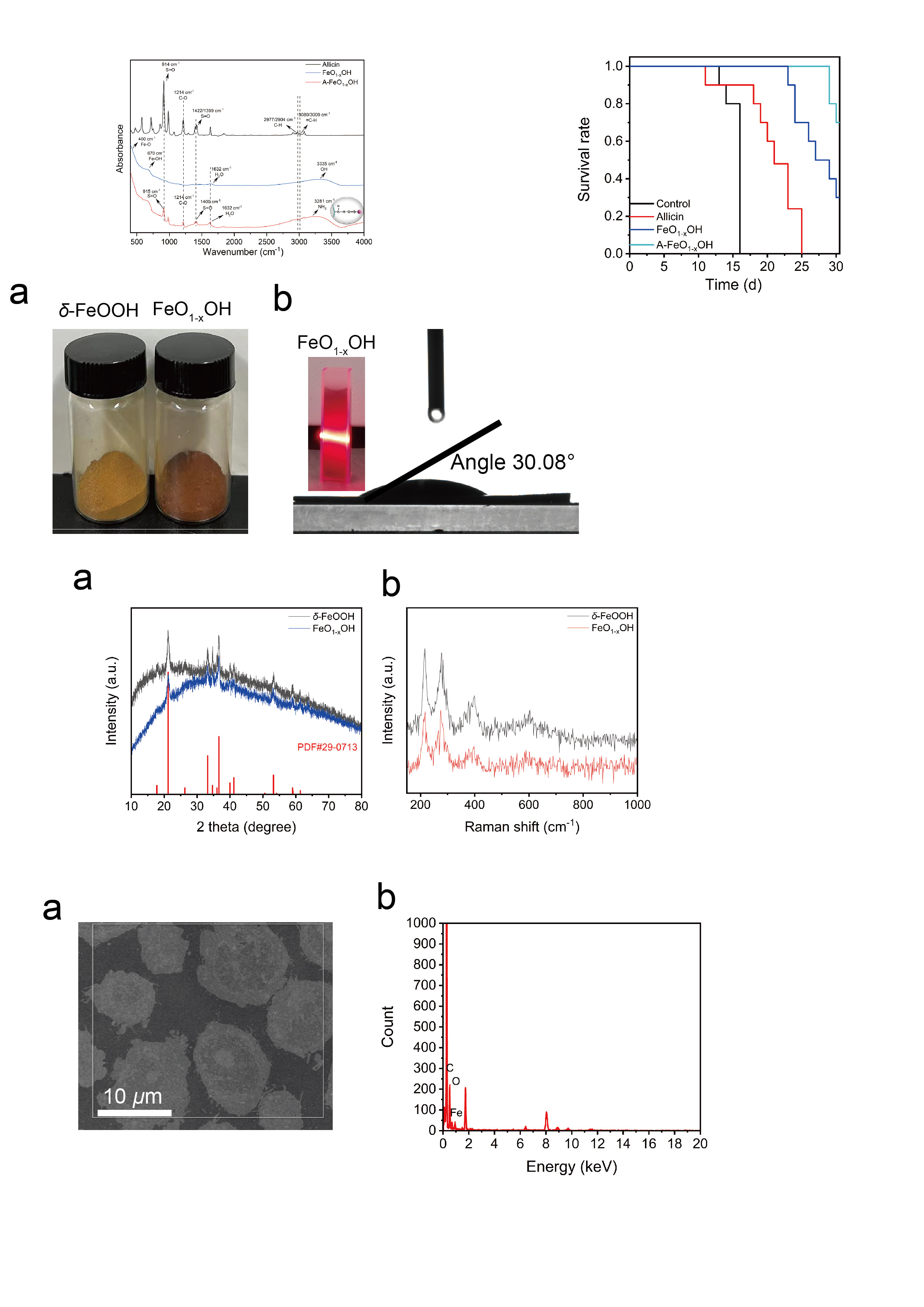


**Figure S5.** FT‒IR results of allicin, FeO_1-x_OH and A‒FeO_1-x_OH.

In detail, the absorption peaks at 3080 cm^-1^ and 3009 cm^-1^ mainly come from C‒H stretching vibrations of unsaturated olefins in allicin. The absorption peaks observed at 2977 cm^-1^ and 2904 cm^-1^ in A‒FeO_1-x_OH are associated with the symmetric and asymmetric stretching vibrations of the C‒H bonds in fatty acids. The absorption peaks at 1422 cm^-1^ and 1399 cm^-1^ are mainly from the S=O stretching vibrations. The absorption peak at 914 cm^-1^ is related to the S=O bond.^[2]^ Besides, FT‒IR spectrum obtained from FeO_1-x_OH demonstrates that the absorption peak at 3335 cm^-1^ derives from the stretching vibration of Fe‒OH and ‒OH groups in bound water. The weak peak shape at 1632 cm^-1^ confirms the presence of trace amounts of bound water in the sample. The absorption peak at 670 cm^-1^ is related to the stretching vibration of Fe‒OH bond. The most significant absorption peak at around 400 cm^-1^ in FT‒IR spectrum of A‒FeO_1-x_OH comes from the stretching vibration of Fe‒O in A‒FeO_1-x_OH.^[3]^ The spectroscopic characteristics of the A‒FeO_1-x_OH and FeO_1-x_OH samples are mostly similar, indicating their analogous chemical structures.


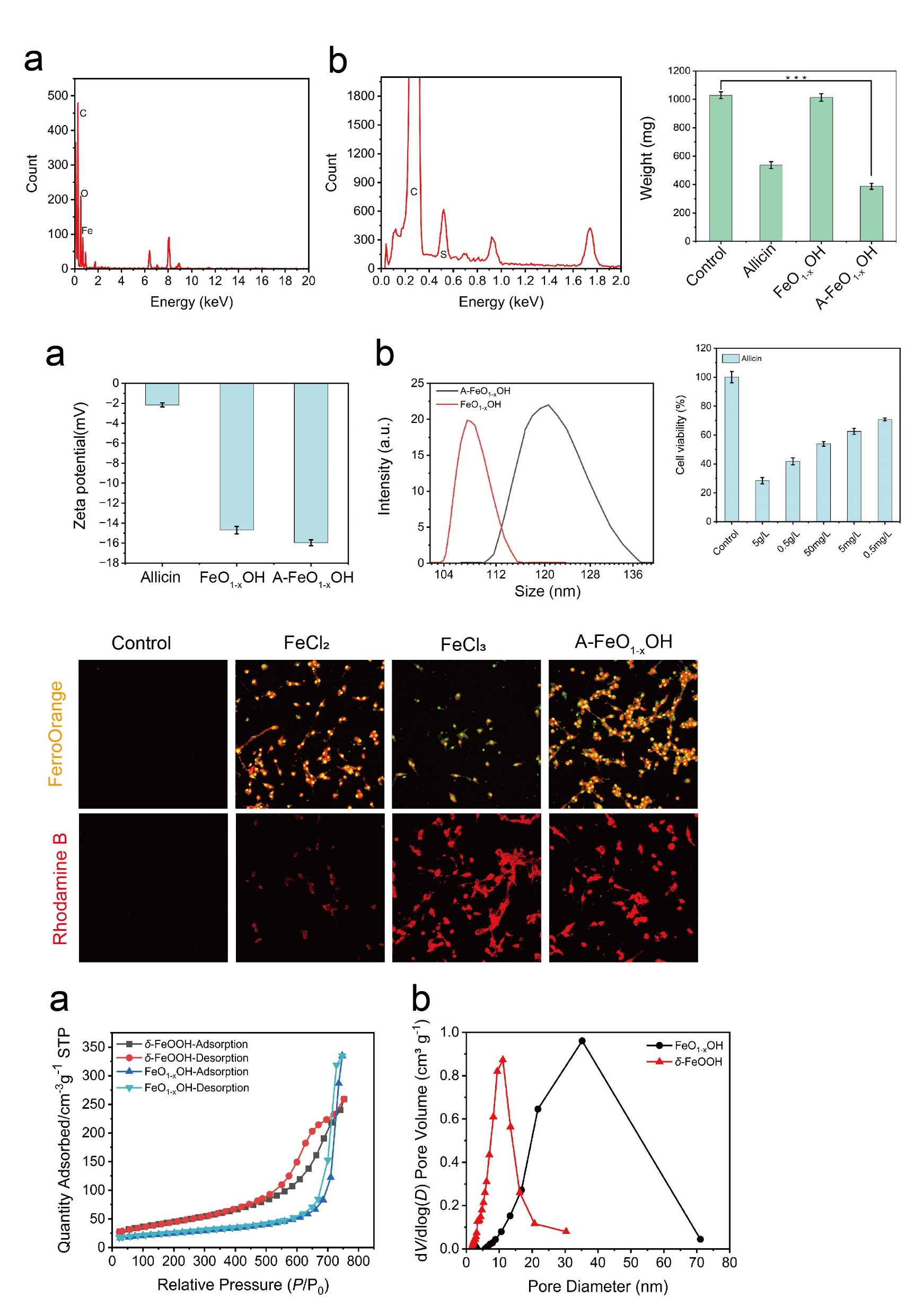


**Figure S6.** a) Zeta potential results of allicin, FeO_1-x_OH and A‒FeO_1-x_OH. The results are reported as means standard deviation (*n* = 3). b) Particle‒size distribution plots of FeO_1-x_OH and A‒FeO_1-x_OH.

According to the Zeta potential analysis, compared with FeO_1-x_OH, allicin‒loaded A‒FeO_1-x_OH exhibits a reduced potential, and the zeta potentials of allicin and FeO_1-x_OH are both negative, ruling out the influence of electrostatic attraction when connecting FeO_1-x_OH and allicin (Figure S6a). The particle size of A‒FeO_1-x_OH measured by DLS, can is larger than that of FeO_1-x_OH, indicating the successful loading of allicin (Figure S6b).


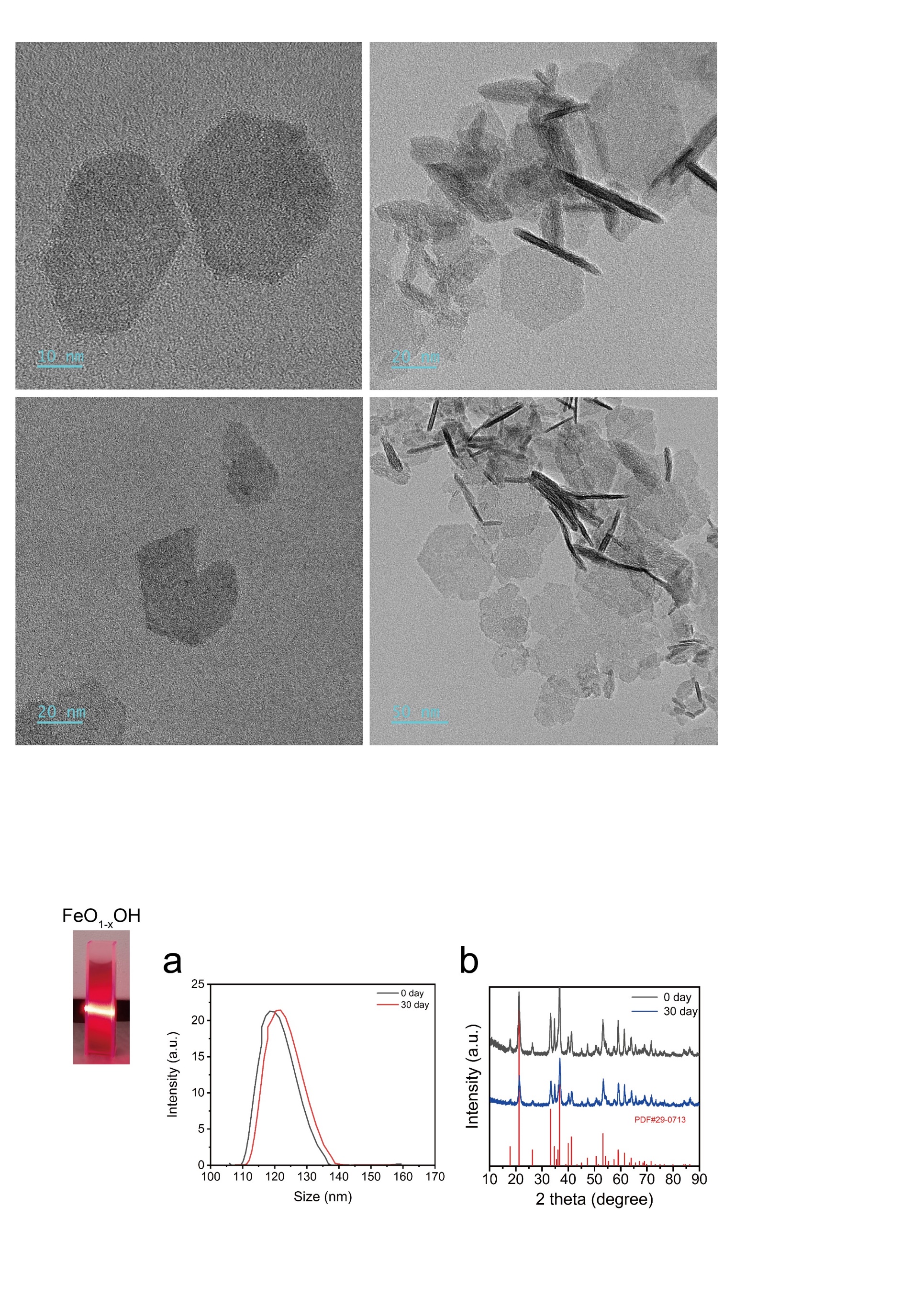


**Figure S7.** The XRD results and DLS particle size distribution data of A‒FeO_1-x_OH before and after 30 days.


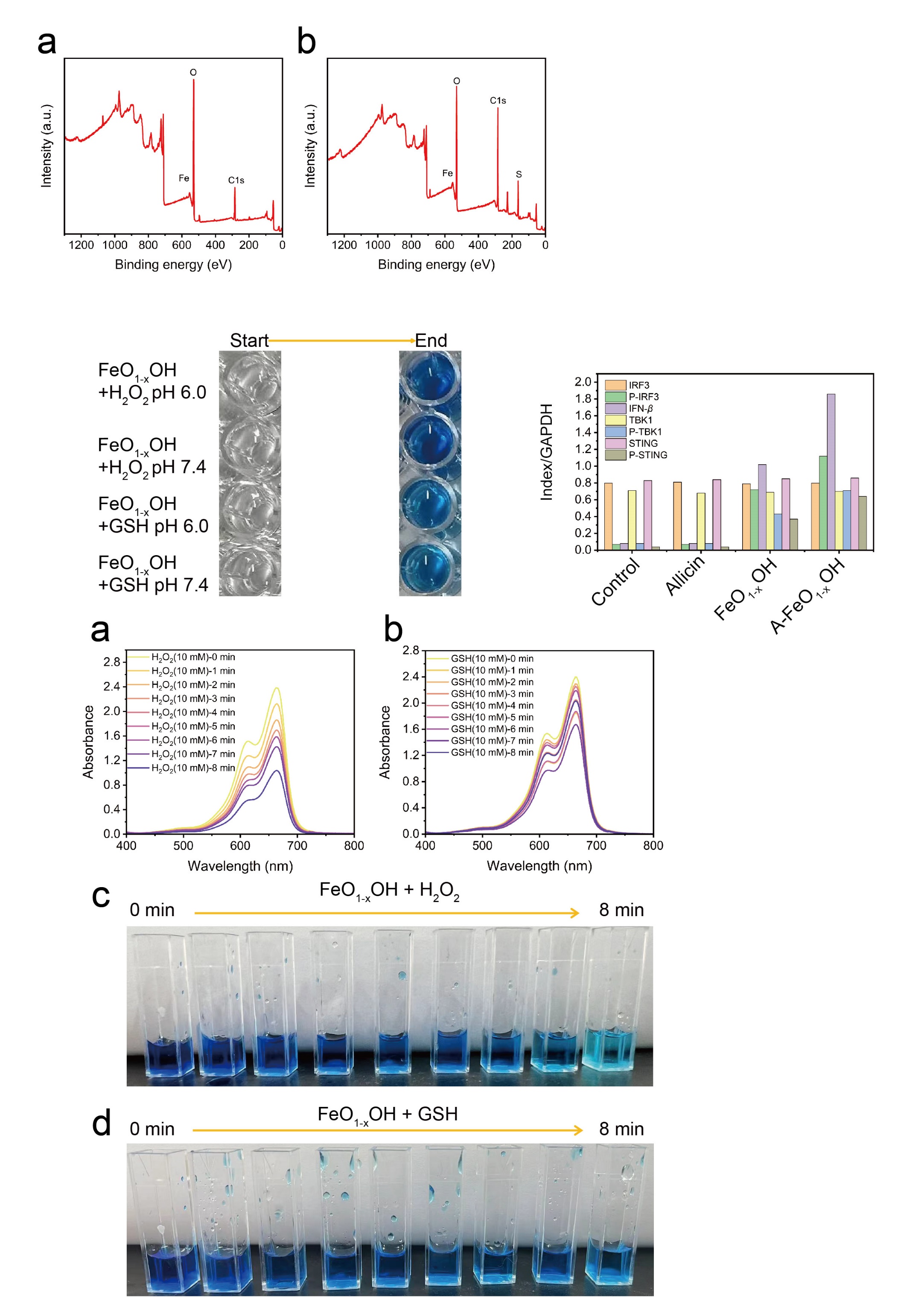


**Figure S8.** a, b) XPS survey spectra of (a) FeO_1-x_OH, (b) A‒FeO_1-x_OH.


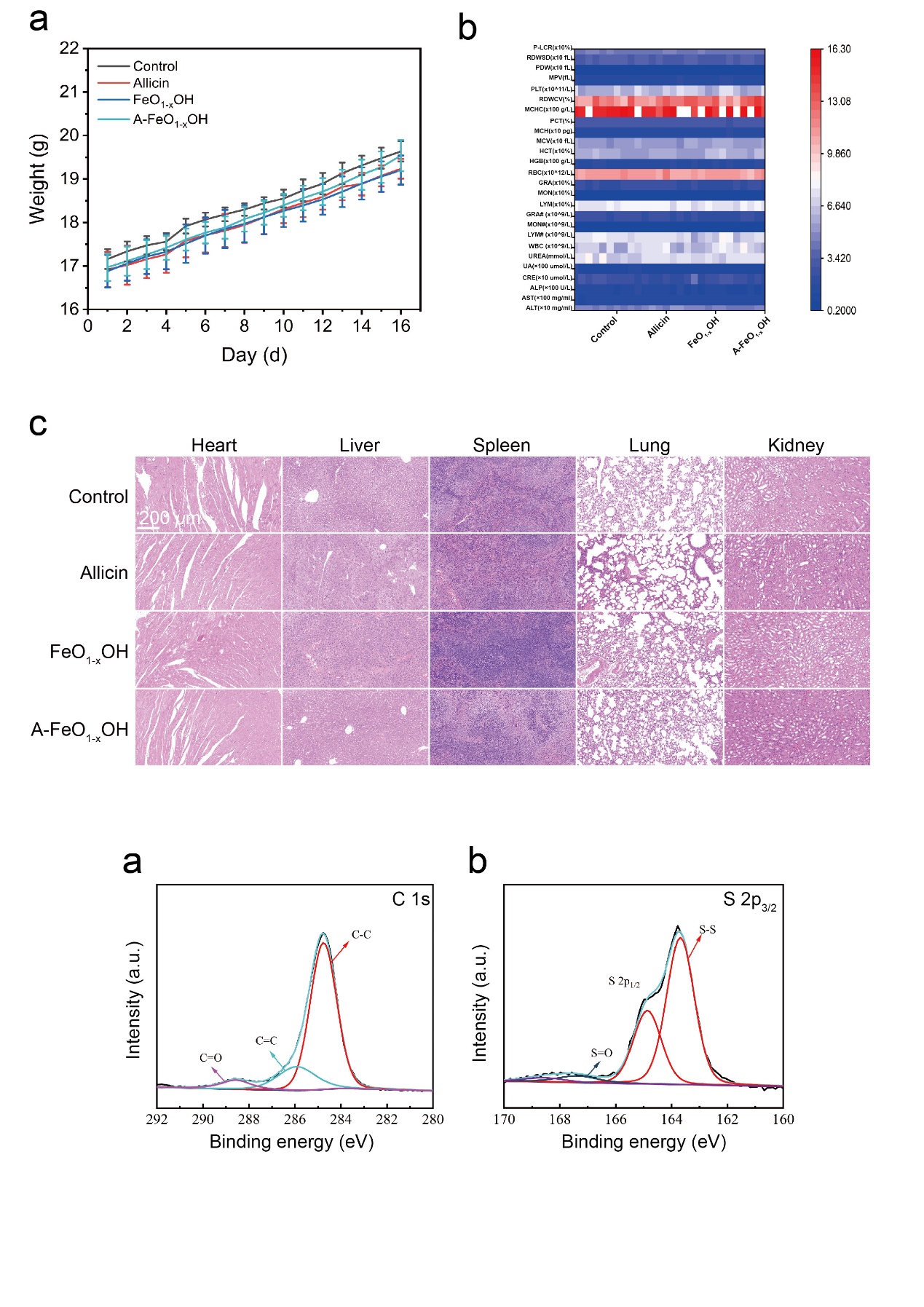


**Figure S9.** XPS spectra. a) C 1s and b) S 2p_3/2_ from A‒FeO_1-x_OH.

.


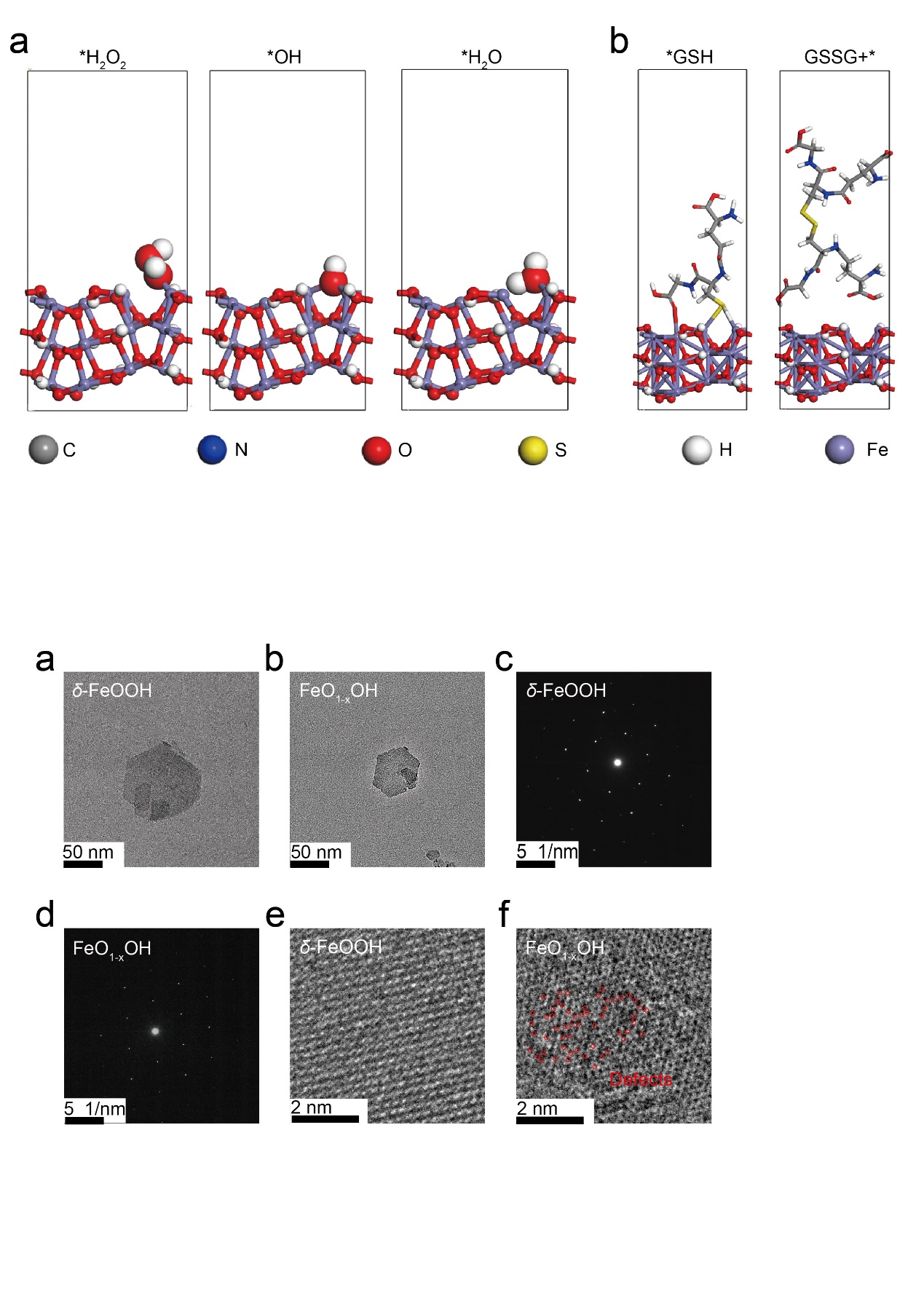


**Figure S10.** a, b) TEM images of *δ*‒FeOOH (a), FeO_1-x_OH (b). c, d) SAED pattern images of *δ*‒FeOOH (c), FeO_1-x_OH (d). e, f) AC‒TEM images for *δ*‒FeOOH (e), FeO_1-x_OH (f).


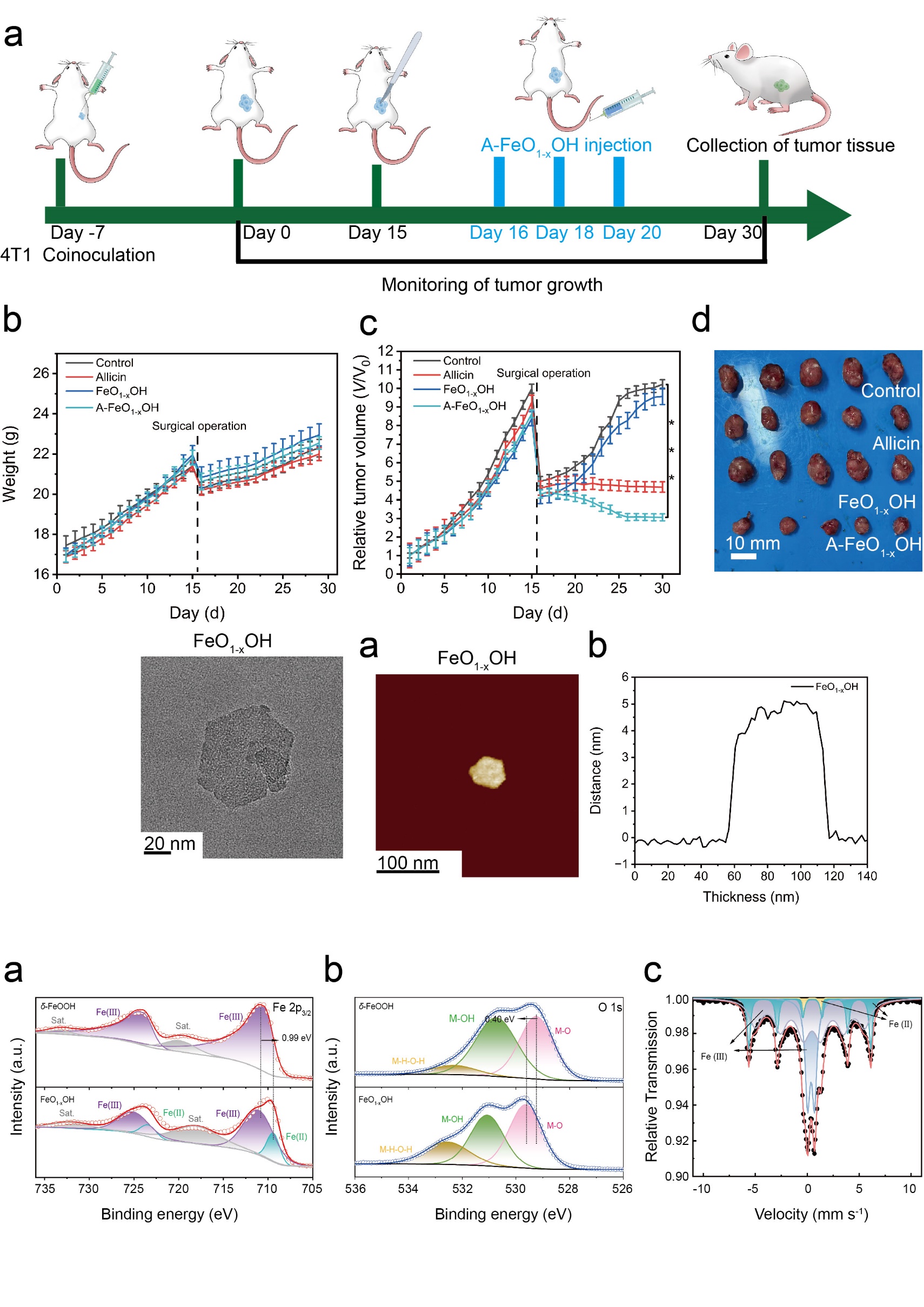


**Figure S11.** a) AFM image of FeO_1-x_OH. b) Thickness distribution data of FeO_1-x_OH.


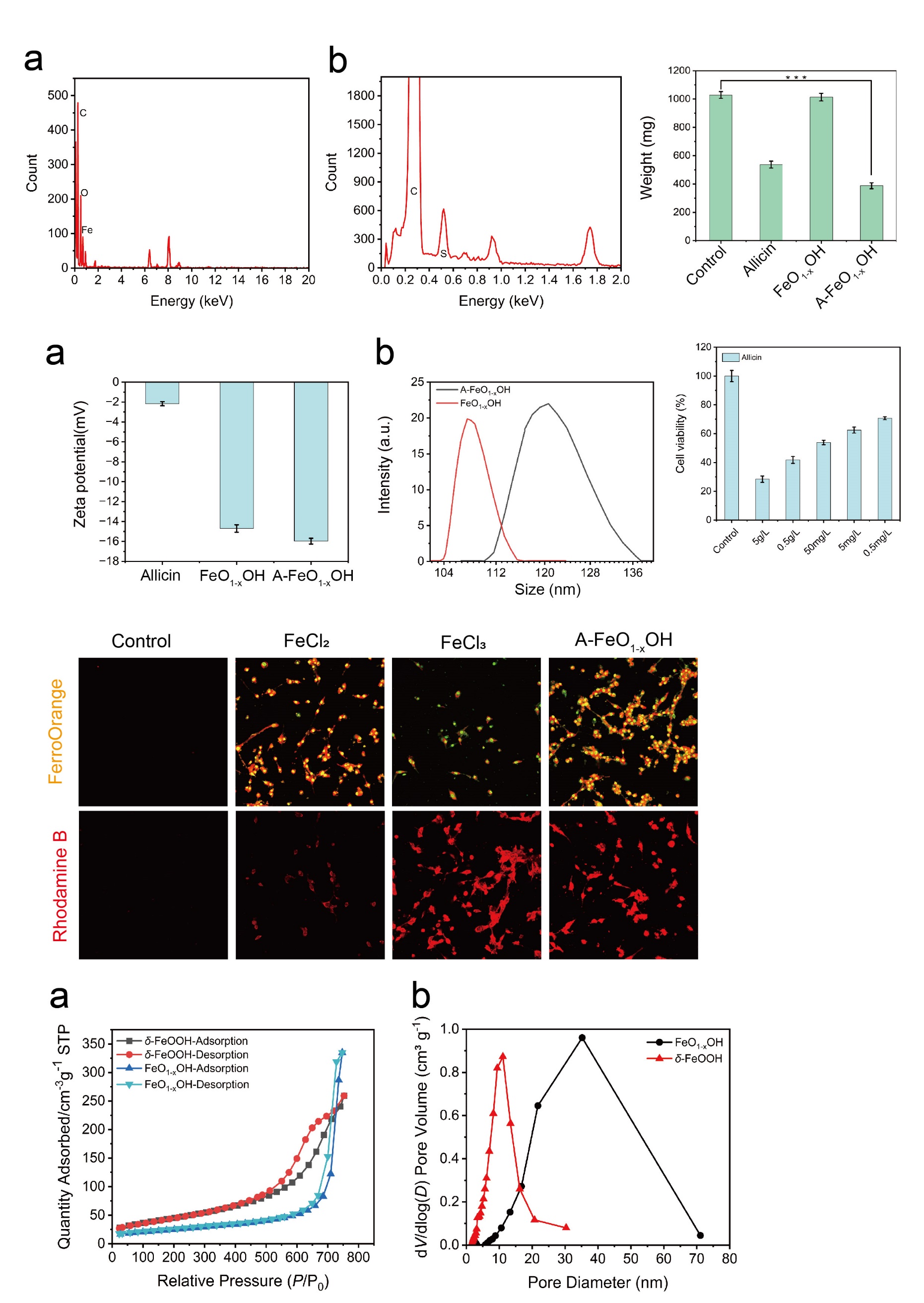


**Figure S12.** a, b) Element content statistical data from EDX spectra of FeO_1-x_OH (a) and A‒FeO_1-x_OH (b).


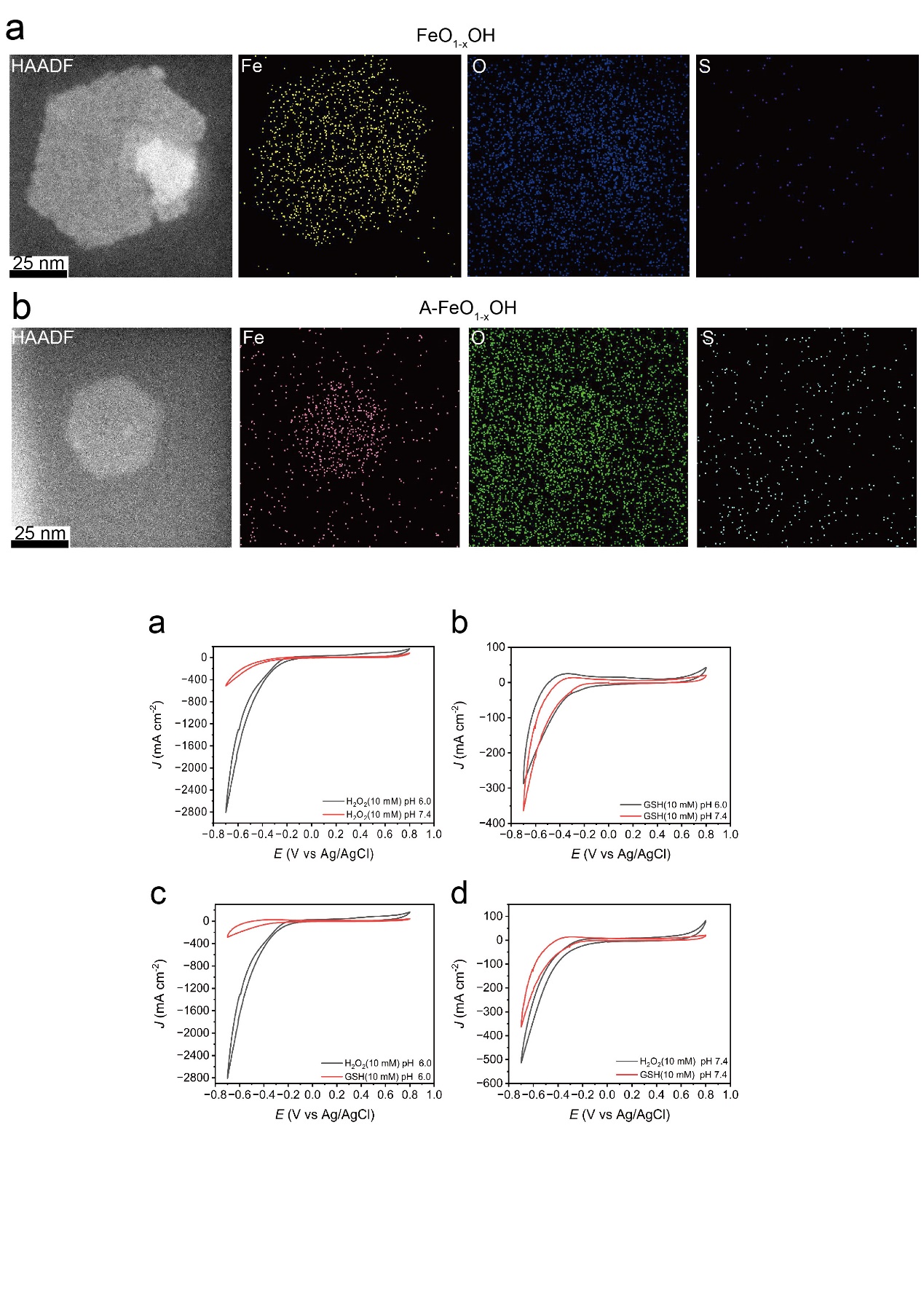


**Figure S13.** a, b) mapping images of Fe, O and S elements from FeO_1-x_OH (a) and A‒FeO_1-x_OH (b).


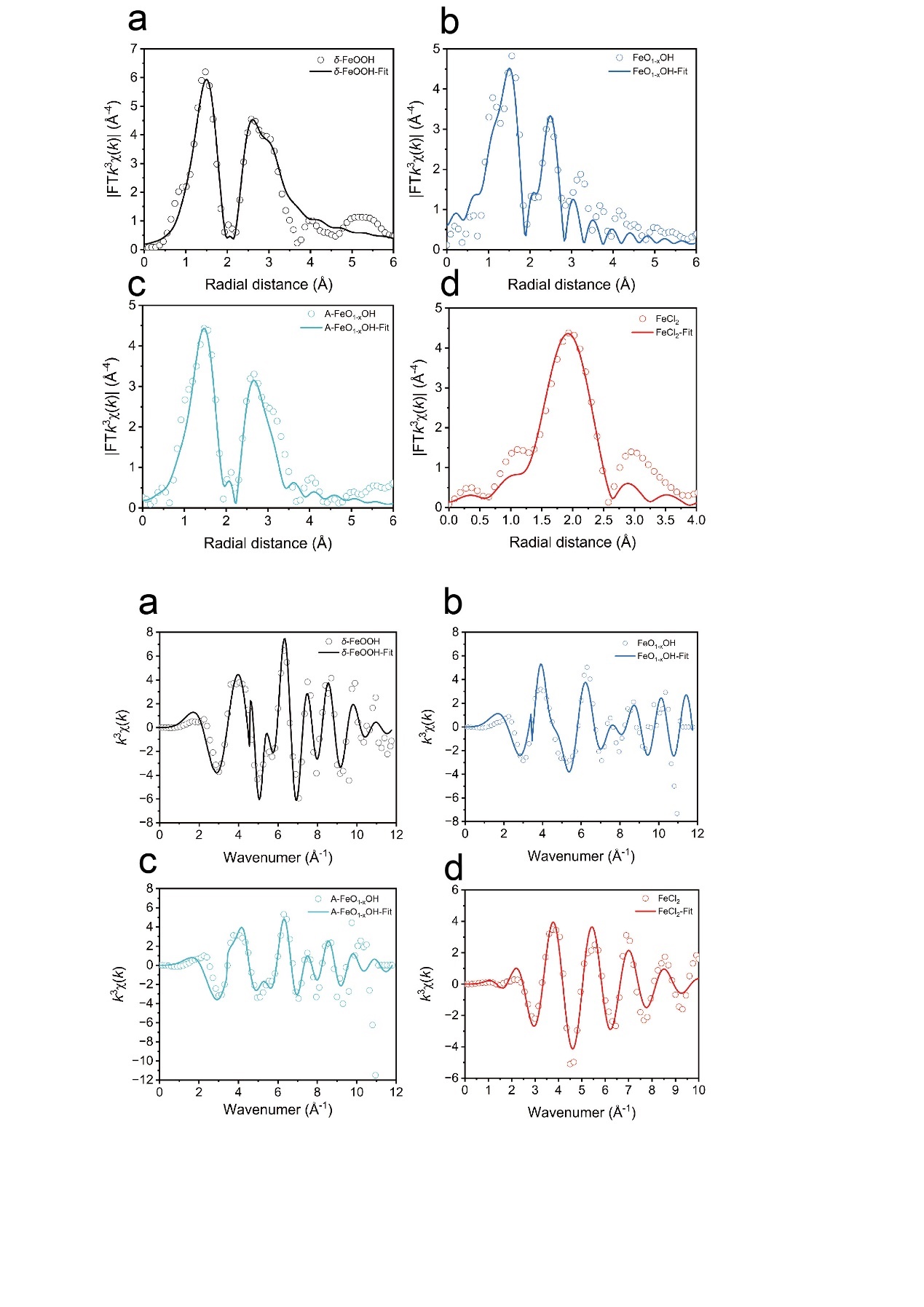


**Figure S14.** *K*‒space extended EXAFS spectra of a) *δ*‒FeOOH, b) FeO_1-x_OH, c) A‒FeO_1-x_OH and d) FeCl_2_.


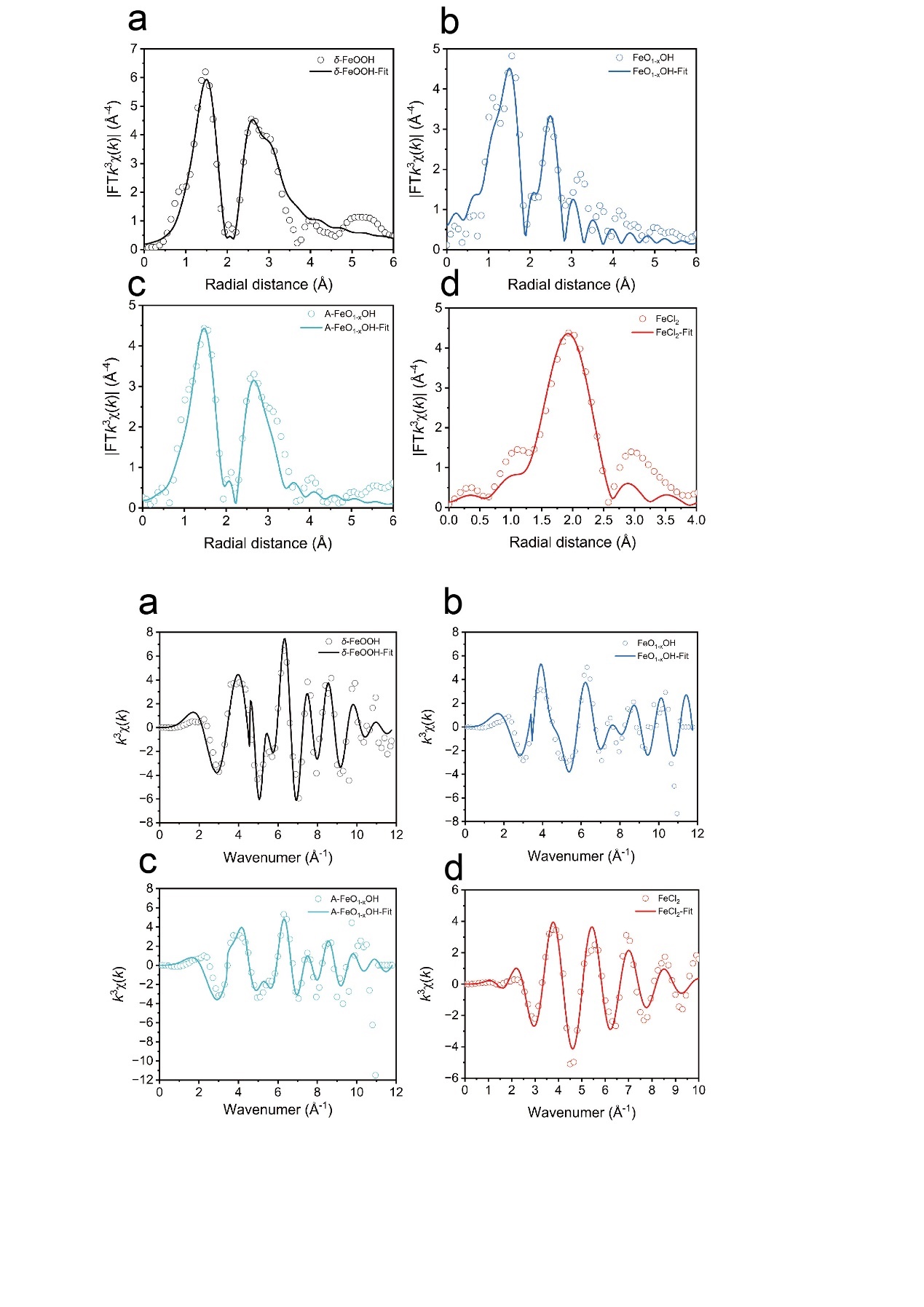


**Figure S15.** *R*‒space extended EXAFS spectra of a) *δ*‒FeOOH, b) FeO_1-x_OH, c) A‒FeO_1-x_OH and d) FeCl_2_.


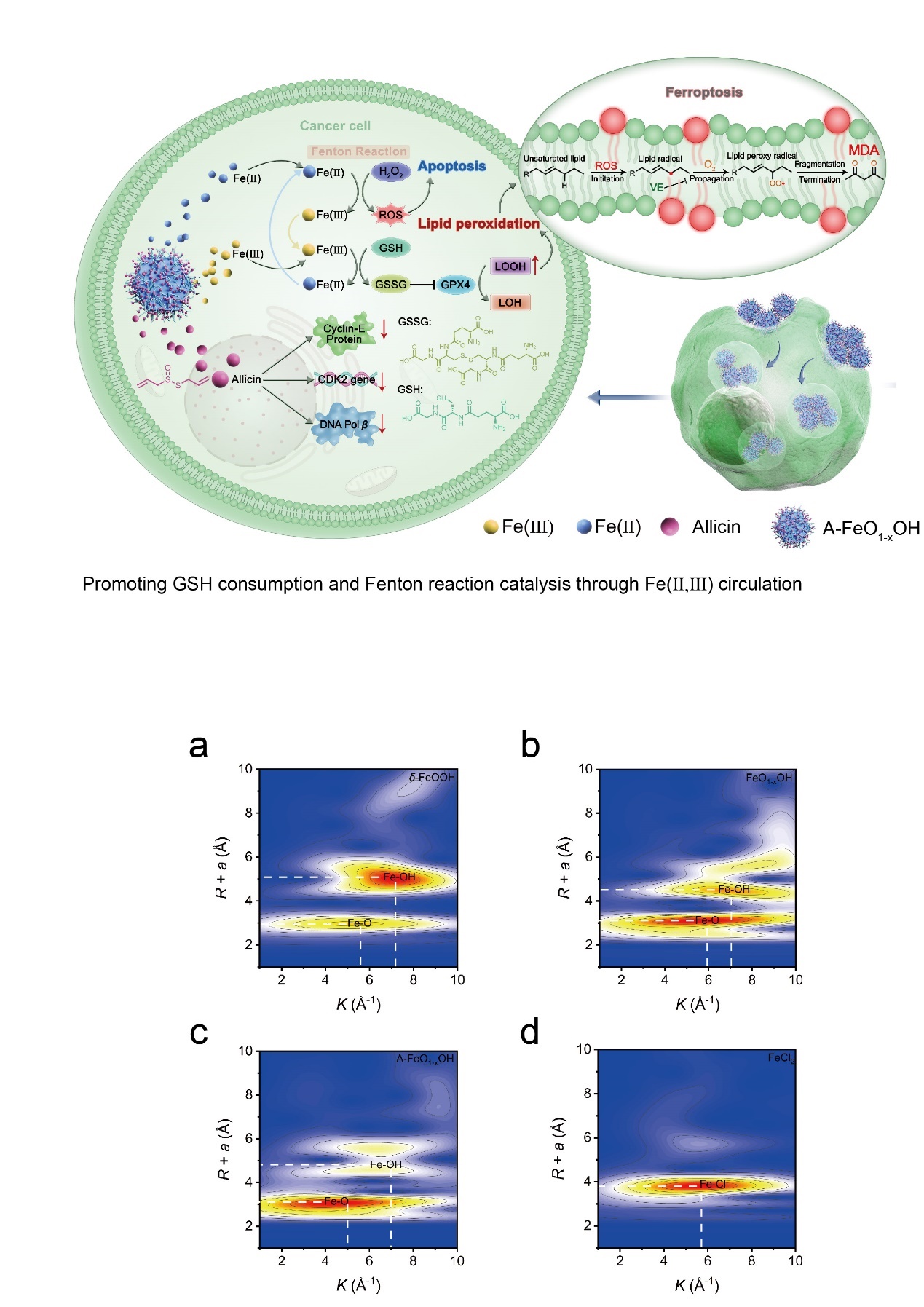


**Figure S16.** Wavelet transforms for the *k*^3^–weighted EXAFS signals of a) *δ*‒FeOOH, b) FeO_1-x_OH, c) A‒FeO_1-x_OH and d) FeCl_2_.


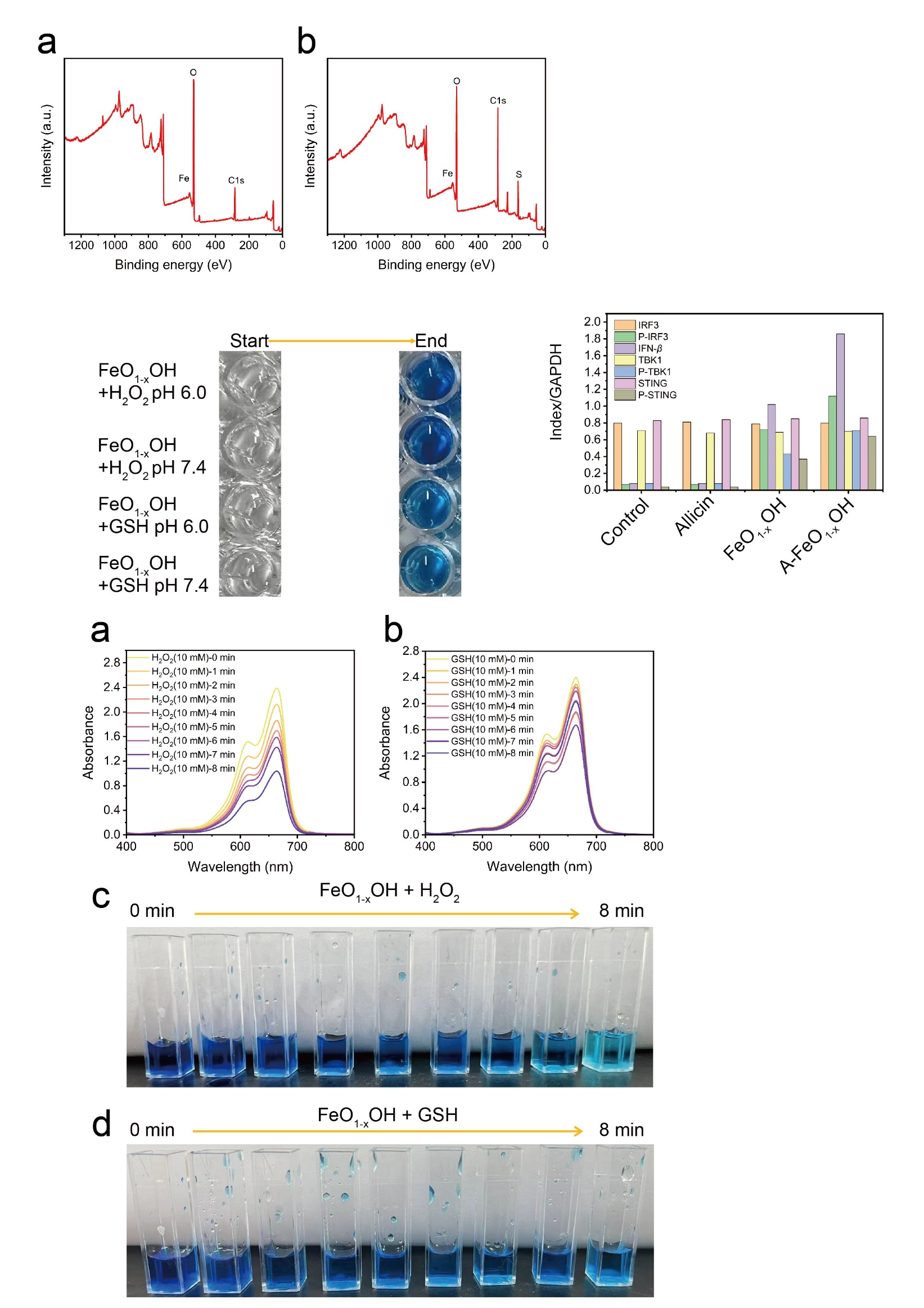


**Figure S17.** a, b) The absorbance results of FeO_1-x_OH catalyzes degradation of MB by H_2_O_2_ (a) and GSH (b) at different time points (pH 6.0). c, d) Digital photo of MB degradation by FeO_1-x_OH and H_2_O_2_ (c) and GSH (d).


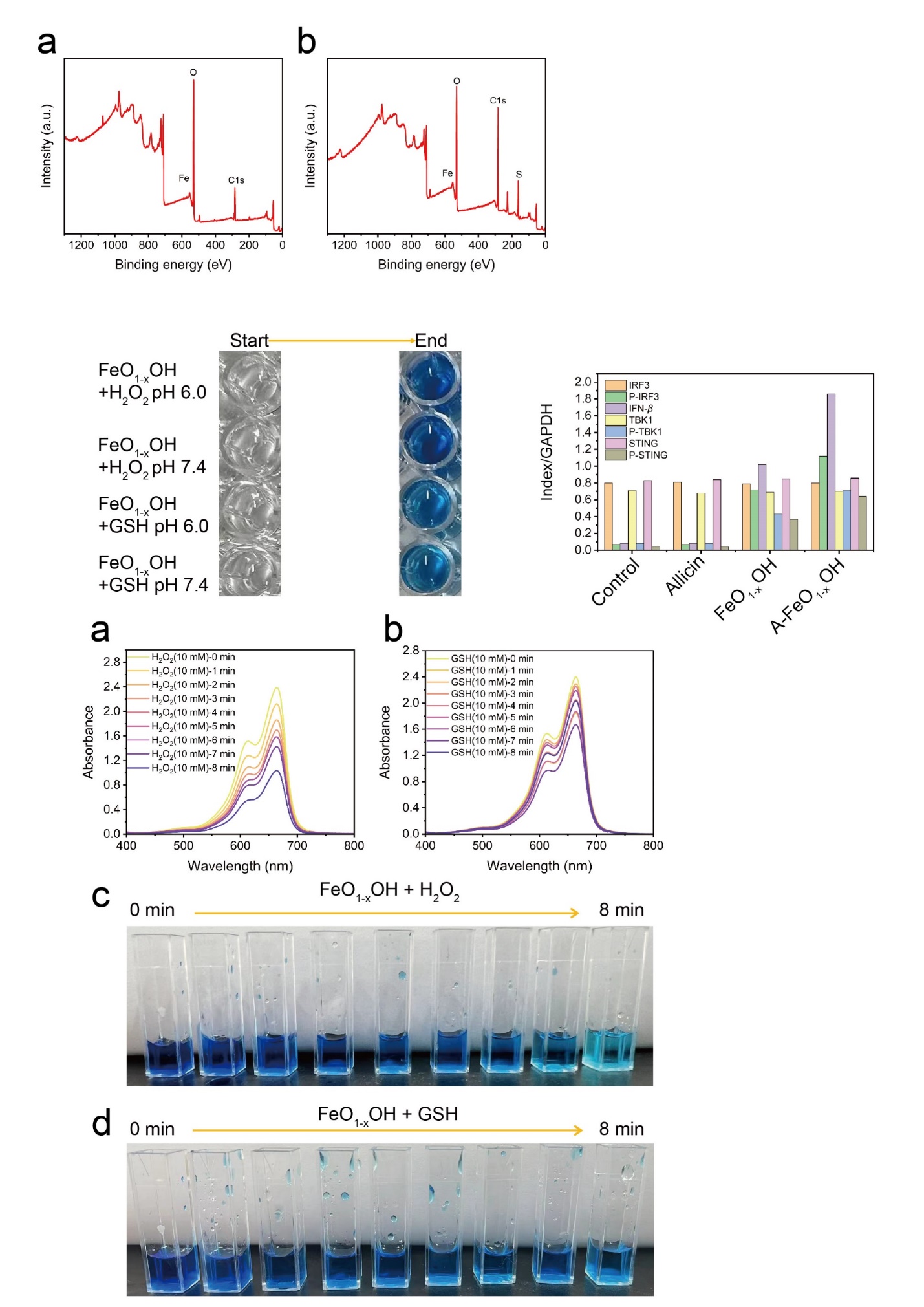


**Figure S18.** Digital photos of TMB color rendering by FeO_1-x_OH, H_2_O_2_ and GSH under different conditions.


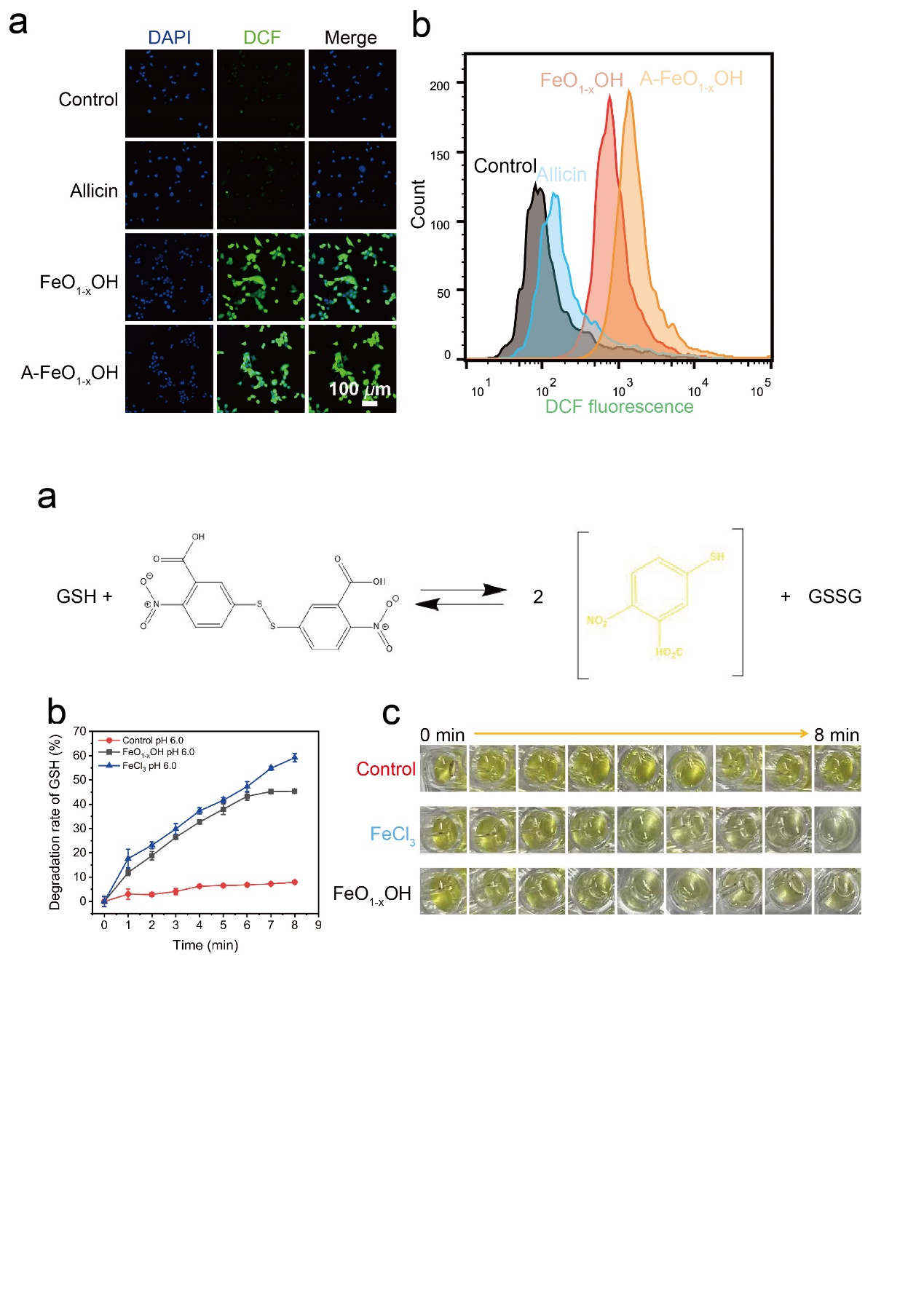


**Figure S19.** a) Molecular structure of DTNB and schematic diagram of colorimetric reaction principle between DTNB and GSH. b) UV‒Vis data of GSH oxidation by FeCl_3_ and FeO_1-x_OH determined by DTNB indicator. c) Digital photos of GSH oxidation by FeCl_3_ and FeO_1-x_OH determined by DTNB indicator.


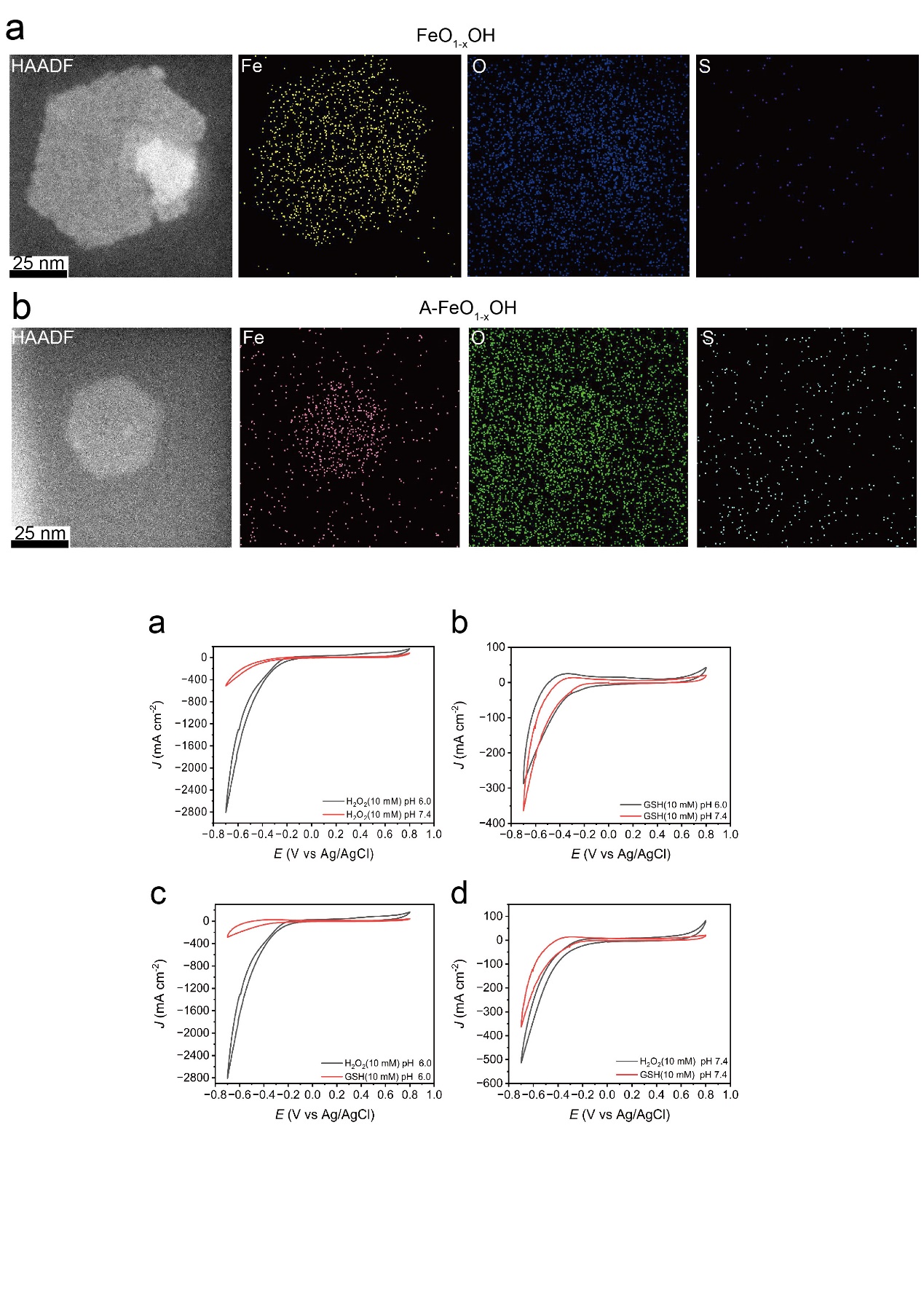


**Figure S20.** Cyclic voltammetry curves of FeO_1-x_OH catalyzes the Fenton reaction by H_2_O_2_ and GSH oxidation in different media.


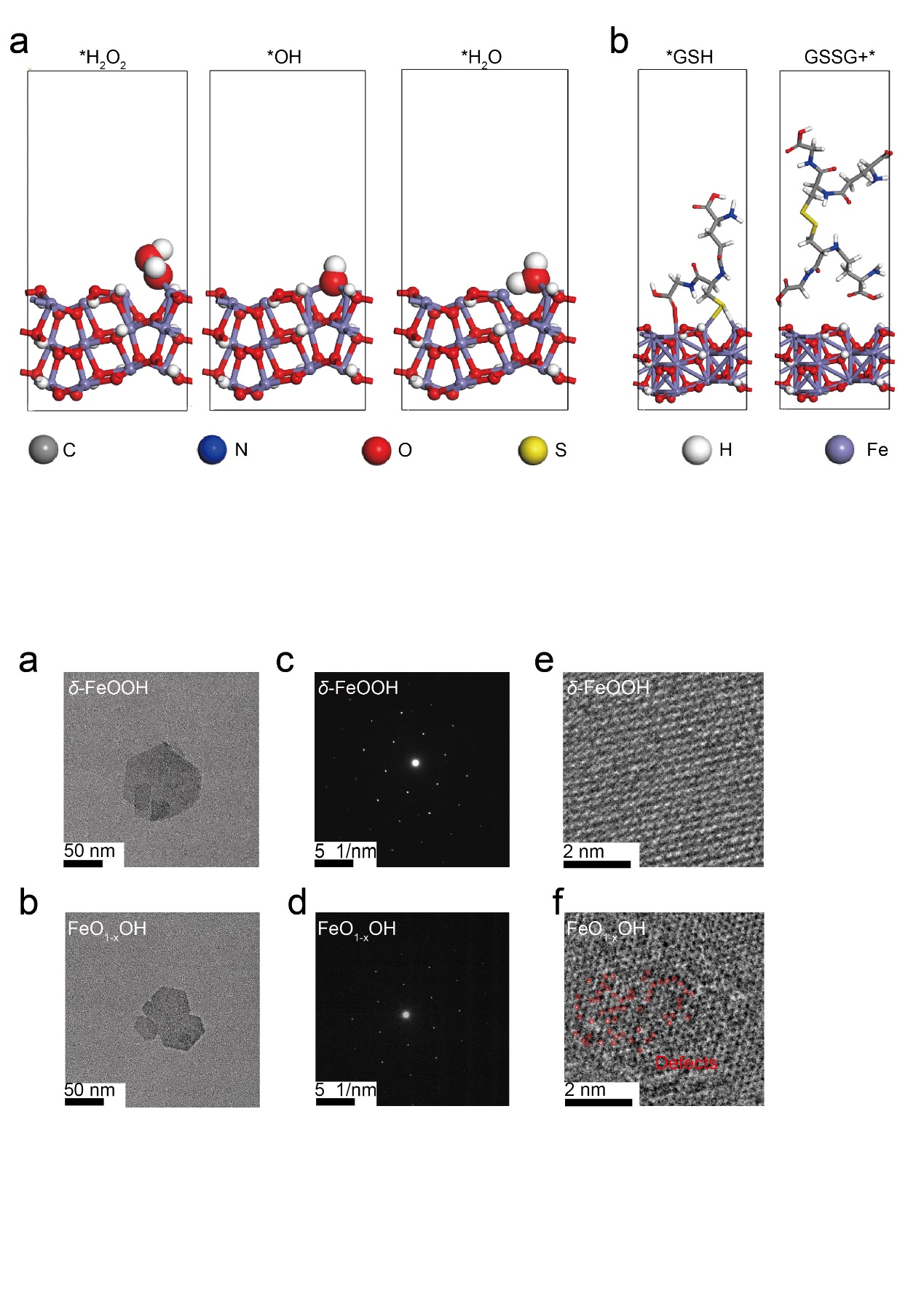


**Figure S21.** a) Molecular model diagram of *H_2_O_2_, *OH and *H_2_O at adsorption sites. b) Molecular model diagram of desorbed GSSG+* and *GSH at adsorption sites.


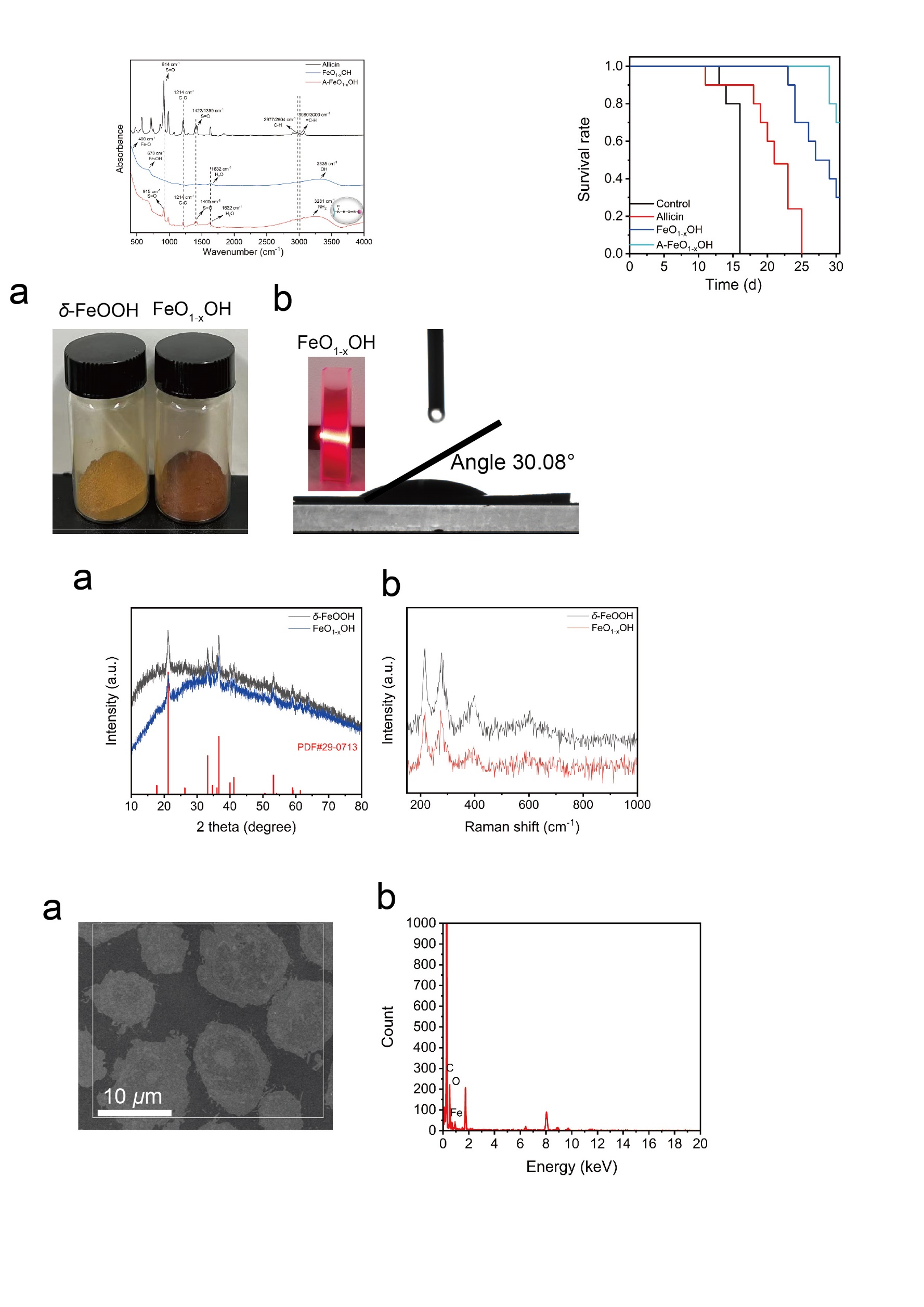


**Figure S22.** EDX a) image and b) spectrum of single 4T1 tumor cell co‒cultured with A‒FeO_1-x_OH for 3 h.


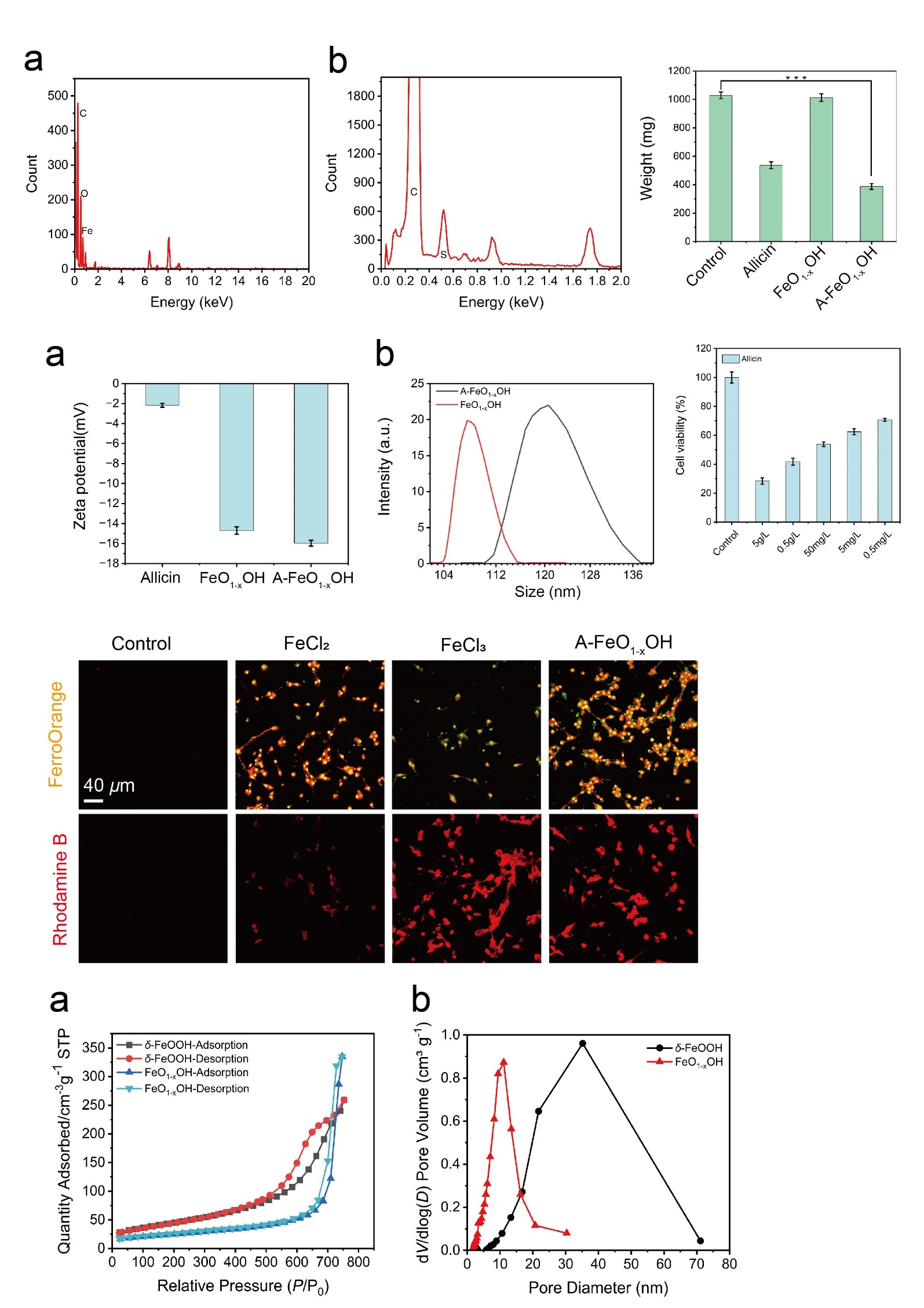


**Figure S23.** CLSM images of FerroOrange and Rhodamine B stained 4T1 tumor cells after co‒incubation with A‒FeO_1-x_OH, FeCl_2_, FeCl_3_ for 3 h.


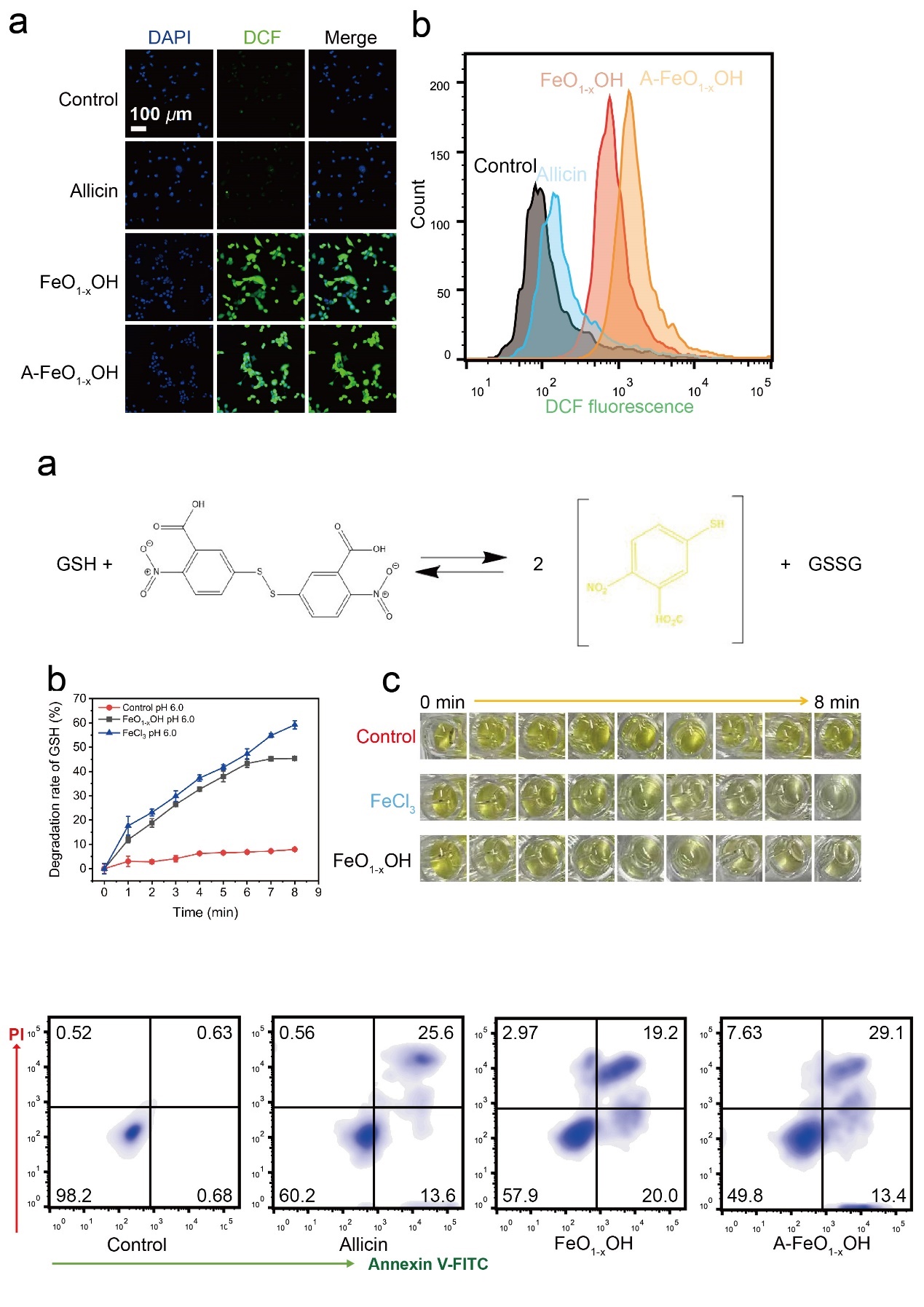


**Figure S24.** a) CLSM images of DAPI and DCFH‒DA stained 4T1 tumor cells after co‒incubation with allicin, FeO_1-x_OH and A‒FeO_1-x_OH for 3 h. b) Flow cytometry analysis of 4T1 tumor cells stained with DCFH‒DA after co-incubation with allicin, FeO_1-x_OH and A‒FeO_1-x_OH for 3 h.


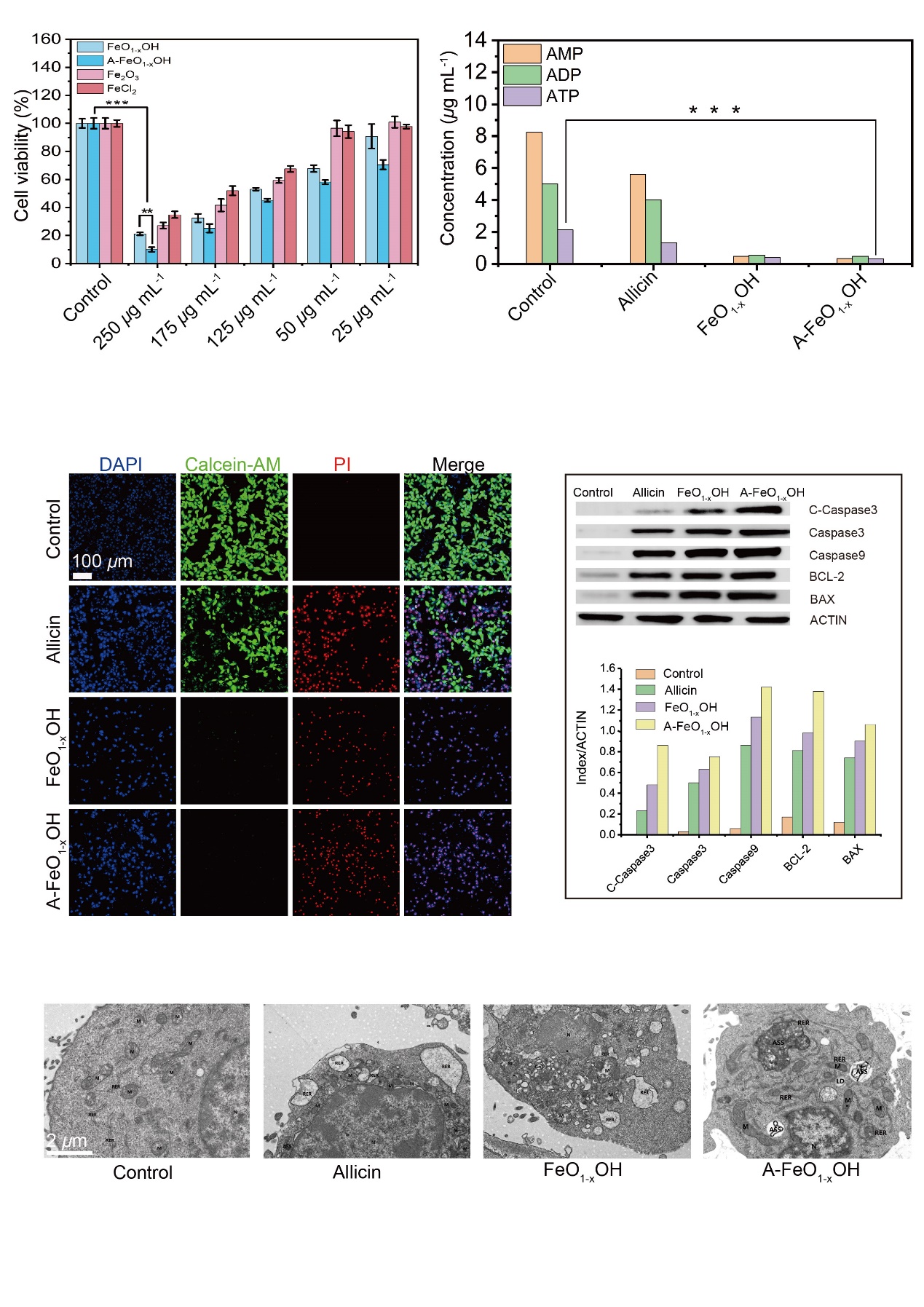


**Figure S25.** Cell viability of 4T1 cells treated with FeO_1-x_OH, Fe_2_O3, FeCl_2_ and A‒FeO_1-x_OH for 24 h. The results are reported as means standard deviation (*n* = 4). *p < 0.05, **p < 0.01, ***p < 0.001.


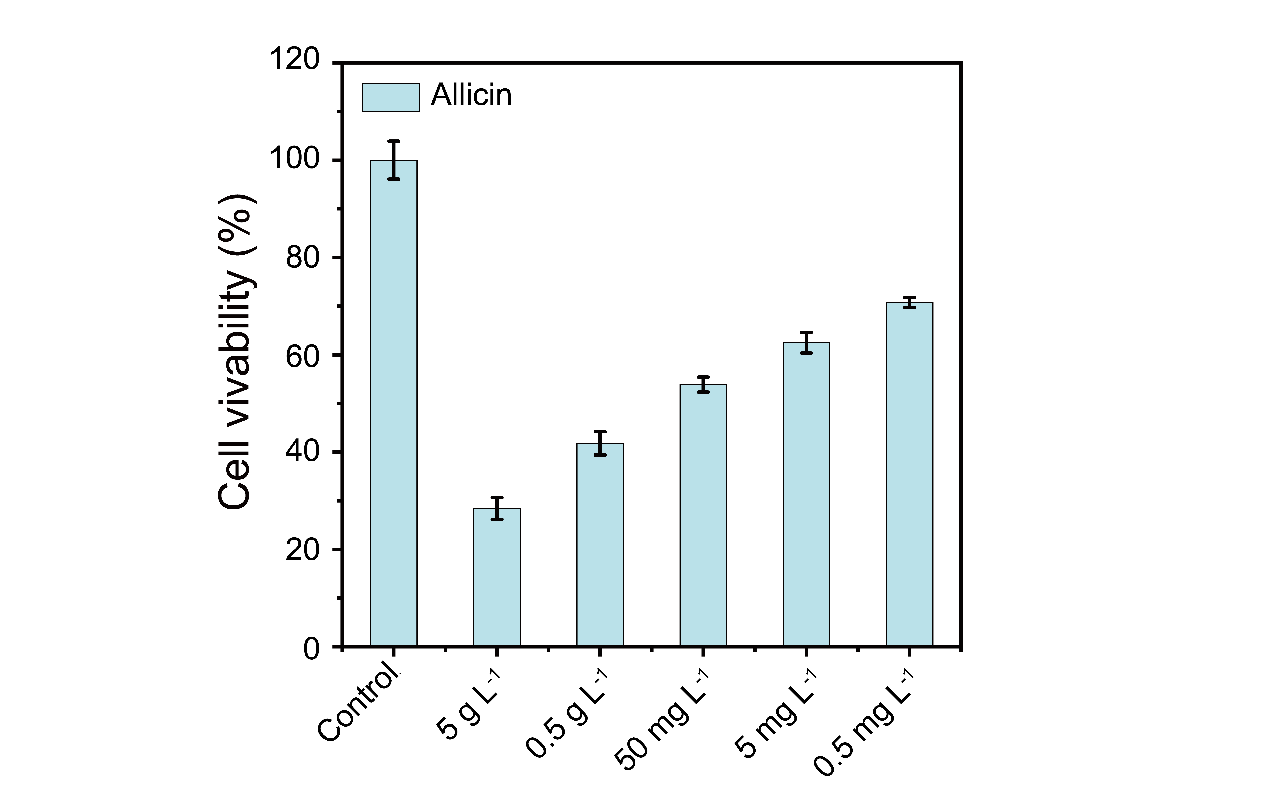


**Figure S26.** The viability of 4T1 cells is assessed after exposure to varying concentrations of allicin for a duration of 24 h. The results are reported as means standard deviation (*n* = 4).


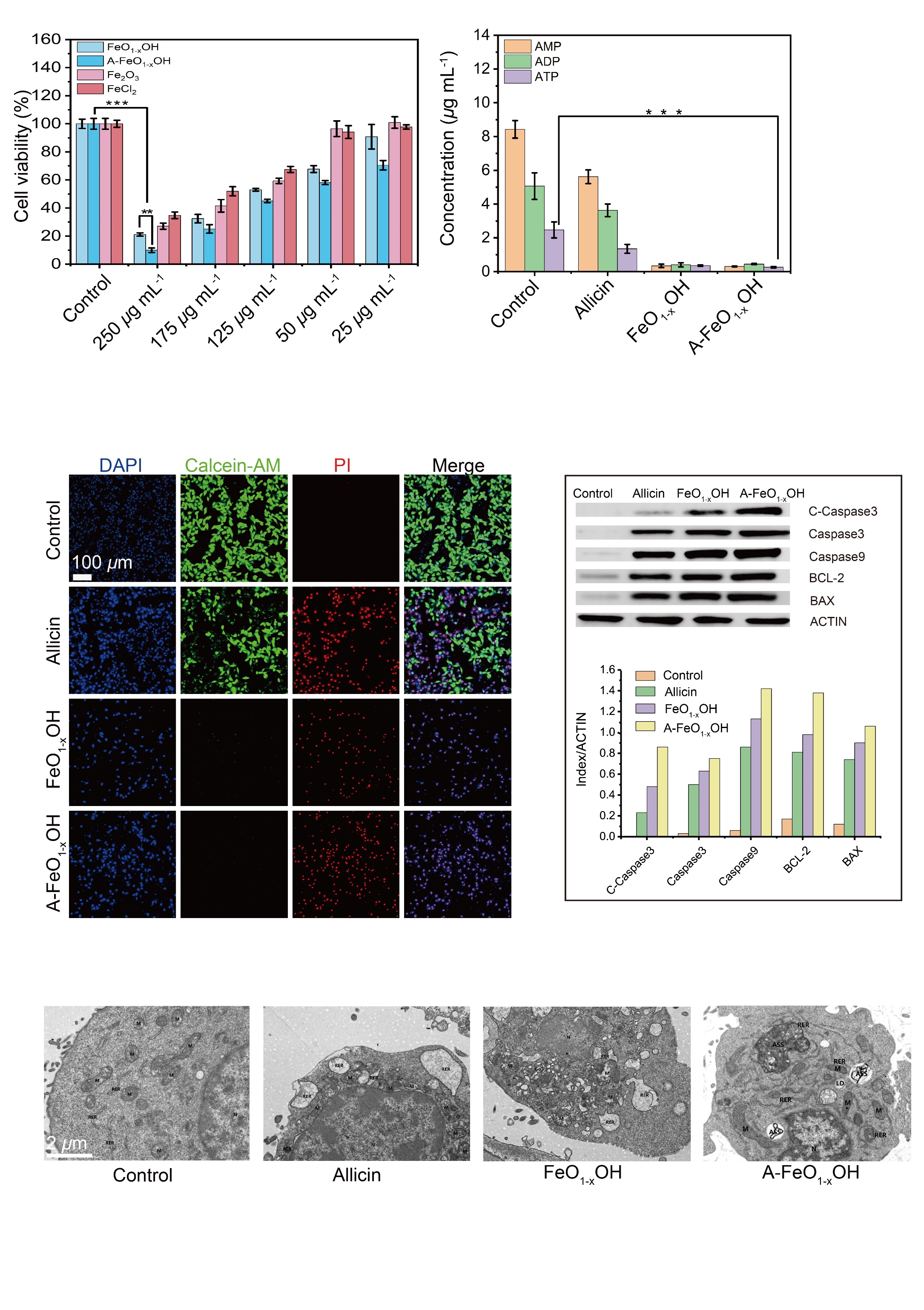


**Figure S27.** The ATP concentrations in 4T1 cells treated with allicin, FeO_1-x_OH, and A‒FeO_1-x_OH for 24 h. The results are reported as means standard deviation (*n* = 3). *p < 0.05, **p < 0.01, ***p < 0.001.


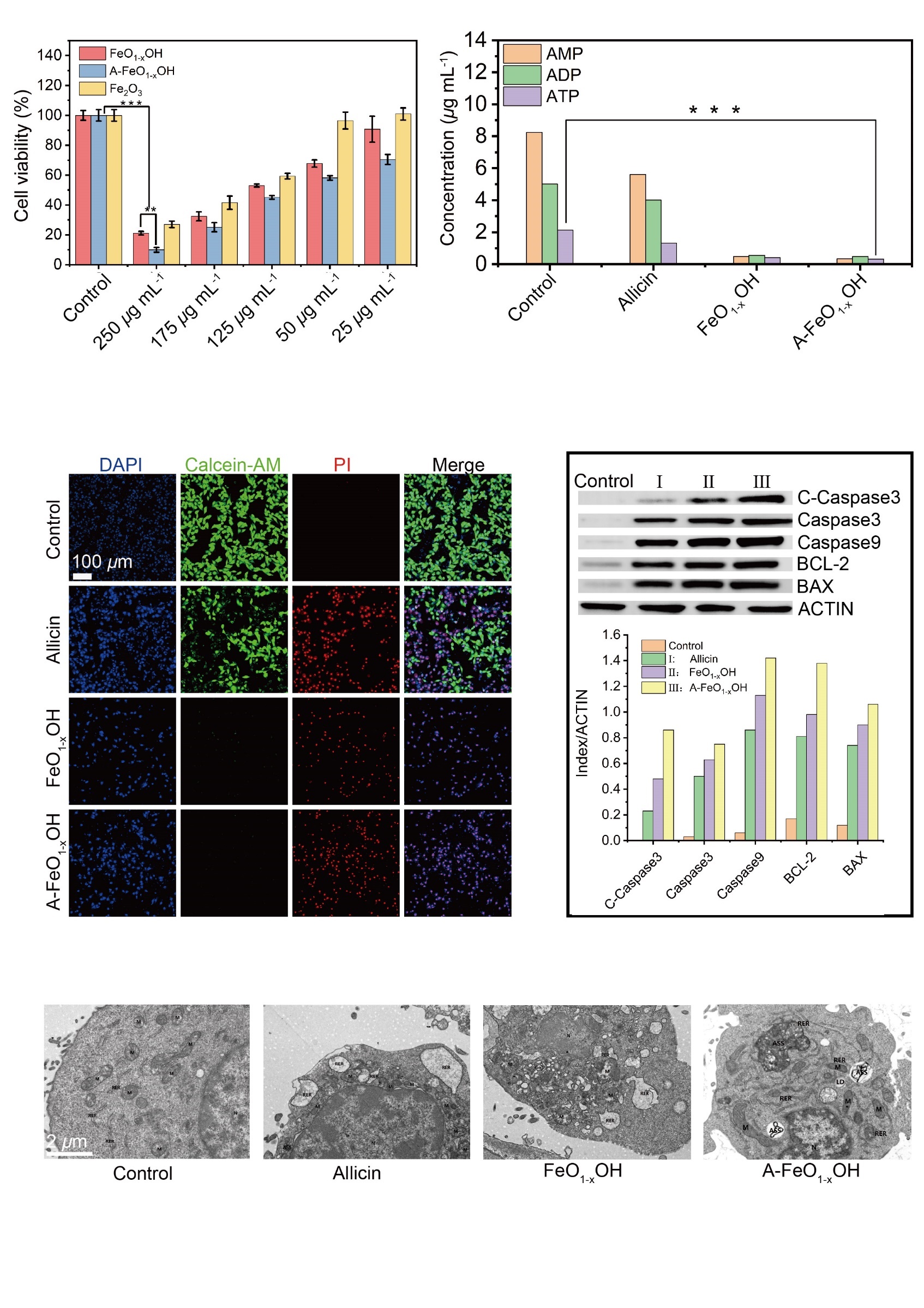


**Figure S28.** CLSM images of DAPI, Calcein‒AM/PI staining of 4T1 tumor cells after co‒incubation with allicin, FeO_1-x_OH and A‒FeO_1-x_OH for 3 h.


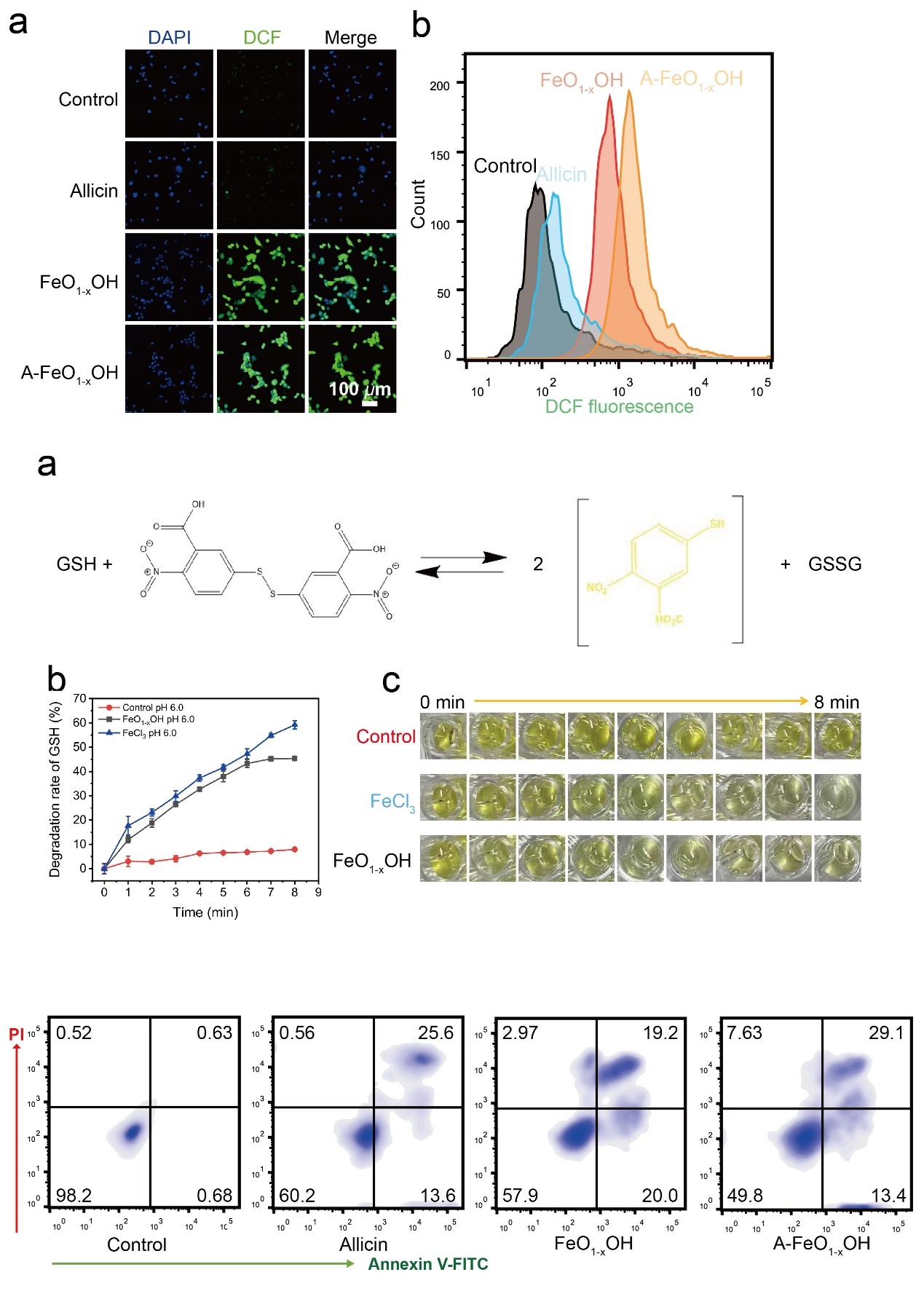


**Figure S29.** Cell apoptosis induced by allicin, FeO_1-x_OH and A‒FeO_1-x_OH are examined via flow cytometry. The cells are stained with Annexin V‒FITC and PI.


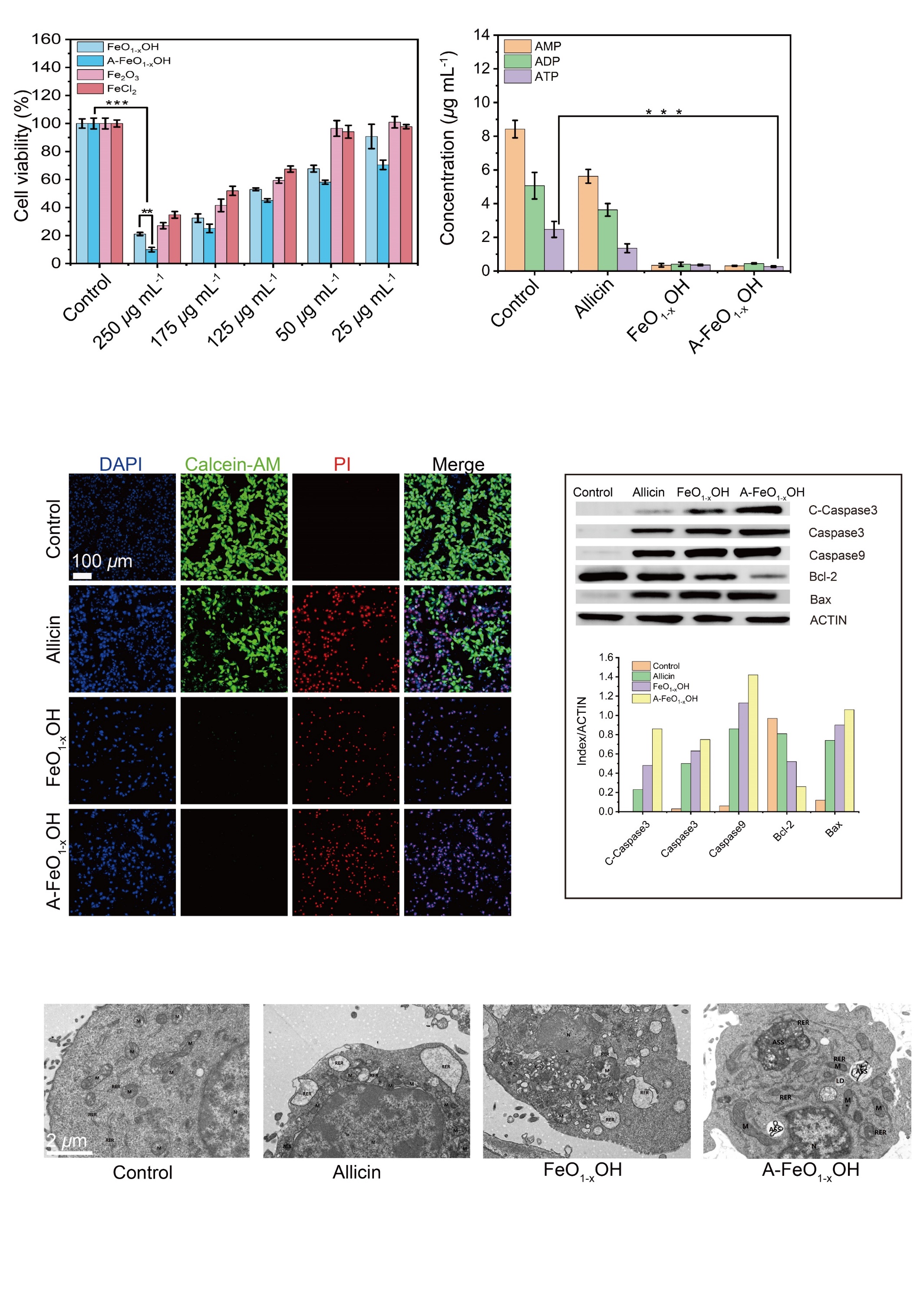


**Figure S30.** WB analysis of C‒Caspase3, Caspase3, Caspase9, Bcl‒2, Bax of 4T1 cells co‒incubated with allicin, FeO_1-x_OH and A‒FeO_1-x_OH for 24 h.


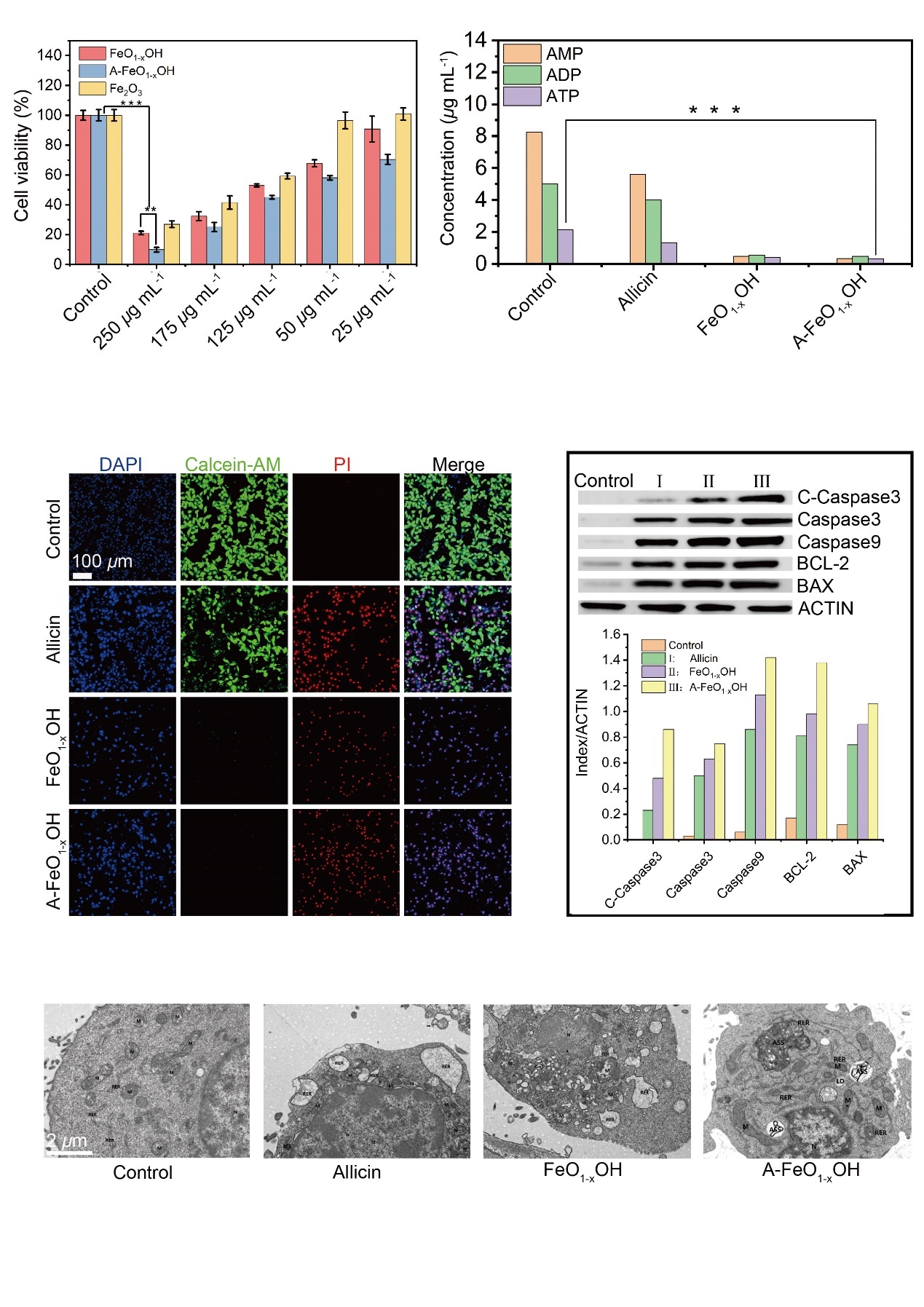


**Figure S31.** Bio‒TEM observation of 4T1 tumor cells treated with allicin, FeO_1-x_OH and A‒FeO_1-x_OH.


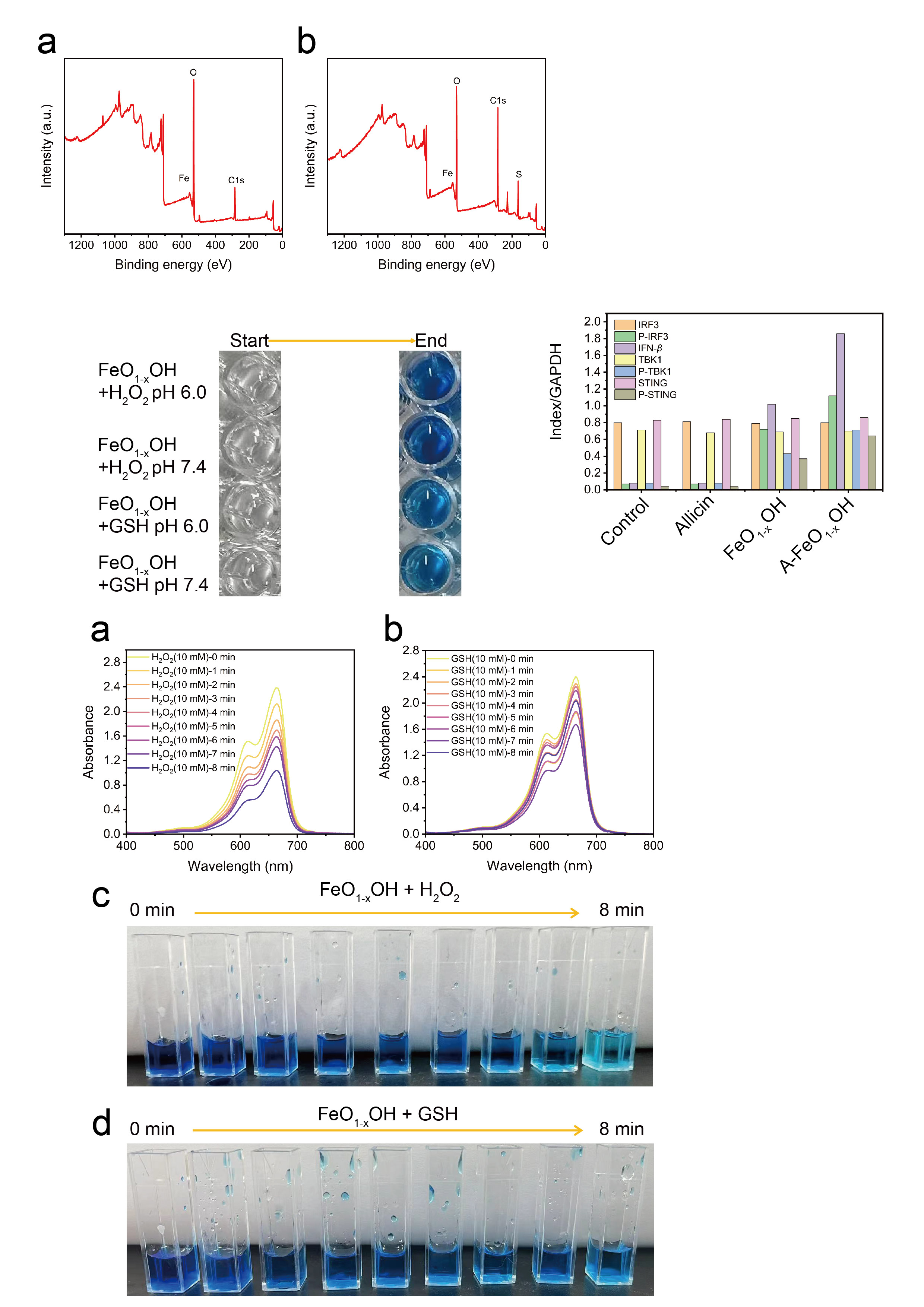


**Figure S32.** WB analysis of IRF3, P‒IRF3, IFN‒*β*, TBK1, P‒TBK1, STING, P‒STING proteins with varied conditions.


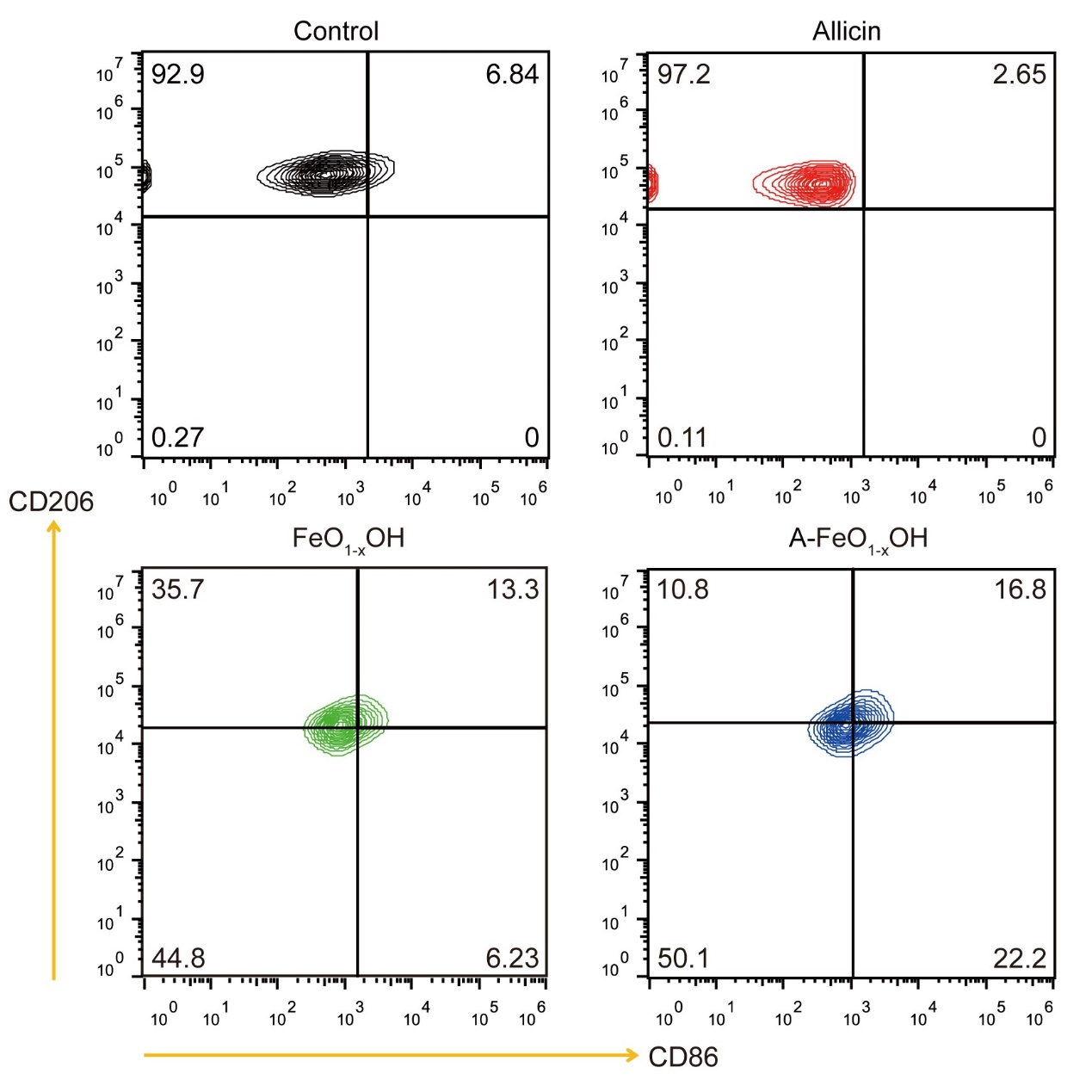


**Figure S33.** Flow cytometry plots of pro‒inflammatory M1 phenotype and M2 phenotype for macrophages co‒cultured with 4T1 cancer cells undergone various treatments.


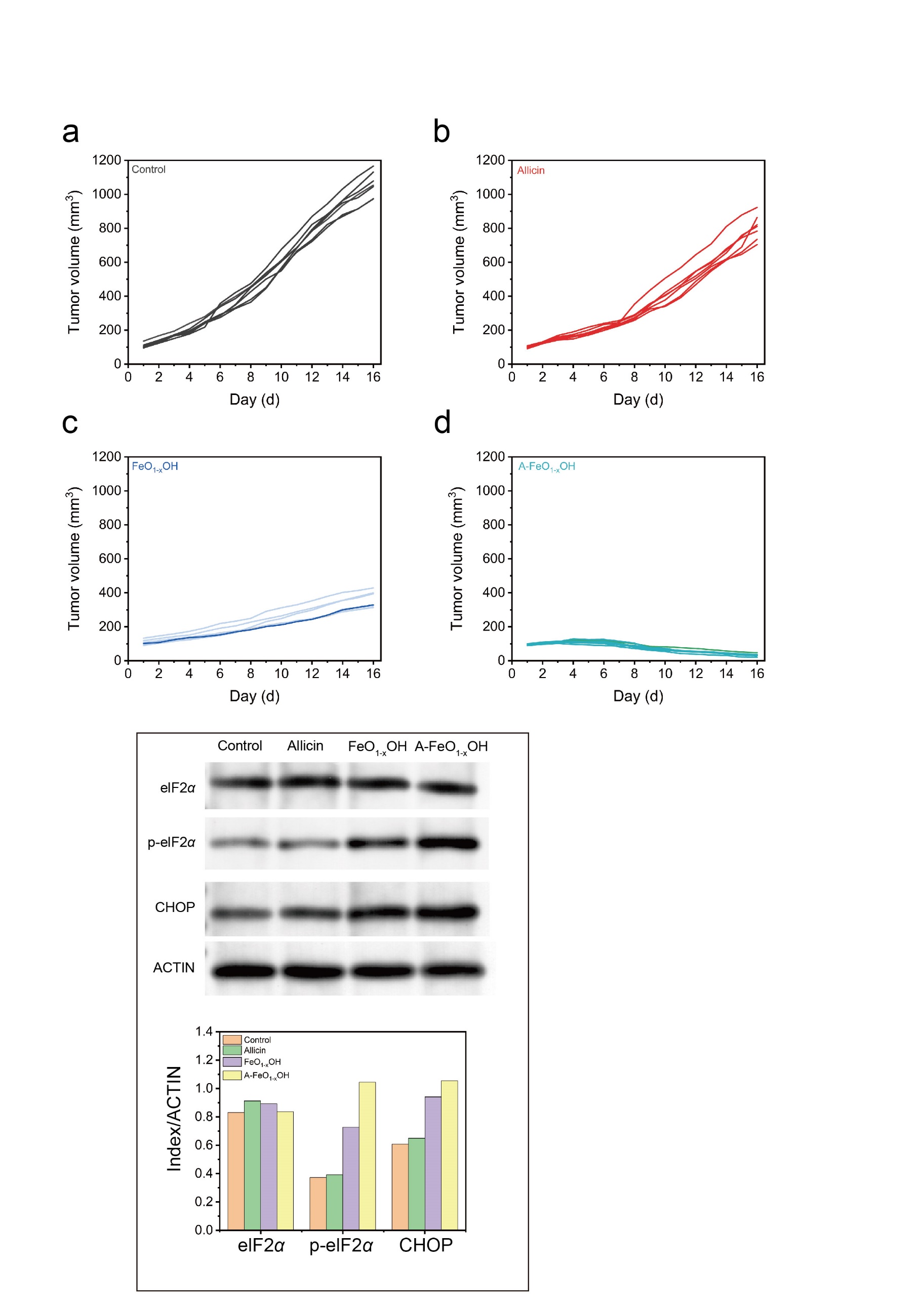


**Figure S34.** WB analysis of ER stress-related proteins in 4T1 cells after treatment with allicin, FeO_1-x_OH and A‒FeO_1-x_OH for 24 h.


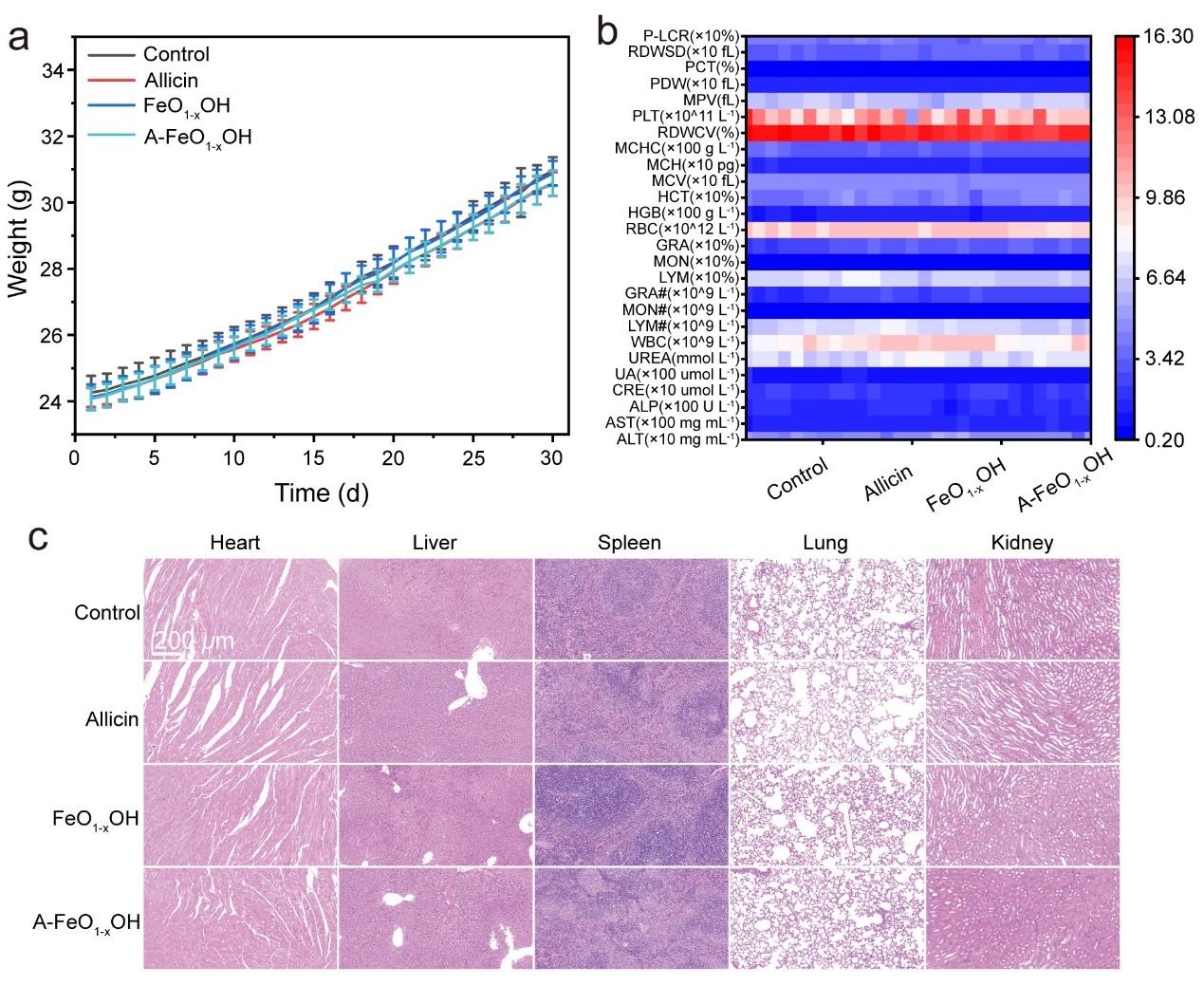


**Figure S35.** a) The body weight of the mice (*n* = 7) injected with normal saline (control), allicin, FeO_1-x_OH, A‒FeO_1-x_OH during 30 days. b) After a 30‒day treatment period, assessments are performed on the blood routine and blood biochemistry of the mice. c) Histopathological images using H&E staining are captured for the heart, liver, spleen, lung, and kidney of mice subjected to a 30‒day treatment.


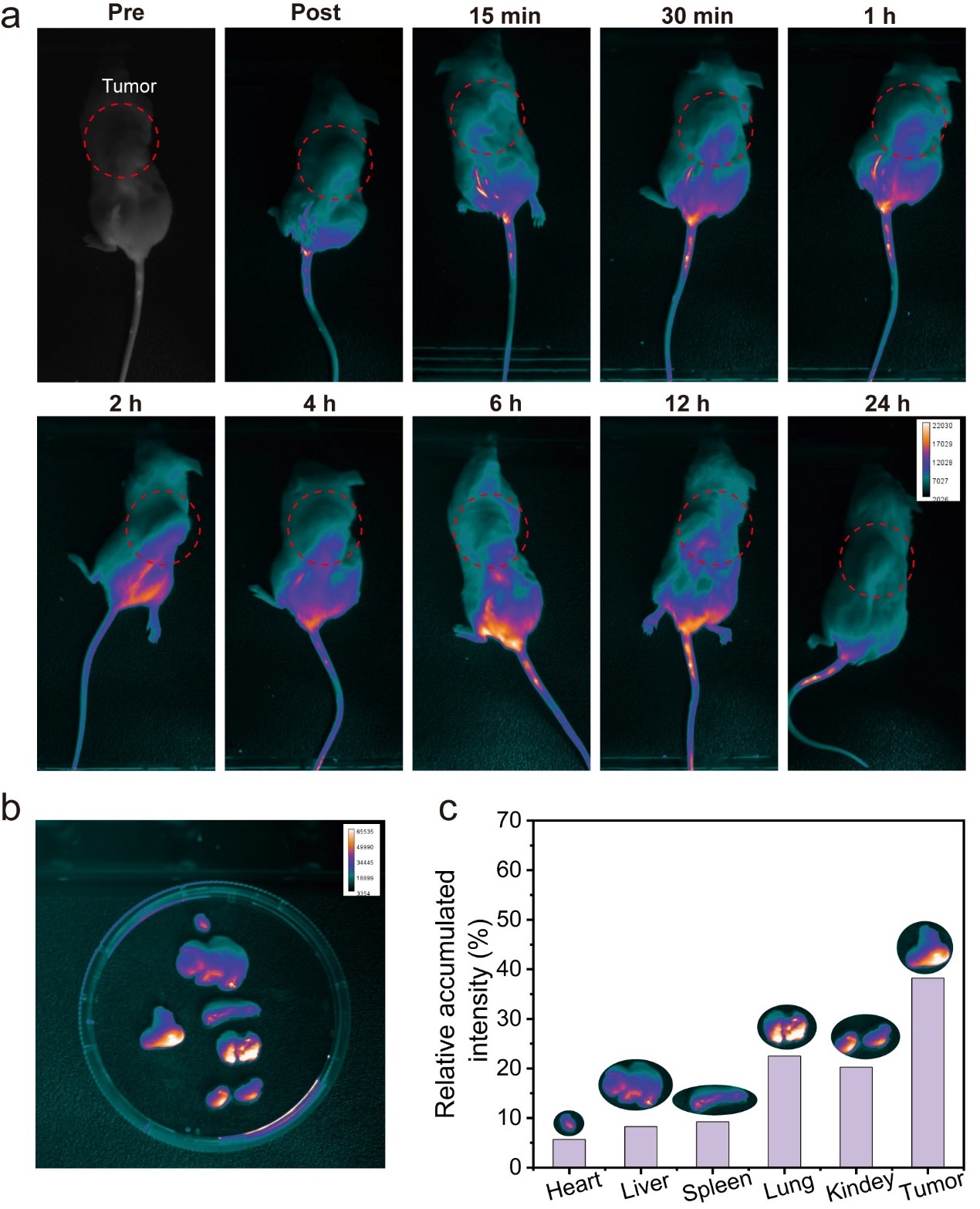


**Figure S36.** a) Fluorescence images of in vivo biological distribution of drugs at different time points after injection of Cy5.5 coupled A‒FeO_1-x_OH in 4T1 tumor‒bearded mice. b, c) Fluorescence images of heart, liver, spleen, lung, and kidney tumors (b) and corresponding fluorescence intensities after injected with nanomedicine (c).


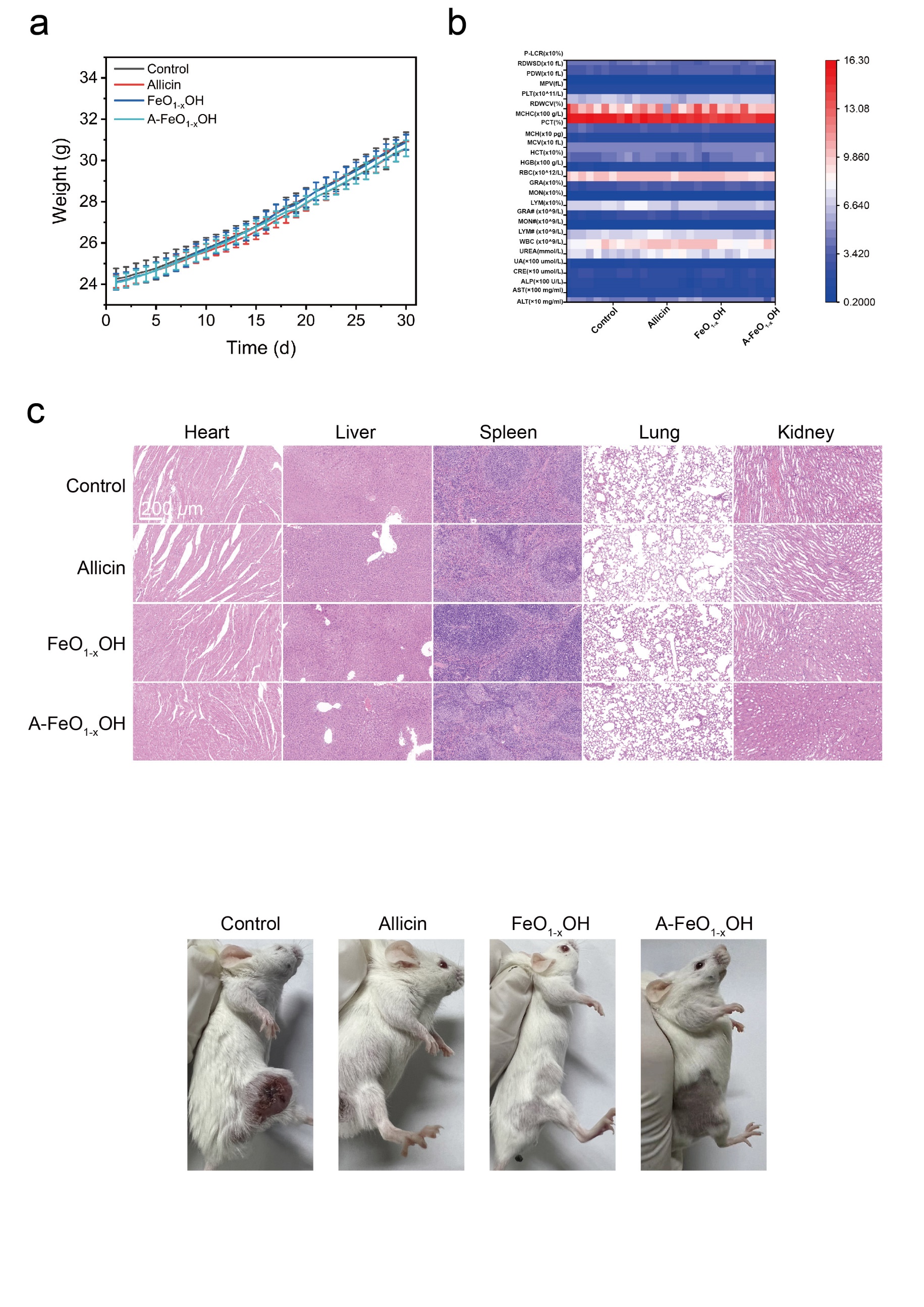


**Figure S37.** Digital photos of the mice treated with normal saline (control), allicin, FeO_1-x_OH, and A‒FeO_1-x_OH in 16 days.


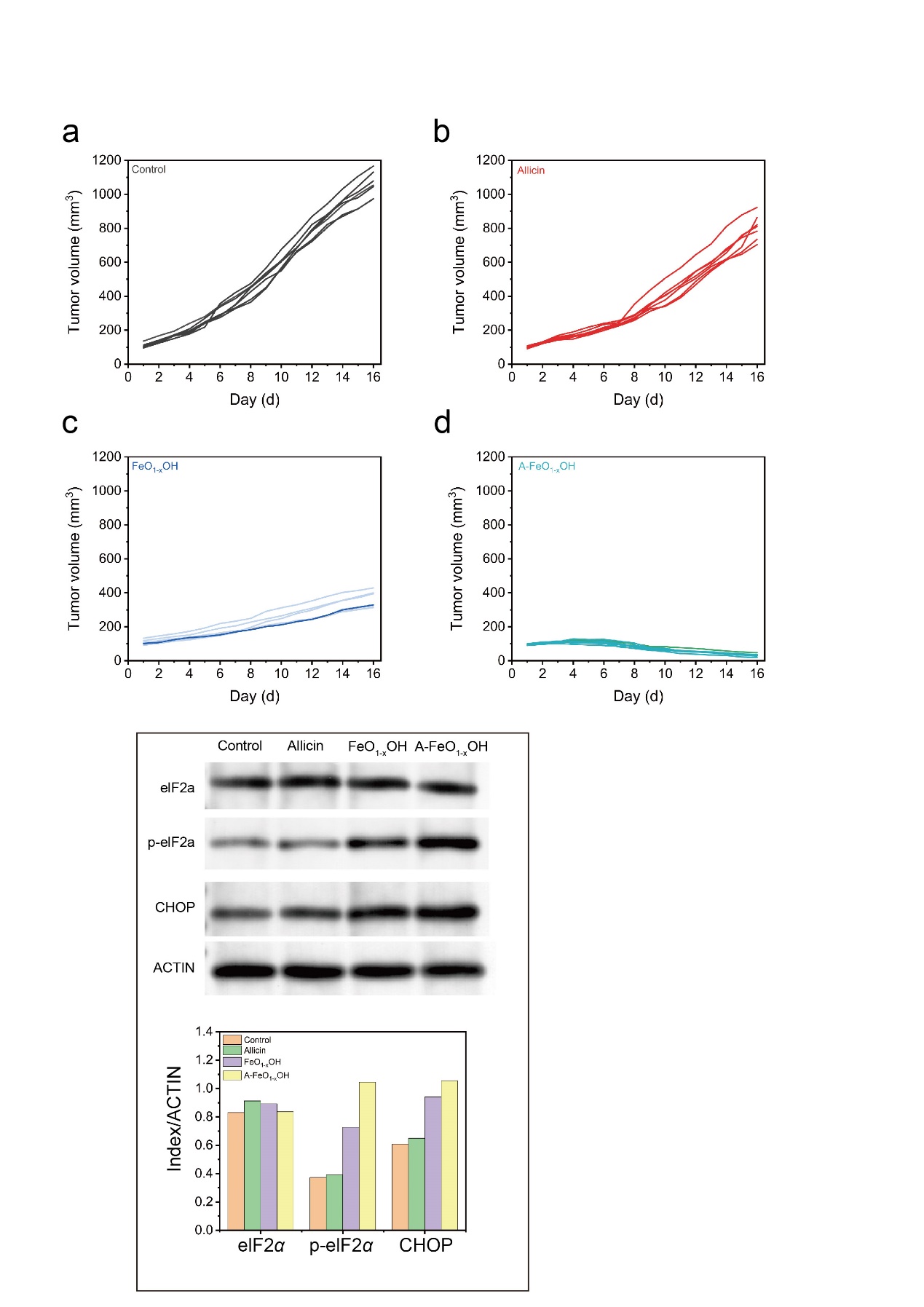


**Figure S38.** Tumor volume growth curves for each mouse in all groups.


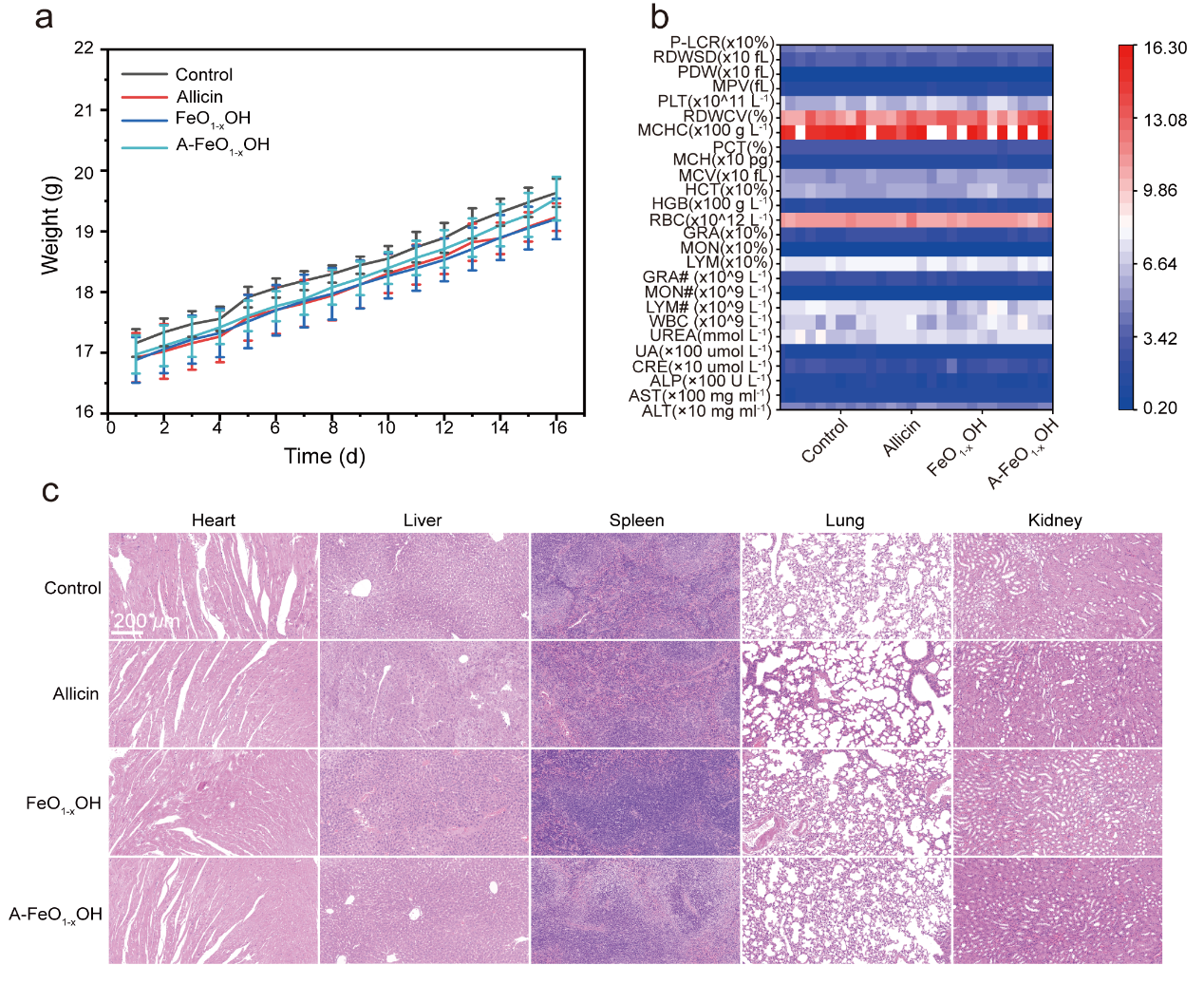


**Figure S39.** a) Body weight statistics over time during the 16‒day treatment period (*n* = 7). 4T1‒tumorbearing mice are injected with normal saline (control), allicin, FeO_1-x_OH, A‒FeO_1-x_OH. b) Routine hematological biochemical analysis. The blood is taken from inner canthus of the mice after treatment for 16 days. c) H&E section scanning images for heart, liver, spleen, lung, and kidney from mice after treatment for 16 days.


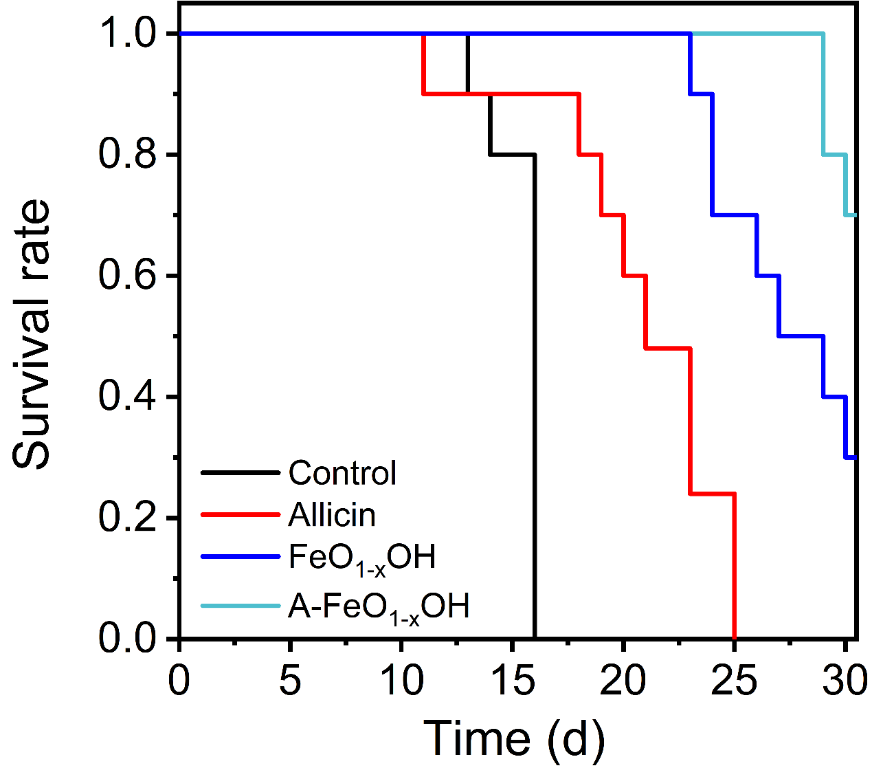


**Figure S40.** Survival rate curves of 4T1‒tumor-bearing mice after different treatments.

**
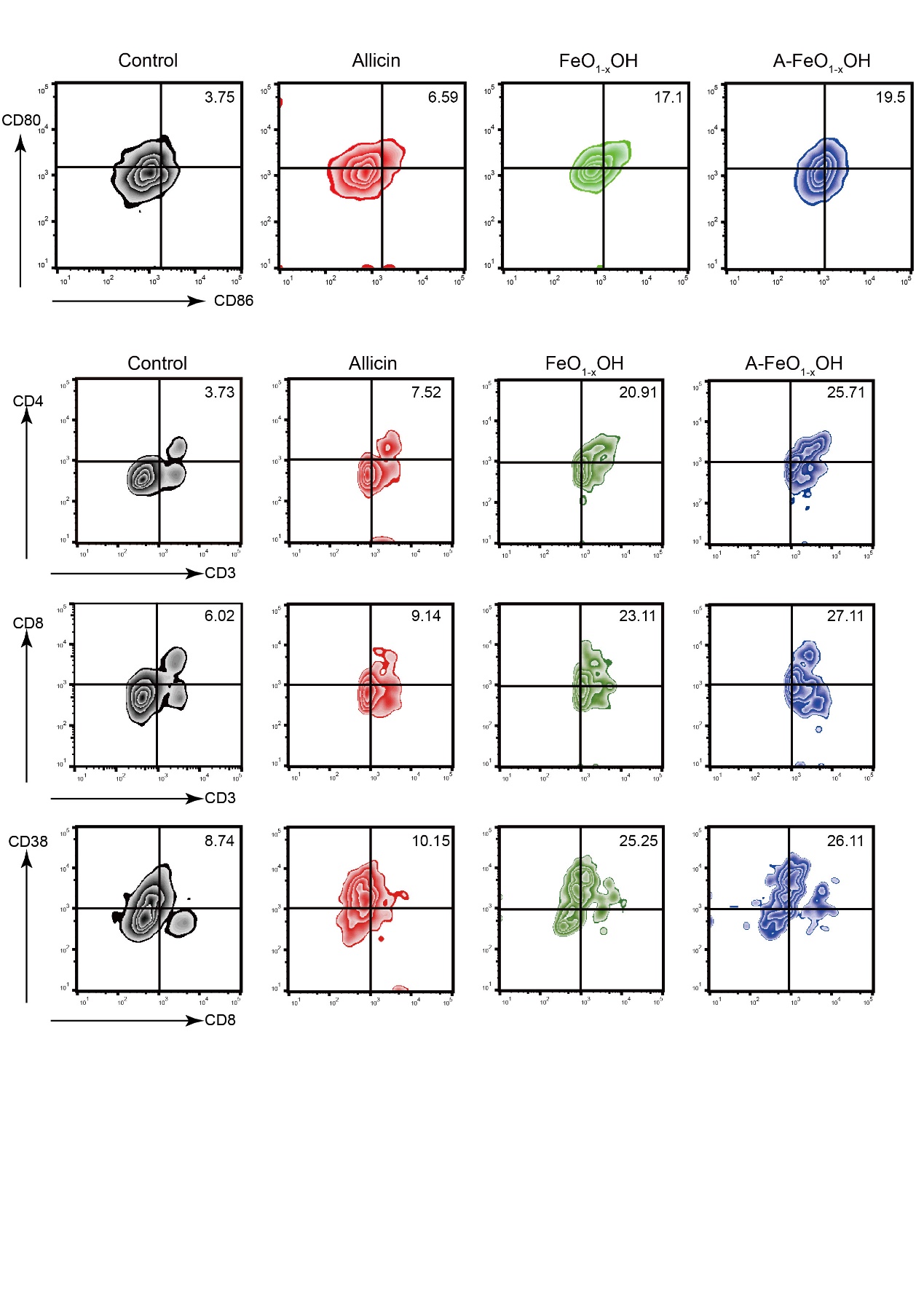
**

**Figure S41.** An investigation of matured DCs expressing (CD80^+^CD86^+^CD11c^+^) markers in 4T1‒tumor-bearing mice utilizing flow cytometry analysis.


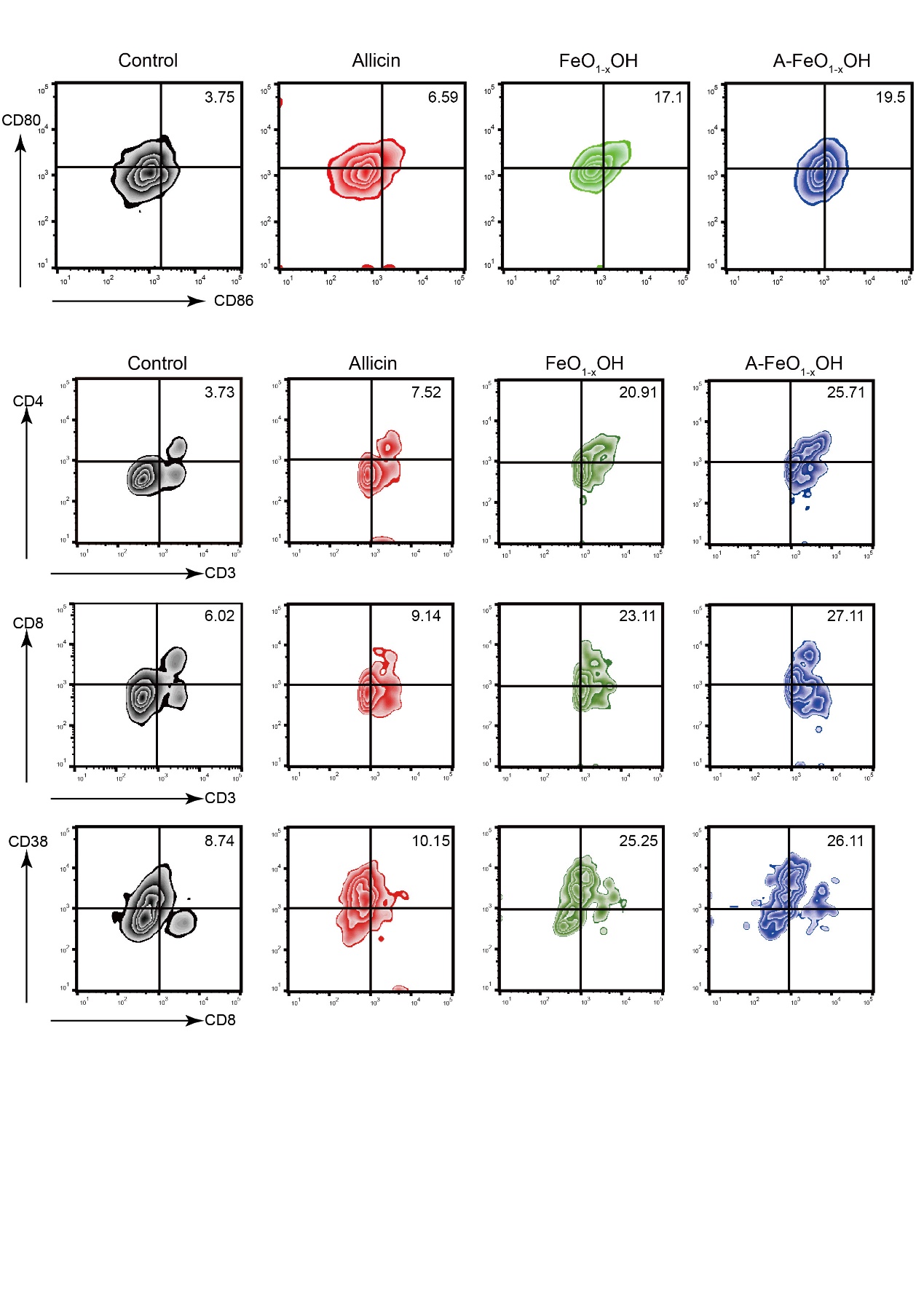


**Figure S42.** Illustrative flow cytometry plots (%) of helper T lymphocytes (CD3^+^CD4^+^), cytotoxic T lymphocytes (CD3^+^CD8^+^), and activated cytotoxic T lymphocytes (CD3^+^CD8^+^CD38^+^) in the primary tumor in different treatment groups.


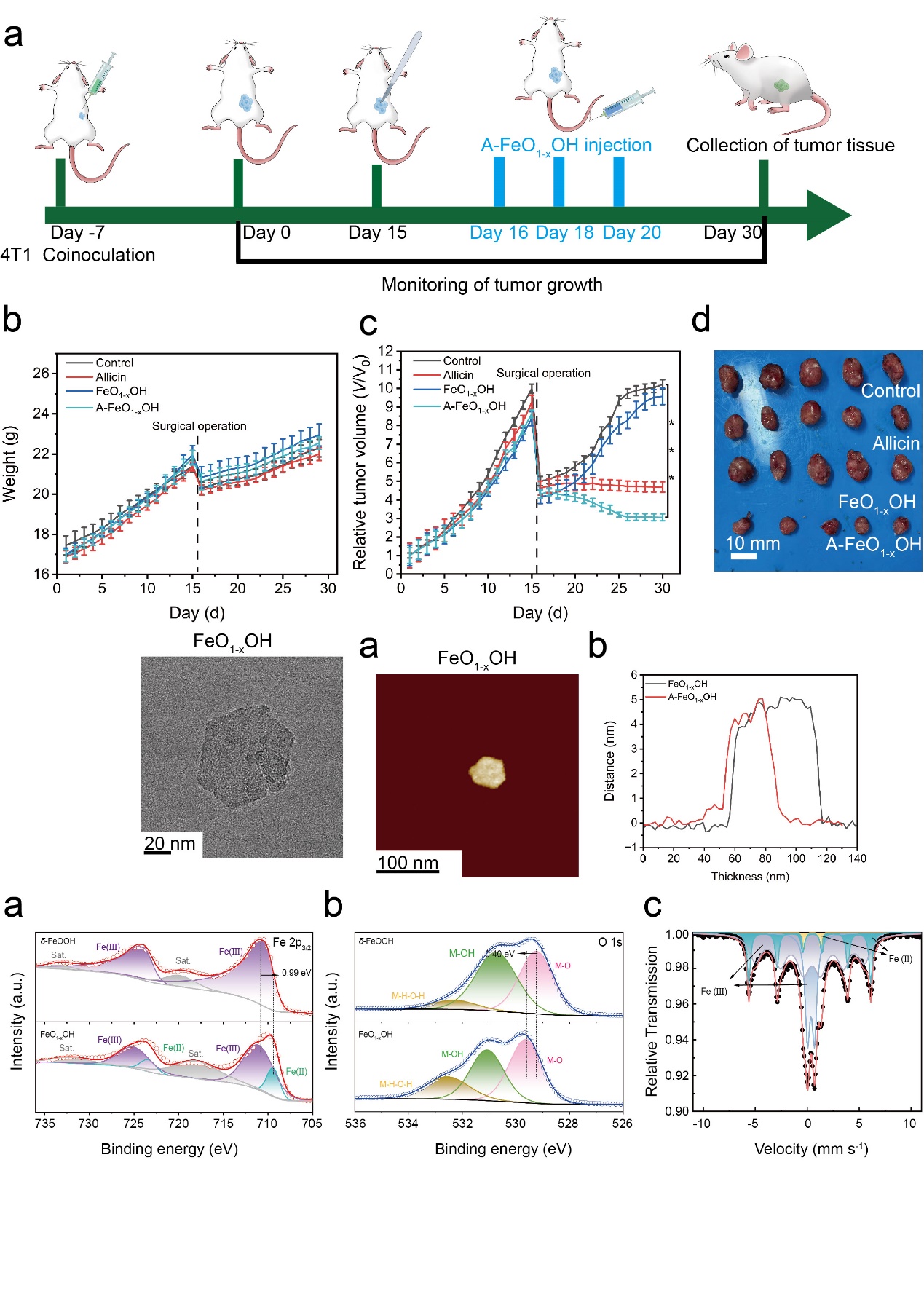


**Figure S43.** A‒FeO_1-x_OH anti‒tumor recurrence treatment. a) Schematic diagram of anti‒tumor recurrence treatment. Half of the tumor in mice was removed by surgical treatment in day 15, and A‒FeO_1-x_OH was injected in day 16, 18, and 20. b) Mouse weight during the anti‒tumor recurrence treatment (*n* = 7, *p < 0.05, **p < 0.01, and ***p < 0.001). c) Tumor volume change during anti‒tumor recurrence treatment (*n* = 7, *p < 0.05, **p < 0.01, and ***p < 0.001). d) Digital photo of tumors in different groups after anti‒tumor recurrence treatment.


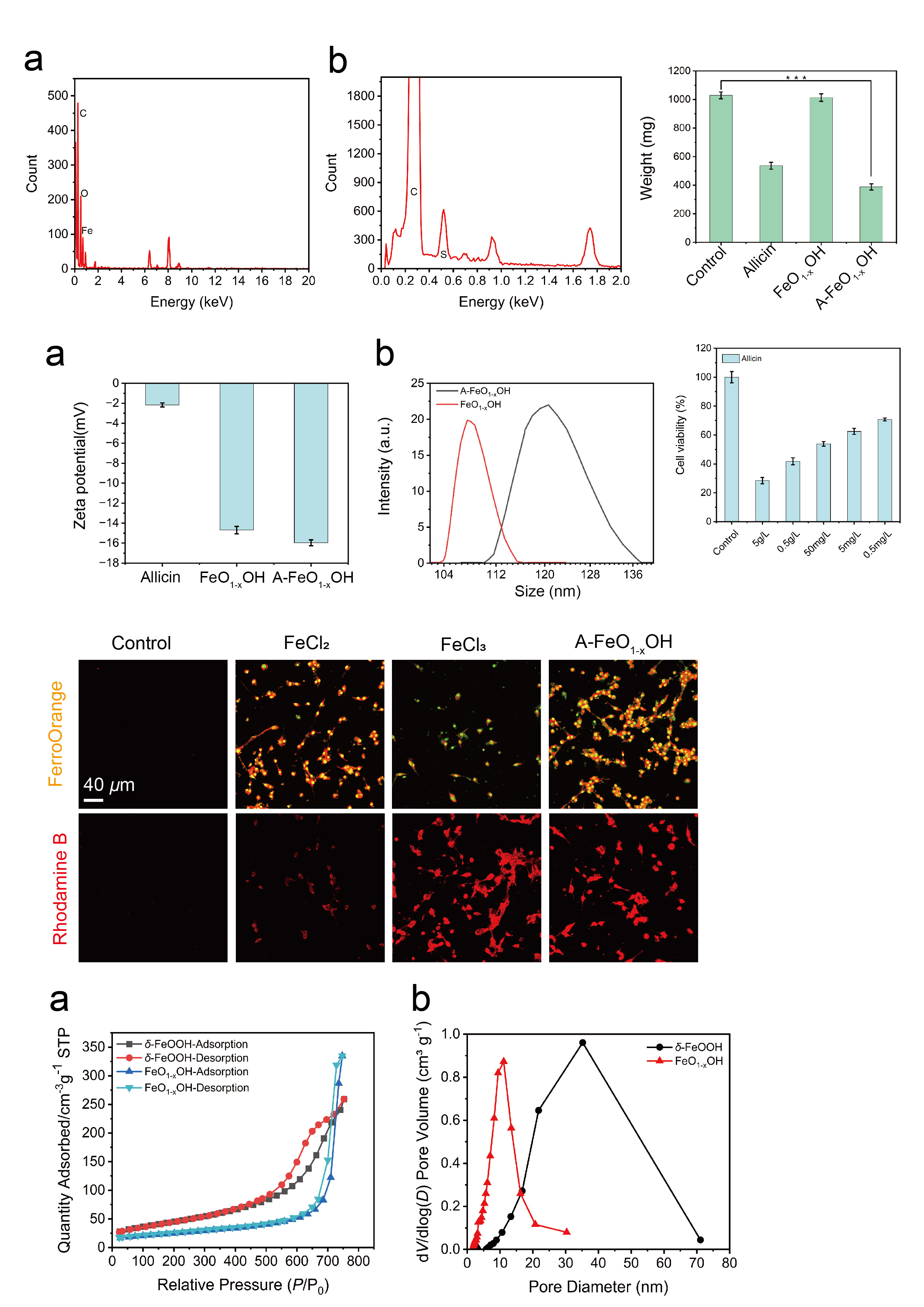


**Figure S44.** Weight data of the tumor after treatment for 30 days.

**Supplementary Tables**

**Table S1.** Fe (III)/Fe (II) ratio in different samples

| Sample | FeO_1-x_OH | A‒FeO_1-x_OH |
| --- | --- | --- |
| Fe (III:II) | 5:1 | 4.8:1 |

**Table S2.** ICP‒OES elemental content analysis of A‒FeO_1-x_OH

| Fe (m.%) | S (m.%) |
| --- | --- |
| 55.28 | 4.9 |

**Table S3.** *E*_0_ values of *δ*‒FeOOH, FeO_1-x_OH and FeCl_2_

| Sample | *δ*‒FeOOH | FeO_1-x_OH | A‒FeO_1-x_OH | FeCl_2_ |
| --- | --- | --- | --- | --- |
| *E_0_* value (eV) | 7130.9185 | 7130.4206 | 7128.2109 | 7121.4588 |

**Table S4.** The fitting parameters for the EXAFS data at the Fe *K*-edge of the samples

| Sample | Path | *CN*^a^ | *R* (Å)^b^ | *σ*^2^ (Å^2^)^c^ |  | *ΔE_0_* (eV)^d^ | | *R* factor |
| --- | --- | --- | --- | --- | --- | --- | --- | --- |
| *δ*‒FeOOH | Fe‒O | 6* | 1.50940±0.041 | 0.01395±0.0012 |  | | 4.3±1.8 | 0.0068 |
|  | Fe‒OH | 2* | 2.60000±0.031 | 0.01504±0.0020 |  | | 3.7±1.5 | 0.0077 |
| FeO_1-x_OH | Fe‒O | 6* | 1.53394±0.033 | 0.0109±0.0028 |  | | 3.1±1.3 | 0.0135 |
|  | Fe‒OH | 2* | 2.51000±0.028 | 0.0098±0.0035 |  | | ‒2.6±3.6 | 0.0235 |
| A‒FeO_1-x_OH | Fe‒O | 6* | 1.56404±0.027 | 0.0109±0.0028 |  | | 3.2±1.4 | 0.0139 |
|  | Fe‒OH | 2* | 2.61010±0.034 | 0.0095±0.0031 |  | | ‒2.5±3.7 | 0.0255 |
| FeCl_2_ | Fe‒Cl | 6* | 1.99326±0.0411 | 0.0127±0.0050 |  | | 2.4±9.2 | 0.0149 |

The provided information outlines various parameters for EXAFS analysis:

*^a^CN* (coordination number): *^a^CN* represents the number of atoms surrounding the central atom in a coordination complex.

*^b^R* (distance to the neighboring atom): *^b^R* indicates the distance from the central atom to its neighboring atom.

*^c^σ*^2^ (Mean Square Relative Displacement‒MSRD): *^c^σ*^2^ is the measure of the average squared displacement of atoms from their equilibrium positions.

*^d^ΔE_0_* (inner potential correction): *^d^ΔE_0_*, represents the inner potential correction applied during the EXAFS analysis.

*R* factor (goodness of fit): The *R* factor is a statistical measure indicating the goodness of fit between the theoretical model and the experimental data. A lower *R* factor signifies a better fit.

*S_0_*^2^ (fixed to 0.718): *S_0_*^2^ is the amplitude reduction factor, and it was fixed at a value of 0.718 during the EXAFS fitting. This fixed value is based on the known structure of *δ*‒FeOOH, FeCl_2_.

Ranges for different samples:

For *δ*‒FeOOH: *k* (Å) between 3.0 and 8.0, *R* (Å) between 1.0 and 3.0.

For FeO_1-x_OH: *k* (Å) between 3.0 and 8.0, *R* (Å) between 1.0 and 3.0.

For A‒FeO_1-x_OH: *k* (Å) between 3.0 and 8.0, *R* (Å) between 1.0 and 3.0.

For FeCl_2_: *k* (Å) between 2.7 and 10.7, *R* (Å) between 1.0 and 3.4.

Reasonable range for fitting parameters:

0.700 < *S_0_*^2^ < 1.000

*CN* > 0

*σ*^2^ > 0 Å^2^

|*ΔE_0_*| < 10 eV

*R* factor < 0.02 (*S_0_*^2^ = 0.718)

**Table S5**. Primers used in RT‒qPCR measurement

| Primer information | Primers | Primer sequences (5’‒3’) | Fragment length (bp) | Annealing temperature (°C) |
| --- | --- | --- | --- | --- |
| NM_008084.2 | M‒GAPDH‒S | CCTCGTCCCGTAGACAAAATG | 133 | 60 |
|  | M‒GAPDH‒A | TGAGGTCAATGAAGGGGTCGT |  | 60 |
| NM_016756.4 | M‒CDK2‒S | CTCTCACGGGCATTCCTCTT | 123 | 60 |
|  | M‒CDK2‒A | TGATAAGCAGGTTCTGGGGC |  | 60 |

**Experimental part**

**Materials and reagents**

FeCl_2_·4H_2_O (≥ 99.0%) was purchased from Sigma‒Aldrich. H_2_O_2_ (30%) was purchased from Sinopharm Chemical Reagent. Fe powder (99%), C11‒bodipy^581/591^, DTNB, Fe^2+^ Fluorescent Probe FerroOrange, Fe^3+^ fluorescent probe Rhodamine B and Cy5.5 succinimide lipid were purchased from Thermo Fisher. The allicin, MB (≥ 98%), PBS, and DMSO (≥ 99.70%) were purchased from Macklin. DSPE‒PEG‒NH_2_, reduced GSH, and TMB were purchased from Aladdin. The DAPI staining solution, cell CCK‒8 kit, reactive oxygen species assay kit, Annexin V‒FITC apoptosis‒detection kit, Calcein/PI cell viability/cytotoxicity assay kit, EdU kit, crystalline purple, and MDA assay kit was purchased from Beyotime. The ATP detection kit was purchased from Solarbio. The DMPO was purchased from the Shanghai Titan company (Shanghai, China).

**Synthesis of *δ*‒FeOOH**

Added FeCl_2_·4H_2_O (5.9640 g) to 300 mL deionized water to form a solution (concentration of Fe^2+^ was 0.1 mol L^-1^). The pH of the prepared solution was adjusted to ~8.0 by adding NaOH solution (5.0 mol L^-1^). Then, 40 mL of 30% H_2_O_2_ was rapidly added, the ferrous hydroxide was rapidly oxidized and precipitated with a reddish‒brown color in a few seconds. Due to the release of H^+^ ions, the pH value was decreased. To improve the flocculation effect and adjust the pH of the solution to ~8.0, 5.0 mol L^‒1^ NaOH was dropwise added under stirring for 1 h. Afterward, the solution was centrifuged (18759 g for 5 min) and washed three times using deionized water. Afterwards, *δ*‒FeOOH was obtained by freeze-drying.

**Synthesis of FeO_1-x_OH**

*δ*‒FeOOH (2.0000 g) was added to 100 mL deionized water to form a solution. Reduced iron powder (0.5000 g) was added and stirred for 30 min. After 30 min, a magnet was put onto the outer wall of the beaker to attract and removed the unreacted Fe powders. The reaction mixture should be filtrated, and the resulting product should be washed with deionized water for three times, centrifugated (18759 g for 5 min) and freeze dried. Finally, *δ*‒FeOOH powder was obtained.

**Synthesis of A****‒FeO_1-x_OH**

FeO_1-x_OH (1.0000 g) was dispersed in 60 mL of deionized water and stirred for 30 minutes. DSPE‒PEG‒NH_2_ (0.0400 g) was dissolved in 30 mL of deionized water and stirred for 30 minutes. DSPE‒PEG‒NH_2_ was dropwise added into the FeO_1-x_OH solution and stirred for 12 h in the dark. Modified FeO_1-x_OH was obtained after centrifugated with deionized water (18759 g for 5 min) and washed for three times.

The altered FeO_1-x_OH was introduced into a solution consisting of 15 mL of dimethyl sulfoxide and 15 mL of deionized water, followed by stirring for 30 minutes. The allicin (0.1000 g) was dissolved in dimethyl sulfoxide (15 mL) and stirred for 30 min to prepare a solution. The allicin solution was dropwise added to the modified FeO_1-x_OH solution and stirred for 12 h in the dark. After 12 h, centrifuged (18759 g, 5 min) and washed with deionized water for three times. Finally, lyophilization was used for collecting A‒FeO_1-x_OH, and the sample was obtained.

**Degradation of A‒FeO_1-x_OH and release of allicin**

A‒FeO_1-x_OH (0.0100 g) was dispersed in PBS solution at pH 6.0 or pH 7.4, respectively, with magnetic stirring. At different time points, 1 mL solution was extracted with a pipette gun and centrifuged. The supernatant obtained by centrifugation was used to determine the amount of Fe and S elements by ICP‒OES, and then the release rates were calculated. The precipitate was dispersed in deionized water for the sample preparation and observed under TEM.

**Characterization methods**

TEM observation was carried out using a TEM FEI TECNAI F20 electron microscope (200 kV), and the corresponding EDX was recorded on a JEOL ARM‒300F. AC‒TEM was carried out using an FEI Themis Z (300 kV). Bio‒TEM observation was carried out using a FEI TECNAI spirit (80 kV). AFM test was performed using a Bruker Dimension Icon. The Mossbauer spectra results were obtained using an MS500. EXAFS measurement was carried out using easyXAFS300. XPS analysis was performed using K‒Alpha (Thermo Fisher). An Ultima IV X‒ray diffractometer (parameters: Cu‒K*α*, *λ* = 1.54 Å, 40 mA, 40 kV) was used to perform XRD measurements. The Zeta potential and DLS measurements were performed using Nano ZS90, Malvern Instruments Co., Ltd. The machine model used for UV test was UV-3600. FT‒IR (Perkin Elmer spectrum GX) and Raman spectroscopy (LabRAM HR evolution) was used to obtain FT‒IR and Raman spectra. ESR characterization was performed using a Bruker EMX Plus instrument. CLSM observation was operated on Olympus FV3000. The Flow cytometry results were obtained using BD LSR Fortessa. The intravital fluorescence was measured using a PerkinElmer IVIS lumina x RMS (USA). The O_2_ concentration was measured using a Unisense oxygen microelectrode. The cyclic voltammetry was measured using a CHI760E instrument. RT‒qPCR detection used QuantStudio 7 Flex. IC‒ICP‒MS determination of Fe^2+^, Fe^3+^ used ICS 2100 (Thermo Scientific)‒iCAP RQ ICPMS (Thermo Scientific).

**Evaluation of the Fenton and the GSH oxidation reactions**

**The ESR determination**

The DMPO can form a DMPO‒OH complex with a hydroxyl radical. Therefore, DMPO is a general hydroxyl radical‒trapping reagent. DMPO (5 *μ*L) was added to a solution (900 *μ*L) contained GSH (10 mM), H_2_O_2_ (10 mM), followed by 100 *μ*L FeO_1-x_OH (250 *μ*g mL^-1^). The solution was transferred to a quartz tube for ESR determination after vigorous spinning for 5 min.

**The valence‒change determination**

FeO_1-x_OH (250 *μ*g mL^-1^) was added to a PBS (pH 6.0) solution contained GSH (20 mM), H_2_O_2_ (20 mM), and reacted for 30 min. After centrifugation and vacuum drying, the valence changes of Fe were analyzed using XPS.

**The MB‒fading experiment**

The methylene blue (12.5 mg L^-1^), GSH (10 mM), H_2_O_2_ (10 mM) were added to PBS (pH 6.0) to form a reaction substrate solution (2 mL), followed by adding of FeO_1-x_OH (250 *μ*g mL^-1^). The absorbance value of the solution was observed at 660 nm using a quartz cell at different time points (0~8 min).

**The GSH depletion capacity of FeO_1-x_OH**

The DTNB was used to determine the GSH content at 412 nm. In a buffer solution system (PBS, pH 6.0) contained GSH (10 mM) and FeO_1-x_OH (250 *μg* mL^-1^), the absorbance value of the solution was read at 412 nm wavelength using a 96‒well plate, and the absorbances at 412 nm at different time points were recorded at 25℃.

**The TMB color rendering capacity of FeO_1-x_OH**

A 96‒well plate was used to read the data at a time interval of 1 s at 450 nm. The experiment was carried out in the buffer solution system (PBS, pH 6.0 or pH 7.4) contained TMB (25 mg L^-1^), GSH (10 mM), H_2_O_2_ (10 mM) and FeO_1-x_OH (250 *μg* mL^-1^). The absorbance at 450 nm was recorded at 25°C for 10 min.

**Quantitative detection for O_2_ concentration**

A Unisense oxygen microelectrode was used to detect the concentration of O_2_ in the buffer solution system (PBS, pH 6.0 or 7.4) contained FeO_1-x_OH (250 *μ*g mL^-1^) and GSH (10 mM) or H_2_O_2_ (30%). The O_2_ concentration was measured 2 cm below the liquid level for 30 min.

**Electrochemical analysis**

For electrochemical analysis, 0.0050 g of FeO_1-x_OH, 1 mL of H_2_O, 50 *μ*L of Nafion solution, and 1 mL of ethanol were thoroughly mixed and sonicated for 30 minutes before being applied onto a glassy carbon electrode. The AgCl/Ag (with a liquid inner component of 1 mol L^-1^ KCl) electrode was served as the reference electrode, and a carbon rod was served as the counter electrode. The electrolyte was PBS, with a pH of 6.0 or pH 7.4, containing either GSH (10 mM) or H_2_O_2_ (10 mM). The potential range was set at -1.60 to 1.60 V vs. AgCl/Ag, and the scan rate was 1 mV s^-1^.

**EXAFS measurement**

EXAFS Fe *K*‒edge data for the *δ*‒FeOOH, FeO_1-x_OH, FeCl_2_ were collected in transmission mode on easyXAFS300 system at room temperature, calibrated background, leading and trailing edge lines in Athena soft (version 0.9.26). Fourier transform fitting was performed in Artemis soft (version 0.9.26).^[4]^ Four parameters, namely coordination number (*CN*), bond length (*R*), Debye-Waller factor (*σ^2^*), and *E_0_* shift (*ΔE_0_*), were subjected to fitting with some values partially fixed. In wavelet transform analyses, the *χ*(*k*) obtained from Athena was imported into the Matlab code for data computation and plotting data. The parameters were as follows: *R*‒range, 0‒10 Å; *K*‒range, 0‒10 Å^-1^; *k* weight = 3, and Morlet function with *κ* = 8, *σ* = 1 was employed as the mother wavelet to generate the overall distribution.^[5]^

**Computational Details**

All calculations were executed within the density functional theory framework using the projector-augmented plane‒wave method, implemented in the Vienna ab initio simulation package.^[6]^ The exchange‒correlation potential was determined using the generalized gradient approximation proposed by Perdew, Burke, and Ernzerhof.^[7]^ The Grimme D3 correction employed a dispersion correction that was dependent on the coordination number.^[8]^ A cut‒off energy of 550 eV was applied for the plane wave. The energy criterion in the iterative solution of the Kohn‒Sham equation was set to 10^-5^ eV. To prevent artificial interactions between periodic images, a perpendicular vacuum layer of 15 Å was added to the sheet. Brillouin zone integration was carried out using a 3×3×1 *k*‒mesh.^[9]^ All structures were relaxed until the residual forces on the atoms decreased to less than 0.03 eV Å^-1^. The free energy changes (*ΔG*) of reaction intermediates could be calculated as follows:

*ΔG* = *ΔE* + *ΔEZPE* − *TΔS*

In the equation, *ΔE* represented the adsorption energy on the cluster surface obtained from DFT calculations. *ΔEZPE* and *ΔS* denoted the differences in zero-point energy and entropy, respectively. The zero-point energy and entropy were computed under standard conditions corresponding to a pressure of 101325 Pa (~1 bar) of H_2_ at a temperature of 298.15 K.

**Cell culture**

The 4T1 cells were procured from the Institute of Biochemistry and Cell Biology, Chinese Academy of Sciences, Shanghai, which had checked the quality of the cell line (including regarding morphology and the presence of bacteria, fungi, chlamydia, and mycoplasma). The 4T1 cells were cultivated in (RPMI)‒1640 medium supplemented with 10% fetal bovine serum, 100 *μ*g mL^-1^ penicillin, and 100 *μ*g mL^-1^ streptomycin within a CO_2_ incubator (Thermo Fisher) set at a temperature of 37℃ with a carbon dioxide concentration of 5%.

**Cell phagocytosis experiment**

The 4T1 cells were spread over the T25 cell culture flasks, and the culture solution contained A‒FeO_1-x_OH (250 *μ*g mL^-1^) and 1640 was added to co‒culture with the cells, and after a period of incubation time (0 h, 1 h, 2 h, and 3 h), the culture solution was poured off, and after that, the cells were digested with tryptic EDTA solution after being washed with PBS, and were fixed in glutaraldehyde electron microscope fixative, and ultrathin electron microscope sections were prepared for observing the phagocytosis of A‒FeO_1-x_OH by the cells under the Bio‒TEM.

**The cytotoxicity assays**

The 4T1 cells were cultured in 96‒well plates and incubated with 1640 culture medium containing Fe_2_O_3_, FeCl_2_, FeO_1-x_OH, A‒FeO_1-x_OH with different concentrations (250 *μ*g mL^-1^, 175 *μ*g mL^-1^,125 *μ*g mL^-1^, 50 *μ*g mL^-1^, 25 *μ*g mL^-1^), and allicin (5 g L^-1^, 0.5 g L^-1^, 50 mg L^-1^, 5 mg L^-1^, 0.5 mg L^-1^) for 24 h. The cell survival rate was detected by a CCK‒8 kit and microplate reader.

**The CLSM observation**

The 4T1 cells were spread over a 15 mm confocal round dish, followed by adding a solution contained A‒FeO_1-x_OH (250 *μ*g mL^-1^), allicin (0.5 g L^-1^), or FeO_1-x_OH (250 *μ*g mL^-1^), respectively. After 3 h, the culture medium was poured out. After washed with PBS, gently, the PBS contained DCFH‒DA (1 *μ*M) was added for ROS observation, and Calcein AM/PI (5 *μ*M) stain was added for live/dead cells observation. The C11‒bodipy^581/591^ (10 *μ*M) was added to observe the cellular lipid peroxidation. Add EdU, Hoechst (10 *μ*M) to observe intracellular DNA repression status.

For the observation of intracellular Fe^2+^ and Fe^3+^ distribution, the identical culture solution containing A‒FeO_1-x_OH (250 *μ*g mL^-1^), FeCl_2_ (250 *μ*g mL^-1^), or FeCl_3_ (250 *μ*g mL^-1^) was added dropwise onto a 15 mm confocal round dish filled with 4T1 cells for 3 h. Subsequently, the culture solution was poured out, and the cells were gently washed with PBS, FerroOrange (1 *μ*M) and Rhodamine B (1 *μ*M) dyes were added to observe the distribution of Fe^2+^ and Fe^3+^ in the cells. Gently rinsed with PBS prior to observation.

**Testing of intracellular iron content in different valence states**

To observe the distribution of Fe^2+^ and Fe^3+^ within the cells, after spreading the cells all over the 6‒well plate, the medium was changed to PBS containing A‒FeO_1-x_OH (250 *μ*g mL^-1^), FeCl_2_ (250 *μ*g mL^-1^), or FeCl_3_ (250 *μ*g mL^-1^), and the cells were rinsed with deionized water after 0 h, 1 h, 2 h, and 3 h, after which the cells were ultrasonically crushed. The supernatant was collected by centrifugation for IC‒ICP‒OES measurement.

**The flow cytometry analysis**

The 4T1 cells were spread in 6‒well plates. Afterwards, 1640 culture medium containing A‒FeO_1-x_OH (250 *μ*g mL^-1^), allicin (0.5 g L^-1^), or FeO_1-x_OH (250 *μ*g mL^-1^) was added, respectively. The culture medium was poured out after 3 h. After washed with PBS, gently, the trypsin‒EDTA solution was added to digest, then, centrifuged and redispersed the cells. According to the kit instructions, the Annexin V‒FITC/PI was added to stain cells for 15 min. Then, PBS was immediately used to wash the cells, and flow cytometry was performed to analyze the level of apoptosis, while the DCFH‒DA dye was used to stain for the ROS analysis. EdU (5 *μ*M) was added for flow cytometry analysis to observe the intracellular DNA repression status.

**The Real‒time fluorescence quantitative PCR**

The 4T1 cells were spread in 6‒well plates, and 1640 culture medium contained A‒FeO_1-x_OH (250 *μ*g mL^-1^), Allicin (0.5 g L^-1^), or FeO_1-x_OH (250 *μ*g mL^-1^) was added, respectively. After incubated for 12 h, the culture solution was poured out, and the cells were rinsed with PBS for RT‒qPCR measurement. The primers used in measurement are listed in **Table S4.**

**The MDA and ATP assays**

The 4T1 cells were spread over 6‒well plates and divided into four groups: PBS group (control), allicin group (0.5 g L^-1^), A‒FeO_1-x_OH (250 *μ*g mL^-1^) group, and FeO_1-x_OH (250 *μ*g mL^-1^) group. The intracellular MDA as well as ATP content of the cells after treatment with the different nanomedicine solutions for 12 h were measured using MDA and ATP kits, respectively.

**The WB experiments**

The 4T1 cells were spread over 6‒well plates. The 1640 culture medium was used as co‒culture media (A‒FeO_1-x_OH (250 *μ*g mL^-1^), allicin (0.5 g L^-1^), and FeO_1-x_OH (250 *μ*g mL^-1^)) with different concentrations. After the 4T1 cells was co‒cultured with the nanodrug solution for 24 h, the WB was performed.

**The cell invasion assay**

The 4T1 cells were seeded in the upper chamber using transwell plates (Corning Inc. New York, NY, USA), and the lower layer was supplemented with culture medium contained allicin (0.5 g L^-1^), FeO_1-x_OH (250 *μ*g mL^-1^), or A‒FeO_1-x_OH (250 *μ*g mL^-1^). Used polycarbonate membrane to lay a layer of matrix gel in the upper chamber to simulate extracellular matrix. In 12 h, stained the cells entering the lower chamber with crystal violet and took photos for counting.

**The Bio‒TEM observation**

The 4T1 cells were placed into T25 cell culture bottles, and the 1640 culture medium contained A‒FeO_1-x_OH (250 *μ*g mL^-1^), allicin (0.5 g L^-1^), or FeO_1-x_OH (250 *μ*g mL^-1^) was added to the bottles. After incubation for 12 h, the culture medium was poured out and rinsed with PBS, gently. The cells were then digested with trypsin‒EDTA solution, centrifuged, redispersed, and fixed with glutaraldehyde electron microscope fixative. Ultrathin sections were prepared for observation under Bio‒TEM.

**Assessment of the role of innate immunosuppression at the intracellular level**

RAW2647 macrophages were analyzed using transwell plates (Corning Inc. New York, NY, USA). RAW264.7 macrophages were co‒incubated with 4T1 cancer cells in A‒FeO_1-x_OH (250 *μ*g mL^-1^), allicin (0.5 g L^-1^), or FeO_1-x_OH (250 *μ*g mL^-1^) for 24 h. The innate immune activation of macrophages was investigated. 1×10^5^ 4T1 cancer cells were implanted into the lower chamber of the transwell system, and 1×10^5^ RAW264.7 macrophages were implanted into the upper chamber. A‒FeO_1-x_OH (250 *μ*g mL^‒1^), allicin (0.5 g L^-1^), or FeO_1-x_OH (250 *μ*g mL^-1^) was added to the lower chamber and co‒cultured for 24 h. Crystalline violet staining of RAW264.7 macrophages in the upper chamber was performed as indicator to detect the chemotaxis index. Subsequently, macrophages from the upper chamber were collected by centrifugation and subjected to various analyses including WB, flow cytometry and ELISA according to standard protocols.

**Animal experiments**

**In vivo biosafety evaluation for animal experiments**

All animal experiments were obtained approval by the Laboratory Animal Center of Tongji University and adhered to the policies outlined by the National Ministry of Health. Healthy seven-week-old female mice with an approximate weight of 25 g were procured from Beijing Weitong Lihua Laboratory Animal Technology Co, Ltd (China). Subsequently, 28 four‒week‒old ICR mice were randomly allocated into four groups, with each cage housing 5 mice. The control group (normal saline, 10 mg kg^-1^), allicin group (10 mg kg^-1^), FeO_1-x_OH group (10 mg kg^-1^), and A‒FeO_1-x_OH group (10 mg kg^-1^) were injected with corresponding nanomedicine (200 *μ*L) at caudal vein to perform in vivo biosafety evaluation. Regularly measured the weight of mice. After 30‒day evaluation period, the blood was taken from the orbit for biochemical analysis. All ICR mice were euthanized by painless cervical dislocation, and the hearts, livers, spleens, lungs, and kidneys were dissected for systematic pathological analysis.

**Animal model construction**

Four‒week‒old female Balb/c mice were purchased from the Weitong Lihua company (China Shanghai). Each cage was cultivated 5 mice. Injected the 4T1 cell suspension (2 × 10^6^ 4T1 cells were dispersed in 100 *μ*L 1640 medium) subcutaneously into the thigh. The lengths and widths of the tumors were measured using a vernier caliper, and the tumor volumes were calculated using the following formula:

$$\text{V}_{\text{i}}\text{ = }\frac{\text{L}_{\text{i}}\text{W}_{\text{i}}^{\text{2}}}{\text{2}}$$

*V_i_*, *L_i_*, and *W_i_* represent the volume, length, and width of the tumor after *i* days, respectively. The mice were euthanized by painless cervical dislocation when the tumor volume reached 1500 mm^3^, and the tumor length in either direction can’t exceed 15 mm.

**A‒FeO_1-x_OH tumor microenvironment enrichment evaluation**

The experiments were conducted after the mouse tumor volume reached 200 mm^3^. A‒FeO_1-x_OH was conjugated with the Cy5.5 infrared group and administered via injection into the tail vein (10 mg kg^-1^, 200 *μ*L) of 4T1 tumor-bearing mice. Subsequently, the mice were subjected to in vivo fluorescence imaging at a wavelength of 703 nm at various time points (pre‒, post‒, 15 min, 30 min, 60 min, 1 h, 2 h, 4 h, 6 h, 12 h, 24 h). After 24 h, the mice were dissected, and fluorescence images of the heart, liver, spleen, lungs, and kidneys were observed, with the fluorescence intensity being evaluated.

**Tumor treatment effect evaluation and slice observation**

Tumor treatment commenced once the tumor volume reached 100 mm³. Twenty Balb/c mice were randomly divided into four groups: control group (injected with normal saline, 10 mg kg^-1^), allicin group (10 mg kg^-1^), FeO_1-x_OH group (10 mg kg^-1^), and A‒FeO_1-x_OH group (10 mg kg^-1^). The drug solution was administered through the tail vein in days 0, 4, 8, and 12, respectively. Tumor lengths and widths, as well as the weights of the mice, were measured daily. After 16 days, blood and serum were collected from the orbit for routine blood biochemistry tests. The tumors were photographed and weighed, and the hearts, livers, spleens, lungs, kidneys, and tumors were dissected to create H&E-stained sections. Additionally, H&E, GPX4, Ki67, TUNEL, and DHE‒stained sections of the tumor tissues were photographed and observed.

**Ex vivo analysis of immune response**

To perform in vitro immune response analysis, the following steps were taken. After group treatment, tumor tissues were collected in order to explore the maturation process of DCs in depth. The samples were processed to make single-cell suspensions and stained with PE anti-mouse CD11c, FITC anti‒mouse CD86 and APC anti‒mouse CD80 antibodies. Subsequently, the staining patterns of these cells were analyzed using flow cytometry to assess the immune response. In addition, while undergoing group treatment, we euthanized the mice in order to fully study their immune responses. We collected subcutaneous tumors and prepared them into single‒cell suspensions. Specifically, tumor tissues were cut into tiny pieces and digested for 45 min at 37°C in a digest containing collagenase IV (200 U mL^-1^) and DNase I (40 U mL^-1^). Afterwards, the digest was gently ground and filtered to obtain a cell suspension. Finally, cells were collected by centrifugation and stained using FITC anti‒mouse CD3, APC anti‒mouse CD4, APC anti‒mouse CD8, and PE anti‒mouse CD38 antibodies. Ultimately, we analyzed the staining results using flow cytometry to reveal the specifics of the immune response.

**Anti‒tumor recurrence therapy**

Similarly, after 15 days of tumor growth in the 4T1 tumor model mice, half of the tumor was removed and sutured the incision. The mice were injected in days 16, 18, and 20, respectively, and treatment initiated. The treatment group was divided into four groups, each injected with 200 *μ*L of drug solution, namely control group (normal saline, 10 mg kg^-1^), allicin group (10 mg kg^-1^), FeO_1-x_OH group (10 mg kg^-1^) and A‒FeO_1-x_OH group (10 mg kg^-1^). Body weights of mice were recorded throughout the entire 30‒day cycle. In the 30th day, the mice were dissected and the tumors were weighed to assess the anti‒tumor recurrence effect.

**Observation of mouse survival time**

Similar to the therapeutic effect evaluation experiment, the experiment was conducted when the tumor volume reached 200 mm^3^. Observed the survival time of mice in 0, 4, 8, and 12 days after intravenous injection of drug solution. When the tumor volume reached 1500 mm^3^, euthanized the mice.

**Statistical analysis**

The probability p was calculated using one‒way variance (ANOVA) method. For the experimental data, *p < 0.05, **p < 0.01, and ***p < 0.001.

**Reference**

[1] a) Q. Liu, L. Shi, Y. Liao, X. Cao, X. Liu, Y. Yu, Z. Wang, X. Lu, J. Wang, *Adv. Sci.* **2022**, *9*, e2200005; b) J. Guan, C. Li, J. Zhao, Y. Yang, W. Zhou, Y. Wang, G.-R. Li, *Appl. Catal. B* **2020**, *269*, 118600.

[2] P. E. Hansen, *Molecules* **2021**, *26*, 2409.

[3] E. R. T. Tiekink, *Coordin. Chem. Rev.* **2017**, *345*, 209.

[4] H. Funke, M. Chukalina, A. C. Scheinost, *J. Synchrotron Radiat.* **2007**, *14*, 426.

[5] H. Funke, A. C. Scheinost, M. Chukalina, *Phys. Rev. B* **2005**, *71*, 12.

[6] G. Kresse, D. Joubert, *Phys. Rev. B* **1999**, *59*, 1758.

[7] J. P. Perdew, K. Burke, M. Ernzerhof, *Phys. Rev. Lett.* **1997**, *78*, 1396.

[8] S. Grimme, J. Antony, S. Ehrlich, H. Krieg, *J. Chem. Phys.* **2010**, *132*, 154104.

[9] H. J. Monkhorst, J. D. Pack, *Phys. Rev. B* **1976**, *13*, 5188.
